# Synthesis and Neurotropic Activity of New Heterocyclic Systems: Pyridofuro[3,2-*d*]pyrrolo[1,2-*a*]pyrimidines, Pyridofuro[3,2-*d*]pyrido[1,2-*a*]pyrimidines and Pyridofuro[3′,2′:4,5]pyrimido[1,2-*a*]azepines

**DOI:** 10.3390/molecules26113320

**Published:** 2021-06-01

**Authors:** Samvel N. Sirakanyan, Domenico Spinelli, Athina Geronikaki, Victor Kartsev, Elmira K. Hakobyan, Anthi Petrou, Ruzanna G. Paronikyan, Ivetta M. Nazaryan, Hasmik H. Akopyan, Anush A. Hovakimyan

**Affiliations:** 1 Scientific Technological Center of Organic and Pharmaceutical Chemistry of National Academy of Science of Republic of Armenia, Institute of Fine Organic Chemistry of A.L. Mnjoyan, Ave. Azatutyan 26, Yerevan 0014, Armenia; hakobyan.elmira@mail.ru (E.K.H.); paronikyan.ruzanna@mail.ru (R.G.P.); ivetta51@yandex.com (I.M.N.); hakobyanh2021@mail.ru (H.H.A.); aaa.h.87@mail.ru (A.A.H.); 2 Dipartimento di Chimica G. Ciamician, Alma Mater Studiorum-Università di Bologna, Via F. Selmi 2, 40126 Bologna, Italy; 3 School of Pharmacy, Aristotle University of Thessaloniki, 54124 Thessaloniki, Greece; anthi.petrou.thessaloniki1@gmail.com; 4 InterBioScreen, a/ya 218, 119019 Moscow, Russia; vkartsev@ibscreen.chg.ru

**Keywords:** furo[3,2-*d*]pyrrolo[1,2-*a*]pyrimidines, furo[3,2-*d*]pyrido[1,2-*a*]pyrimidines, furo[3′,2′:4,5] pyrimido [1,2-*a*]azepines, neurotropic activity, anticonvulsant action

## Abstract

Background: Neurotic disturbances, anxiety, neurosis-like disorders, and stress situations are widespread. Benzodiazepine tranquillizers have been found to be among the most effective antianxiety drugs. The pharmacological action of benzodiazepines is due to their interaction with the supra-molecular membrane GABA-a-benzodiazepine receptor complex, linked to the Cl-ionophore. Benzodiazepines enhance GABA-ergic transmission and this has led to a study of the role of GABA in anxiety. The search for anxiolytics and anticonvulsive agents has involved glutamate-ergic, 5HT-ergic substances and neuropeptides. However, each of these well-known anxiolytics, anticonvulsants and cognition enhancers (nootropics) has repeatedly been reported to have many adverse side effects, therefore there is an urgent need to search for new drugs able to restore damaged cognitive functions without causing significant adverse reactions. Objective: Considering the relevance of epilepsy diffusion in the world, we have addressed our attention to the discovery of new drugs in this field Thus our aim is the synthesis and study of new compounds with antiepileptic (anticonvulsant) and not only, activity. Methods: For the synthesis of compounds classical organic methods were used and developed. For the evaluation of biological activity some anticonvulsant and psychotropic methods were used. Results: As a result of multistep reactions 26 new, five-membered heterocyclic systems were obtained. PASS prediction of anticonvulsant activity was performed for the whole set of the designed molecules and probability to be active Pa values were ranging from 0.275 to 0.43. The studied compounds exhibit protection against pentylenetetrazole (PTZ) seizures, anti-thiosemicarbazides effect as well as some psychotropic effect. The biological assays evidenced that some of the studied compounds showed a high anticonvulsant activity by antagonism with pentylenetetrazole. The toxicity of compounds is low and they do not induce muscle relaxation in the studied doses. According to the study of psychotropic activity it was found that the selected compounds have an activating behavior and anxiolytic effects on the models of “open field” and “elevated plus maze” (EPM). The data obtained indicate the anxiolytic (anti-anxiety) activity of the derivatives of pyrimidines, especially pronounced in compounds **6n**, **6b**, and **7c**. The studied compounds increase the latent time of first immobilization on the model of “forced swimming” (FST) and exhibit some antidepressant effect similarly to diazepam. Docking studies revealed that compound **6k** bound tightly in the active site of GABA_A_ receptor with a value of the scoring function that estimates free energy of binding (ΔG) at −7.95 kcal/mol, while compound **6n** showed the best docking score and seems to be dual inhibitor of SERT transporter as well as 5-HT_1A_ receptor. Conclusions: Тhe selected compounds have an anticonvulsant, activating behavior and anxiolytic effects, at the same time exhibit some antidepressant effect.

## 1. Introduction

Nowadays, neurotic disturbances, anxiety, neurosis-like disorders and stress situations are widespread. Benzodiazepine tranquillizers have been found to be among the most effective antianxiety drugs. The pharmacological action of benzodiazepines is due to their interaction with the supra-molecular membrane GABA-a-benzodiazepine receptor complex, linked to the Cl-ionophore. Benzodiazepines enhance GABA-ergic transmission and this has led to a study of the role of GABA in anxiety. The search for anxiolytics and anticonvulsive agents has involved glutamate-ergic, 5HT-ergic substances and neuropeptides.

Literature data evidenced that derivatives of fused pyrrolo[1,2-*a*]pyrimidines, pyrido[1,2-*a*]pyrimidines and pyrimido[1,2-*a*]azepines have a wide spectrum of biological activities such as anticonvulsive [1], antitubercular [2], bronchodilator [3] activities and HIV integrase inhibitory [4,5].

**Figure 1:**
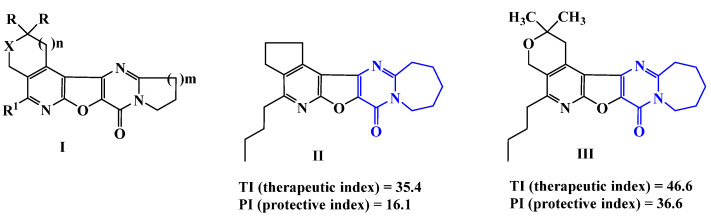
General **I** and lead structure **II**, **III** of compounds from our previous study. **I**. X = CH_2_, O; R = H, Me; n = 0, 1; R^1^ = alkyl, aryl; m = 1, 3.

In a previous paper [6] we have described the synthesis as well as the anticonvulsive and psychotropic properties of a series of furo[3,2-*d*]pyrrolo[1,2-*a*]pyrimidines and furo[3′,2′:4,5]pyrimido [1,2-*a*]azepines **I**. The results of these previous studies have enabled us to identify several compounds that exhibited potent and wide-spectrum anticonvulsant properties in the maximal electroshock seizure (MES) and the subcutaneous pentylentetrazole (scPTZ) seizure tests. Furthermore, the compounds were studied for their anxiolytic activity in some psychotropic models such as “open field” and elevated plus-maze (EPM). Moreover, our studies have shown that two derivatives among the pyridofuropyrimido[1,2-*a*]azepines **II** and **III** exhibited anticonvulsant activity significantly better than that of commercial drug zarontin (Figure 1) [6].

Based on above considerations we now design new compounds with the aim to study the influence of the homologous series on biological effect. Moreover, PASS prediction supported our idea. Continuing our studies in the field, in this paper we present the synthesis of 26 new derivatives of the above mentioned systems **I** as well as new heterocyclic systems: pyrido[3′,2′:4,5]furo[3,2-*d*]pyrrolo[1,2-*a*]pyrimidines, (m = 1), pyido[3′,2′:4,5]furo[3,2-*d*]pyrido[1,2-*a*]pyrimidines (m = 2) and pyrdo[3″,2″:4′,5′]furo[3′,2′:4,5] pyrimido[1,2-*a*]azepines (m = 3).

## 2. Results and Discussion

### 2.1. Chemistry

**Figure 2:**
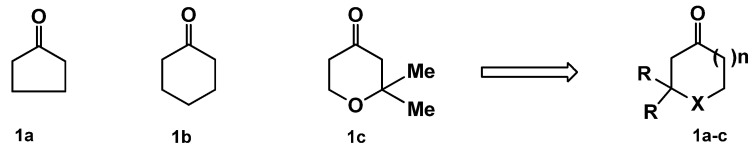
The structure of starting cyclic ketones **1a**–**c**. **1a**: X = CH_2_, n = 0, R = H, **b**: X = CH_2_, n = 1, R = H, **c**: X = O, n = 1, R = Me.

For the synthesis of the aimed compounds we used as starting materials cyclopentanone **1a**, cyclohexanone **1b**, and 2,2-dimethyltetrahydro-4*H*-pyran-4-one **1c** (Figure 2).

**Scheme 1:**
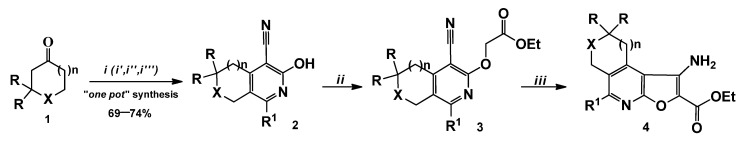
*Reagents and conditions*: i (**i’**) morpholine, C_6_H_6_, TsOH, reflux 5 h, (**i’’**) RCOCl, Et_3_N, 35–40 °C, 6 h, (i’’’) NCCH_2_CONH_2_, NH(Et)_2_, reflux 10 h; ii ClCH_2_COOEt, K_2_CO_3_/DMF, 75–80 °C, 2 h; iii EtONa, reflux 20 min. **2**–**4. a**–**d**: X = CH_2_, n = 0, R = H, **a**: R^1^ = *i*-Pr; **b**: R^1^ = *n*-Bu; **c**: R^1^ = C_6_H_11_; **d**: R^1^ = 2-furyl; **e**–**h**: X = CH_2_, n = 1, R = H, **e**: R^1^ = Me; **f**: R^1^ = Et; **g**: R^1^ = *i*-Pr; **h**: R^1^ = *i*-Bu; **i**–**o**: X = O, n = 1, R = Me, **i**: R^1^ = Me; **j**: R^1^ = Et; **k**: R^1^ = *i*-Pr; **l**: R^1^ = *n*-Bu; **m**: R^1^ = *i*-Bu; **n**: R^1^ = Ph; **o**: R^1^ = 2-furyl.

For the first time a “one-pot” synthetic method for the preparation of new as well as already known 3-oxo derivatives of cyclopenta[*c*]pyridine **2a**–**d**, of 5,6,7,8-tetrahydroisoquinoline [7,8] **2e**–**h** and of pyrano[3,4-*c*]pyridine [9] **2i**–**o** was developed and is described. Thus, the reaction of ketones **1** with morpholine in anhydrous benzene led to the formation of the corresponding enamines. After, the acylation of the enamines, followed by the cyclocondensation intermediate compounds with 2-cyanoacetamide in the presence of diethylamine led to the formation of the aimed compounds **2a**–**o** in good to high yields (Scheme 1). This approach of synthesis for compounds **2** was not described in the literature but, although no significant difference was observed in terms of yields compared to the already described multistep synthesis of these compounds **2** [7,8,9], it has some advantages: it reduces the time required to obtain these compounds, requires fewer reagents and solvents, and is less laborious.

The structure of newly obtained compounds **2a**–**d**,**g**,**h** (the physico-chemical characterization of compounds **2e**,**f** [7,8] and **2i**–**o** [9] were already reported) was confirmed by NMR, IR spectroscopy and by elemental analysis. Thus, in the ^1^H-NMR spectra of these new compounds **2a**–**d**,**g**,**h** the presence of the NH group proton at 12.05−12.19 ppm was observed. The IR spectra of **2a**–**d**,**g**,**h** show carbonyl group absorptions near 1630−1647 cm^−1^, nitrile groups near 2220−2228 cm^−1^, and NH groups in the region 3117−3142 cm^−1^ (see Supplementary Material).

All these starting materials **2** are versatile substrates: in fact, all of them are decorated on adjacent carbon atoms of the pyridine ring by two functional groups able to open the way to compounds with a new condensed ring (a furan ring), which in turn will still contain other reactive groups useful for further chemical transformations.

Thus, compounds **2a**–**o** by interaction with ethyl chloroacetate in the basic medium were converted into the corresponding *O*-alkylated compounds **3a**–**o** [6,10,11,12,13]. Then, compounds **3** underwent cyclization in the presence of sodium ethoxide giving the fused furo[2,3-*b*]pyridines **4a**–**o** [6,10,11,12,13] by the Thorpe-Ziegler reaction (Scheme 1).

**Scheme 2:**
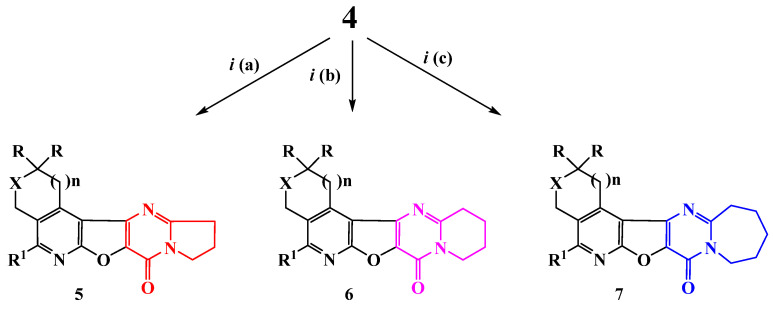
*Reagents and conditions*: *i*−2-pyrrolidone (**a**), 2-piperidone (**b**), 2-azepanone (**c**), POCl_3_, C_2_H_4_Cl_2_, reflux 25 h.

The obtained 1-aminofuro[2,3-*b*]pyridine-2-carboxylates **4** still contain functional groups which can undergo new cyclization reactions. Thus, the reaction of compounds **4** with some lactams: 2-pyrrolidinone, 2-piperidone, 2-azepanone, in the presence of phosphorus oxychloride led to the simultaneous ring closing reaction of the two heterocycles [1,2,6,14] with formation of new heterocyclic systems: pyrido[3′,2′:4,5]furo[3,2-*d*]pyrrolo[1,2-*a*]pyrimidines **5a**–**d**, pyrido[3′,2′:4,5]furo[3,2-*d*]pyrido[1,2-*a*]pyrimidines **6a**–**n** and pyrido[3″,2″:4′,5′]furo[3′,2′:4,5]pyrimido[1,2-*a*]azepines **7a**–**h** in good yields (Scheme 2, Table 1).

The structure of new synthesized compounds **5**–**7** was supported by NMR and IR spectroscopy. Thus, the IR spectra of compounds **5**–**7** did not show the characteristic bands of the amino group, but showed the bands in the range of ν 1683–1702 cm^−1^ typical for the carbonyl group. In the ^1^H-NMR spectra the singlet signals of NH_2_ and ester COOEt groups characteristic for the initial compounds **4** were absent, while the 3, 4 and 5-CH_2_ group signals appeared, respectively, indicating the cyclization of compounds **4**. The structure of compounds **5**–**7** was also supported by ^13^C-NMR data (see Supplementary Material).

**Figure 3:**
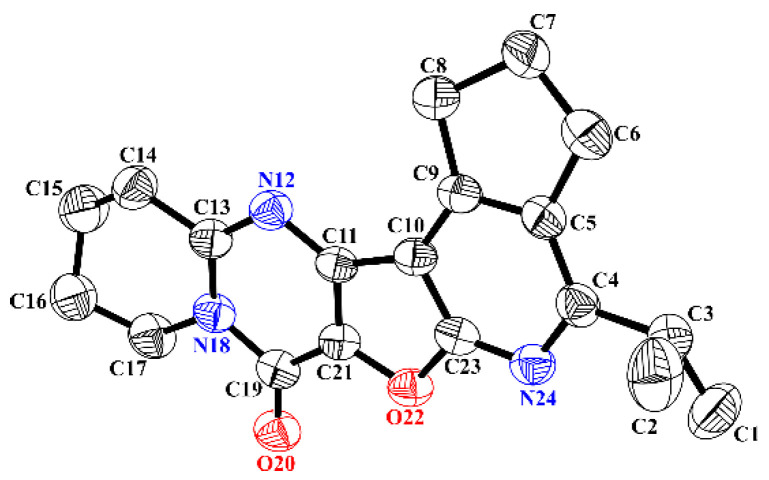
Single crystal X-ray structure of compound **6a**.

As the polycyclic system of pyrido[3′,2′:4,5]furo[3,2-*d*]pyrido[1,2-*a*]pyrimidines **6** was never described in the literature, their structure was also confirmed by the X-ray analysis in the instance of compounds **6a** (Figure 3).

### 2.2. PASS Prediction

PASS prediction of anticonvulsant activity was performed for the whole set of the designed and synthesized molecules. Anticonvulsant activity was predicted with the probability to be active Pa values ranging from 0.275 to 0.43 (Table 2). The calculated Pa values for most of the compounds were less than 0.5, indicating their relative novelty compared to the structures of the compounds from the PASS training set [15,16]. Such results lead to the conclusion that the studied compounds have some features different from those of well-known anticonvulsant agents, which may indicate their innovative potential.

### 2.3. Biological Assay

The neurotropic activity of 26 new synthesized heterocyclic compounds: pyrido[3′,2′:4,5]furo[3,2-*d*]pyrrolo[1,2-*a*]pyrimidines **5a**–**d**, pyrido[3′,2′:4,5]furo[3,2-*d*]pyrido[1,2-*a*]pyrimidines **6a**–**n** and pyrido[3″,2″:4′,5′]furo[3′,2′:4,5]pyrimido[1,2-*a*]azepines **7a**–**h** was investigated according to indicators characterizing anticonvulsant, sedative, anti-anxiety activities and side effects.

The study of the anticonvulsant activity of compounds **5**–**7** was assessed by evaluating the antagonism to the convulsive pentylenetetrazole (PTZ) action and maximal electroshock seizures (MES) [17,18,19,20,21,22]. PTZ induced test is considered an experimental model for the clonic component of epilepsy seizures and prognostic anxiolytic activities of the compounds. The MES test is used as an animal model for the generalization of tonic seizures of epilepsy. The side effects of the compounds–neurotoxicity (movement coordination disorder, myorelaxation and ataxia) by using the test of “rotating rod” [17,23] was also studied on mice.

To determine the 50% effective (ED_50_, causing the anticonvulsant effect of 50% of animals, is calculated by the test antagonism of PTZ), 50% neurotoxic (TD_50_, causing myorelaxation effect in 50% of animals, is calculated by the test “rotating rod”) and 50% lethal (LD_50_, causing death in 50% of animals) doses a statistical method of probit analysis as proposed by Litchfield and Wilcoxon [24,25] were used. From a practical point of view for the active compounds therapeutic (TI = LD_50_/ED_50_) indexes were identified. The well-known antiepileptic drug ethosuximide and the tranquilizer diazepam were used as reference drugs [26].

The evaluation of anticonvulsant activity of synthesized compounds **5**–**7** was performed by PTZ. The results of PTZ test showed that anticonvulsant activity was ranged from 20 to 80. Then their therapeutic (TI) indexes are much greater than that of reference drug ethosuximide (Table 3).

The ED50 and TD50 data were used to calculate protective index (PI), which is a measure of the benefit-risk proportion of the therapeutic agent. Results of the tested compounds with the data for reference (ethosuximide (ETX) and diazepam are presented in Table 3. It can be seen that all compounds exhibited almost three-to-four-fold lower ED50 value than ethosuximide. Compound **6k** showed the highest activity among all tested higher than ethosuximide with ED50 30 mg/kg and TD50 >200, resulted in PI value of 6.7. In general, all compounds showed PI index superior than ethosuximide and almost equal to diazepam.

Thus, activity for the group of pyrido[3′,2′:4,5]furo[3,2-*d*]pyrido[1,2-*a*]pyrimidines can be presented as follows: **6k** > **6j** > **6n**. Interestingly, compound **7h** showed activity similar to that of **6k**. The difference between these compounds is that **7h** is pyrimido[1,2-*a*]azepine and **6k** is pyrido[1,2-*a*]pyrimidine derivative as well as in substitution at 5 position. It seems that *n*-Bu and 2-furyl substituents in position 5 of these two compounds have similar positive influence on activity.

The evaluation revealed that the studied compounds are superior to ethosuximide in anticonvulsant activity by antagonism with pentylenetetrazole, but inferior to diazepam. However, unlike diazepam, they in the studied doses (12.5, 50, 200 mg/kg) do not induce muscle relaxation and are not very toxic (LD_50_ = 450–930 mg/kg).

The six more active compounds **6b**, **6j**, **6k**, **6n**, **7c**, and **7h** were selected to study them on the model of thiosemicarbazide (TSC) seizures (affecting the exchange of GABA, Table 3). In the TSC-induced seizure model, the anticonvulsant effect is similar to that of the sc-PTZ induced seizure mod (Table 3). The compounds at dose of 100 mg/kg increased latency of thiosemicarbazide seizures to 1.3–2.3 times compared with the control. Ethosuximide has approximately the same effect increasing the latent time of TSC convulsions. Diazepam is not effective in this model.

According to the MES test, the studied compounds as well as reference drugs did not exhibit an anticonvulsant effect. They did not protect from tonic and clonic seizures, caused by MES.

Furthermore, the most effective compounds: **6b**, **6j**, **6k**, **6n**, **7c** and **7h**, were studied on the “open field” [27,28], “elevated plus maze” (EPM) [29], “forced swimming” [30,31] tests at a dose of 50 mg/kg, since the ED_50_ of these compounds are within 50 mg/kg at the confidence intervals.

In the “open field“ model [27,28] in rats of the control group, the number of horizontal, vertical displacements and the number of examined cells were 14.4, 3.4, and 0.8 respectively (Table 4). The studied compounds caused significant changes in behavioral indices compared to control. After injection of the compounds an increase in horizontal and vertical movements of rats was observed (Table 4). Furthermore, all compounds statistically significant compared with the control, especially compounds **6j** and **6n** increase the number of sniffing cell examinations, probably due to the expression of the anti-anxiety activity of the compounds. All these findings are an indication of activating effect of the studied compounds.

Diazepam showed an activating effect too, in contrast to ethosuximide, which has neither an activating nor a sedative effect at the studied dose. Compounds, like diazepam, increase the number of cells examined. The data obtained indicate the anxiolytic (anti-anxiety) activity of the pyrido[1,2-*a*]pyrimidines and pyrimido[1,2-*a*]azepines, especially pronounced in the compounds **6b**, **6j**, and **6n**. The order of anxiolytic activity of tested compounds in this model is: **6n** > **6j** > **6b** > **7h** > **7c** > **6k**, indicating the beneficial role of 2-furyl substitution in both series pyrido-pyrimidine-ones and pyrimido-azepin-ones.

The evaluation of fear was assessed using the methodology of elevated plus-maze (EPM) developed by Pellow and File [29]. The elevated plus maze is known behavioural assay (fear) used for the estimation of the anti-anxiety effect of pharmacological agents and synthetic compounds. In this model control animals are predominantly located in closed arms (Table 5). All compounds as well as ethosuximide and diazepam increased in a statistically significant manner the time spent by experimental animals in the center. Statistically the investigated compounds significantly reduce the residence time in the closed arms. After the administration of compounds **6b**, **6n** and **7c**, the experimental animals, in contrast to the control animals and those who received ethosuximide at a dose of 200 mg/kg, enter the open arms and stay there from 14 (**7c**) to 21.8 (**6n**) s. The administration of these compounds, as well as diazepam, in fact, leads to the identification of an anxiolytic effect. In this test again the most active compound was found to be **6n**, followed by **6b.**

The forced swimming test (FST) [30,31] is used to monitor depressive-like behavior and is based on the assumption that immobility reflects a measure of behavioral despair. On the “forced swimming” model, some of the investigated selected compounds increase the active swimming time and the latent period of first immobilization (188 s for compound **6k**) (Table 6). In control mice, the first immobilization occurs after 127.5 s. This indicates that the studied compounds at a dose of 50 mg/kg exhibit some antidepressant effect. The data regarding ethosuximide at a dose of 200 mg/kg are comparable with the control data. The reference drug diazepam at a dose of 2 mg/kg acts similarly to compounds tested increasing the latent time of first immobilization. At the same time, diazepam significantly reduces the total immobilization time, while the compounds **6j**, **6k**, **7h** and ethosuximide increase it. The compounds have practically no effect on the total swimming time or reduce it. According to the data of Table 5, the activity of compounds in the FST can be presented as follows: **6k > 7h > 6j > 7c > 6n > 6b.**

According to the structure-activity relationship study, in general, the introduction of the butyl and furyl group in the pyridine ring in position 4(5) of heterocyclic systems as in compounds **6b**, **6k**, **6n** and **7h**, appeared to be beneficial for neurotropic activity and are in agreement with previous obtained data [6]. From obtained results one can conclude that compound **6j** is dual acting anticonvulsant/anxiolytic, while **6k** also is dual acting anticonvulsive and antidepressant.

### 2.4. Molecular Docking

#### 2.4.1. Docking Studies for Prediction of the Mechanism of Anticonvulsant and Anxiolytic Activity (Docking to GABA_A_ Receptor)

It is well known that antiepileptic drugs target GABA_A_ receptors block sodium channels or enhance γ-aminobutyric acid (GABA) function [32,33,34]. Therefore, docking studies of all tested compounds were performed in order to get a better understanding of the GABA_A_ receptor inhibitory potency at the molecular level and to shed light on the interactions in the active site of GABA_A_ receptor.

For docking studies, the crystal structure of GABA_A_ receptor was retrieved from Protein Data Bank (PDB) with PDB ID: 4COF [33]. The X-Ray diffraction structure of GABA_A_ receptor had a resolution of 2.97 Å, R value of 0.206 and R free value of 0.226. As a first step of docking studies and for the validation of docking parameters, the initial co-crystal ligand benzamidine was re-docked at the catalytic site of protein, and the root-mean-square deviation (RMSD) between co-crystal and re-docked pose was found to be 0.34 Å.

**Figure 4:**
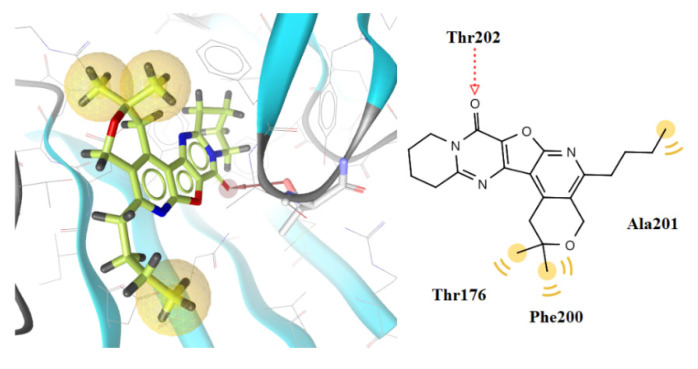
Docked pose of compound **6k** and GABA_A_ receptor complex; Red lines show the hydrogen bonds, yellow and purple-colored spheres and lines represent hydrophobic and aromatic interactions, respectively.

**Figure 5:**
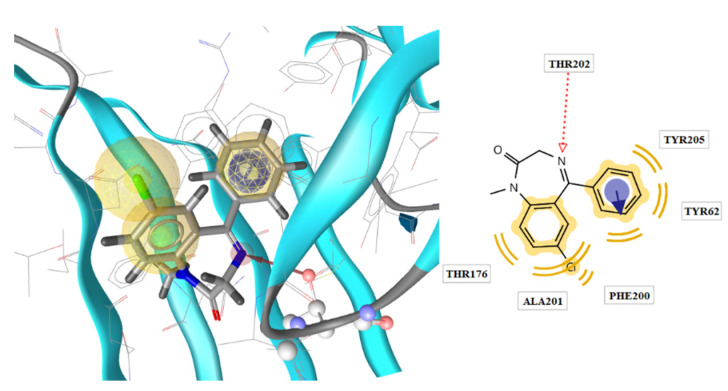
Docked conformation of diazepam and GABA_A_ receptor complex.

Docking results are represented in Table 7 and revealed that compound **6k** bound tightly in the active site of GABA_A_ receptor with a value of the scoring function that estimates free energy of binding (ΔG) at −7.95 kcal/mol, forming one hydrogen bond between the oxygen atom of C=O group and the hydrogen atom of the backbone hydrogens of the amino acid residue Thr202 (distance 1.75 Å) respectively (Figure 4). Furthermore, the CH_3_ groups of the compound showed hydrophobic interactions with the residues Thr176, Phe200 and further stabilizing the complex ligand-enzyme as it was observed for Diazepam (Figure 5). The obtained docking results, even this is only the prediction, are in accordance with the experimental.

#### 2.4.2. Docking to SERT Transporter and 5-HT_1A_ Receptor

Antidepressant drugs fall in two main categories, the tricyclic antidepressants and selective serotonin reuptake inhibitors (SSRIs) [34]. These drugs inhibit the transport of serotonin into the presynaptic neuron by inhibiting the serotonin (5-HT) transporter (SERT). SERT is a transmembrane protein located in the membrane of presynaptic neurons which removes the serotonin from the synaptic cleft resulting to termination of serotonergic neurotransmission. The increase in serotonin activates the 5-HT_1A_ receptors decrease the serotonergic neurotransmission follow-on a delay in the onset of antidepressant action [35,36]. This delay lasts until HT_1A_ receptors become desensitized and the release of serotonin is normalized.

Tacking all the above into account, in order to study if the tested compounds act as dual inhibitors of serotonin transporter (SERT) and alongside antagonize the presynaptic autoinhibitory 5-HT_1A_ receptors, we proceed on docking studies in SERT transporter as well as 5-HT_1A_ receptor.

As there is no available crystal structure of SERT transporter at Protein Data Bank (PDB) we used the X-ray crystal structure of LeuT bound to L-Tryptophan (PDB code: 3F3A), a prokaryotic homologue of SERT [37]. The results of docking studies on SERT transporter are presented in Table 8.

**Figure 6:**
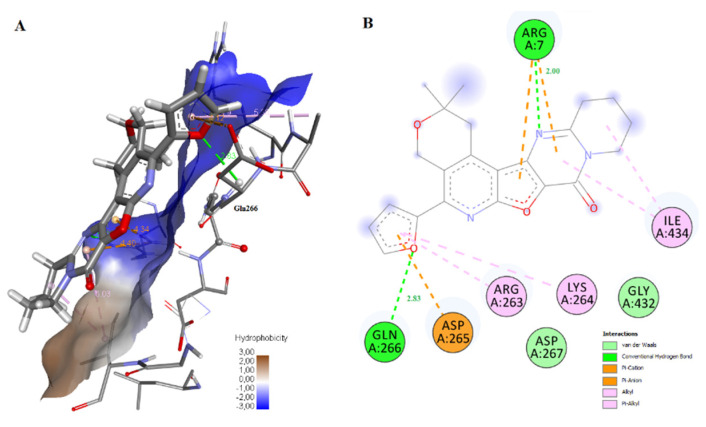
(**A**) Docked pose of compound **6n** and SERT transporter complex; (**B**) 2D ligand interaction diagram for docked ligand.

Compound **6n** by forming two hydrogen bonds showed the best docking score which is in accordance with the experimental results. The first one between the N atom of fused rings and the hydrogen atom of the side chain of Arg7 (distance 2.00 Å) and the other between the oxygen atom of furan ring of the compound and the hydrogen of the side chain of Gln266 (distance 2.83A). The fused rings showed hydrophobic interactions with the residues Arg7, Asp267, Gly433, Gly432 and Ile434, while the furan ring interact hydrophobically with the residues Asp265, Arg263 and Ly264 (Figure 6).

For docking to 5-HT_1A_ receptor the crystal structure of the human β2-adrenergic receptor in complex with the neutral antagonist alprenolol (PDB code: 3NYA) was used [38,39]. For the validation of docking parameters, the initial co-crystal ligand alprenolol was re-docked at the catalytic site of protein, and the root-mean-square deviation (RMSD) between co-crystal and re-docked pose was found to be 0.98 Å.

**Figure 7:**
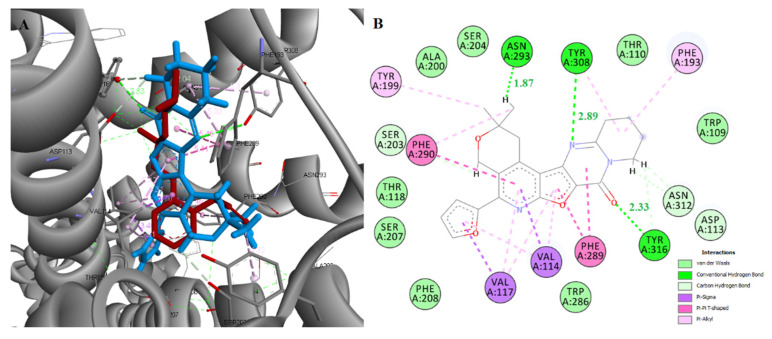
(**A**) Docked pose of compound **6n** (blue) and alprenolol (red) into 5-HT_1A_ receptor (**B**) 2D ligand interaction diagram for docked compound **6n**.

All the tested compounds were docked into the orthostatic binding site of the 5-HT_1A_ receptor (Table 9). The best docking score was achieved for compound **6n** (−10.25 kcal/mol) which formed 3 hydrogen bonds with the residues Asn293, Tyr308, and Tyr316. Additionally, hydrophobic interactions were observed between the fused rings of the compound and the residues Thr110, Phe193, Trp109, Asn312, Asp113, Phe289, Val114, Ser204, Ala200, Phe290, and Tyr199 while the furan ring interact hydrophobically with the residues Val117, Val114, Thr118, Ser207, and Ph208 (Figure 7B). It is worth to underline that alprenolol formed also hydrogen bond with the same Tyr316 residue as compound **6n**. Moreover, this compound is orientated in the same cavity into the enzyme as alprenolol does (Figure 7A). This is maybe a reason of the high action of compound **6n**. Finally, the docking studies revealed that this compound can probably be a dual target molecule, since it seems to be good inhibitor of SERT transporter and alongside good 5-HT_1A_ receptor binder.

### 2.5. Drug Likeness

According to definition “drug-likeness” assesses qualitatively the possibility for a molecule to become an oral drug with respect to bioavailability. Drug-likeness was established from structural or physicochemical inspections of development compounds advanced enough to be considered oral drug-candidates. Various rule-based filters such as viz. Lipinski, Ghose, Veber, Egan, and Muegge with diverse ranges of properties inside of which the molecule is defined as drug-like are used, measure Drug-likeness of the tested compounds according to some acute criterion like molecular weight, LogP, number of hydrogen bond acceptors and donors. The number of violations to the above-disclosed rules along with bioavailability and Drug-likeness scores are given in Table 10.

**Figure 8:**
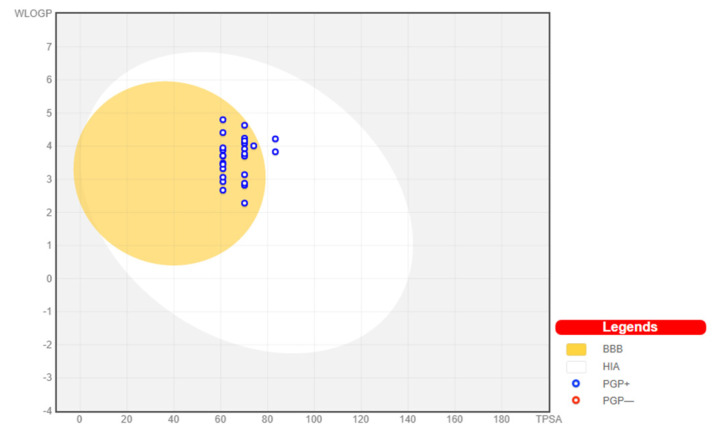
BOILED-Egg diagram of the tested compounds.

**Figure 9:**
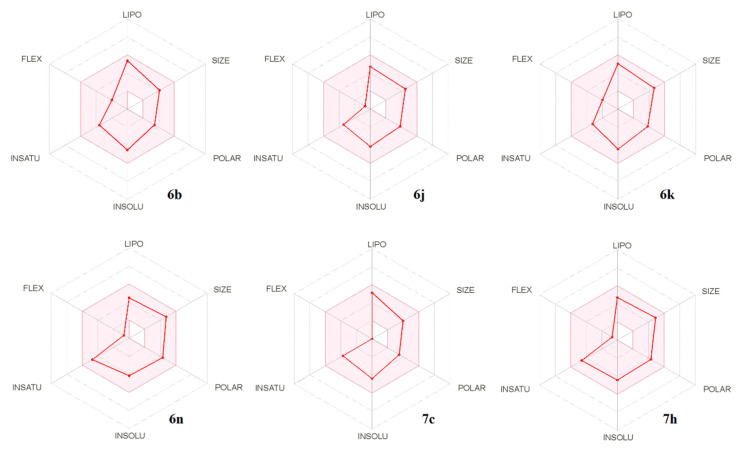
Bioavailability Radar of compounds **6b**, **6j**, **6k**, **6n**, **7c**, and **7h**. The pink area represents the optimal range for each property for oral bioavailability, (Lipophilicity (LIPO): XLOGP3 between −0.7 and +5.0, Molecular weight (SIZE): MW between 150 and 500 g/mol, Polarity (POLAR) TPSA between 20 and 130 Å^2^, Solubility (INSOLU): log S not higher than 6, Saturation (INSATU): fraction of carbons in the sp3 hybridization not less than 0.25, and Flexibility (FLEX): no more than 9 rotatable bonds.

According to obtained results none of the compounds violated any rule and their bioavailability score was around 0.55. All the tested molecules displayed high gastrointestinal (GI) absorption, are P-gp (p-glycoprotein) inhibitors, and able to pass the blood-brain barrier (BBB) except compounds **5c** and **7h**. The predictions for the passive BBB permeation, HIA (human gastrointestinal absorption), and P-gp substrates are displayed together in the BOILED-Egg diagram as shown in Figure 8. All compounds exhibited good to excellent Drug-likeness scores ranged from 0.22 to 1.01. Furthermore, the experimentally active compounds **6b**, **6j**, **6k**, **6n**, **7c**, and **7h** appeared to have good *in-silico* predictions with a Drug-likeness score ranging from 0.26 to 0.97 for compound **6b** without any violation. The bioavailability radar of these compounds is presented in Figure 9.

## 3. Materials and Methods

### 3.1. Chemistry

#### 3.1.1. General Information

^1^H- and ^13^C-NMR spectra were recorded in DMSO/CCl_4_ (1/3) solution (300 MHz for ^1^H and 75 MHz for ^13^C, respectively) on a Mercury 300VX spectrometer (Varian Inc., Palo Alto, CA, USA). Chemical shifts were reported as δ (parts per million) relative to TMS as internal standard. IR spectra were recorded on an Avatar 330-FT-IR spectrophotometer (Thermo Nicolet, CA, USA) and the reported wave numbers were given in cm^−1^. All melting points were determined in an open capillary and were uncorrected. Elemental analyses were performed on a CarloErba-1106 machine (Hewlett-Packard, Milan, Italy). Quoted values were in the range ±0.4% of the theoretical ones. Crystallographic data for the structure of compound **6a** were deposited with the Cambridge Crystallographic Data Center as supplementary publication no: CCDC 1918758. Copies of the data can be obtained, free of charge, on application to CCDC, 12 Union Road, Cambridge CB2 1EZ, UK (fax. +44-(0)1223-336033 or e-mail: deposit@ccdc.cam.ac.uk). Compounds **2e**,**f [7,8]**, **2i**–**o [9]**, **3a**,**b**,**d**–**m**,**o [6,10,11,12,13]**, and **4a**,**b**,**d**,**e**,**g**–**m**,**o [6,10,11,12,13]** were already described.

#### 3.1.2. General Method for the Preparation of Compounds **2a**–**d**,**g**,**h**

A stirred solution of ketone **1** (100 mmol), morpholine (8.7 g, 100 mmol) and catalytic amount of TsOH in anhydrous benzene (100 mL) was refluxed for 5 h. After triethylamine (10.1 g, 100 mmol) was added and the appropriate acyl chloride (100 mmol) was added dropwise maintaining the reaction temperature at 35–40 °C for 6 h. Then, cyanoacetamide (8.4 g, 100 mmol) and diethylamine (7.3 g, 100 mmol) were added and the reaction mixture was refluxed for 10 h. After cooling, the separated crystals were filtered off, washed with water, dried, and recrystallized from dimethylformamide.

*1-Isopropyl-3-oxo-3,5,6,7-tetrahydro-2H-cyclopenta[c]pyridine-4-carbonitrile* (**2a**). Colorless solid, yield 73%, mp 330−332 °C; IR *ν*/cm^−1^: 1636 (C=O), 2225 (C≡N), 3142 (NH). ^1^H-NMR δ: 1.25 (d, *J =* 7.0 Hz, 6H, CH(CH_3_)_2_), 2.03–2.14 (m, 2H, 6-CH_2_), 2.73 (t, *J =* 7.3 Hz, 2H, 7-CH_2_), 2.88 (sept., *J =* 7.0 Hz, 1H, CH(CH_3_)_2_), 2.92 (t, *J =* 7.5 Hz, 2H, 5-CH_2_), 11.83 (br s, 1H, NH). ^13^C-NMR δ: 19.33, 23.99, 28.04, 30.80, 32.75, 95.93, 114.90, 117.32, 153.24, 161.04, 167.30. Anal. calcd. for C_12_H_14_N_2_O: C 71.26; H 6.98; N 13.85%. Found: C 71.61; H 7.18; N 14.11%.

*1-Butyl-3-oxo-3,5,6,7-tetrahydro-2H-cyclopenta[c]pyridine-4-carbonitrile* (**2b**). Colorless solid, yield 71%, mp 240−241 °C; IR *ν*/cm^−1^: 1647 (C=O), 2228 (C≡N), 3137 (NH). ^1^H-NMR δ: 0.93 (t, *J* = 7.3 Hz, 3H, CH_2_CH_3_), 1.28–1.41 (m, 2H, CH_2_CH_3_), 1.49–1.61 (m, 2H, CH_2_C_2_H_5_), 2.03–2.14 (m, 2H, 6-CH_2_), 2.43–2.49 (m, 2H, CH_2_C_3_H_7_), 2.68 (t, *J* = 7.3 Hz, 2H, 7-CH_2_), 2.92 (t, *J* = 7.6 Hz, 2H, 5-CH_2_), 12.05 (br s, 1H, NH). ^13^C-NMR δ: 13.31, 21.67, 24.06, 27.82, 29.74, 30.63, 32.79, 95.64, 114.96, 118.64, 148.93, 160.79, 166.90. Anal. calcd. for C_13_H_16_N_2_O: C 72.19; H 7.46; N 12.95%. Found: C 72.51; H 7.63; N 13.18%.

*1-Cyclohexyl-3-oxo-3,5,6,7-tetrahydro-2H-cyclopenta[c]pyridine-4-carbonitrile* (**2c**). Colorless solid, yield 69%, mp 326−328 °C; IR *ν*/cm^−1^: 1643 (C=O), 2220 (C≡N), 3141 (NH). ^1^H-NMR δ: 1.20–1.28 (m, 3H, C_6_H_11_), 1.59–1.95 (m, 7H, C_6_H_11_), 2.01–2.17 (m, 2H, 6-CH_2_), 2.41–2.53 (m, 1H, CH, C_6_H_11_), 2.74 (t, *J =* 7.4 Hz, 2H, 7-CH_2_), 2.93 (t, *J =* 7.6 Hz, 2H, 5-CH_2_), 11.84 (br s, 1H, NH). ^13^C-NMR δ: 23.97, 24.59, 25.68, 28.21, 28.85, 32.80, 41.13, 95.96, 114.89, 117.68, 152.40, 161.09, 167.17. Anal. calcd. for C_15_H_18_N_2_O: C 74.35; H 7.49; N 11.56%. Found: C 74.72; H 7.70; N 11.84%.

*1-(2-Furyl)-3-oxo-3,5,6,7-tetrahydro-2H-cyclopenta[c]pyridine-4-carbonitrile* (**2d**). Yellow solid, yield 74%, mp 356−358 °C; IR *ν*/cm^−1^: 1630 (C=O), 2220 (C≡N), 3117 (NH). ^1^H-NMR δ: 2.10–2.21 (m, 2H, 6-CH_2_), 2.98 (t, *J =* 7.7 Hz, 2H, 7-CH_2_), 3.09 (t, *J =* 7.4 Hz, 2H, 5-CH_2_), 6.61 (dd, *J* = 3.6, 1.7 Hz, 1H, 4-CH_fur._), 7.50 (d, *J* = 3.6 Hz, 1H, 3-CH_fur._), 7.75 (d, *J* = 1.7, Hz, 1H, 5-CH_fur._), 12.01 (br s, 1H, NH). ^13^C-NMR δ: 24.11, 30.40, 32.81, 95.82, 112.81, 114.64, 115.79, 118.38, 134.73, 145.87, 146.57, 160.46, 168.92. Anal. calcd. for C_13_H_10_N_2_O_2_: C 69.02; H 4.46; N 12.38%. Found: C 69.36; H 4.65; N 12.64%.

*1-Isopropyl-3-oxo-2,3,5,6,7,8-hexahydroisoquinoline-4-carbonitrile* (**2g**). Light yellow solid, yield 70%, mp > 360 °C; IR *ν*/cm^−1^: 1634 (C=O), 2220 (C≡N), 3142 (NH). ^1^H-NMR δ: 1.24 (d, *J =* 6.9 Hz, 6H, CH(CH_3_)_2_), 1.72–1.78 (m, 4H, 6,7-CH_2_), 2.76–2.82 (m, 2H, 8-CH_2_), 2.92–2.98 (m, 2H, 5-CH_2_), 3.11 (sept., *J =* 7.0 Hz, 1H, CH(CH_3_)_2_), 11.82 (br s, 1H, NH). Anal. calcd. for C_13_H_16_N_2_O: C 72.19; H 7.46; N 12.95%. Found: C 72.57; H 7.68; N 13.24%.

*1-Isobutyl-3-oxo-2,3,5,6,7,8-hexahydroisoquinoline-4-carbonitrile* (**2h**). Colorless solid, yield 72%, mp 248−250 °C; IR *ν*/cm^−1^: 1651 (C=O), 2215 (C≡N), 3138 (NH). ^1^H-NMR δ: 0.94 (d, *J =* 6.7 Hz, 6H, CH(CH_3_)_2_), 1.67–1.81 (m, 4H, 6,7-CH_2_), 1.89–2.04 (m, 1H, CH(CH_3_)_2_), 2.41 (d, *J =* 7.4 Hz, 2H, CHCH_2_), 2.42–2.48 (m, 2H, 8-CH_2_), 2.76–2.82 (m, 2H, 5-CH_2_), 12.19 (br s, 1H, NH). ^13^C-NMR δ: 20.98, 21.79, 21.91, 23.44, 27.74, 28.81, 38.21, 99.35, 112.20, 114.94, 151.23, 158.97, 159.59. Anal. calcd. for C_14_H_18_N_2_O: C 73.01; H 7.88; N 12.16%. Found: C 73.32; H 8.06; N 12.40%.

#### 3.1.3. General Method for the Preparation of Compounds **3c**,**n**

To a suspension of compound **2** (10 mmol) and potassium carbonate (1.52 g, 11 mmol) in absolute DMF (30 mL) ethyl chloroacetate (1.2 mL, 11 mmol) was added dropwise under stirring. The reaction mixture was maintained at 75−80 °C for 2 h, then cooled to room temperature, and poured onto cold water. The resulting crystals were filtered off, washed with water, dried, and re-crystallized from ethanol.

*Ethyl [(4-cyano-1-cyclohexyl-6,7-dihydro-5H-cyclopenta[c]pyridin-3-yl)oxy]acetate* (**3c**). Colorless solid, yield 94%, mp 125−127 °C; IR *ν*/cm^−1^: 1761 (C=О), 2225 (C≡N). ^1^H-NMR δ: 1.27 (t, *J =* 7.1 Hz, 3H, CH_2_CH_3_), 1.23–1.55 (m, 5H, C_6_H_11_), 1.62–1.85 (m, 5H, C_6_H_11_), 2.11–2.23 (m, 2H, 6-CH_2_), 2.55–2.61 (m, 1H, CH, C_6_H_11_), 2.68 (t, *J =* 7.4 Hz, 2H, 7-CH_2_), 3.04 (t, *J =* 7.6 Hz, 2H, 5-CH_2_), 4.15 (q, *J =* 7.1 Hz, 2H, CH_2_CH_3_), 4.83 (s, 2H, OCH_2_). Anal. calcd. for C_19_H_24_N_2_O_3_: C 69.49; H 7.37; N 8.53%. Found: C 69.86; H 7.59; N 8.81%.

*Ethyl [(5-cyano-3,3-dimethyl-8-phenyl-3,4-dihydro-1H-pyrano[3,4-c]pyridin-6-yl)oxy]acetate* (**3n**). Colorless solid, yield 83%, mp 133−135 °C; IR *ν*/cm^−1^: 1748 (C=О), 2231 (C≡N). ^1^H-NMR δ: 1.24 (t, *J =* 7.1 Hz, 3H, CH_2_CH_3_), 1.36 (s, 6H, С(CH_3_)_2_), 2.94 (s, 2H, CH_2_), 4.22 (q, *J =* 7.1 Hz, 2H, CH_2_CH_3_), 4.71 (s, 2H, OCH_2_), 4.98 (s, 2H, OCH_2_CO), 7.39−7.47 (m, 5H, Ph). ^13^C-NMR δ: 13.65, 26.16, 38.09, 59.64, 60.06, 62.40, 69.19, 94.25, 112.82, 121.95, 127.71, 128.14, 128.82, 137.06, 150.94, 155.07, 160.15, 167.04. Anal. calcd. for C_21_H_22_N_2_O_4_: C 68.84; H 6.05; N 7.65%. Found: C 69.19; H 6.24; N 7.90%.

#### 3.1.4. General Method for the Preparation of Compounds **4c**,**f**,**n**

To a solution of sodium ethoxide [0.25 g (11 mmol) in absolute ethanol (30 mL)] was added compound **3** (10 mmol). The mixture was refluxed for 15−20 min, cooled, and poured onto ice. The formed crystals were filtered off, washed with water, dried, and recrystallized from ethanol.

*Ethyl 1-amino-5-cyclohexyl-7,8-dihydro-6H-cyclopenta[d]furo[2,3-b]pyridine-2-carboxylate* (**4c**). Colorless solid, yield 87%, mp 157−159 °C; IR *ν*/cm^−1^: 3443, 3347 (NH_2_), 1658 (C=O). ^1^H-NMR δ: 1.25–1.42 (m, 3H, C_6_H_11_), 1.58–1.89 (m, 7H, C_6_H_11_), 1.39 (t, *J =* 7.1 Hz, 3H, CH_2_CH_3_), 2.15–2.27 (m, 2H, 7-CH_2_), 2.64–2.74 (m, 1H, CH, C_6_H_11_), 2.91 (t, *J =* 7.4 Hz, 2H, 6-CH_2_), 3.25 (t, *J =* 7.6 Hz, 2H, 8-CH_2_), 4.31 (q, *J =* 7.1 Hz, 2H, CH_2_CH_3_), 5.53 (br s, 2H, NH_2_). ^13^C-NMR δ: 14.16, 24.37, 25.52, 25.93, 28.86, 30.67, 30.88, 43.03, 58.75, 108.58, 121.82, 131.30, 138.60, 148.63, 158.95, 160.18, 160.46. Anal. calcd. for C_19_H_24_N_2_O_3_: C 69.49; H 7.37; N 8.53%. Found: C 69.79; H 7.54; N 8.76%.

*Ethyl 1-amino-5-ethyl-6,7,8,9-tetrahydrofuro[2,3-c]isoquinoline-2-carboxylate* (**4f**). Colorless solid, yield 83%, mp 140−142 °C; IR *ν*/cm^−1^: 3491, 3386 (NH_2_), 1682 (C=O). ^1^H-NMR δ: 1.27 (t, *J =* 7.4 Hz, 3H, CH_2_CH_3_), 1.40 (t, *J =* 7.1 Hz, 3H, OCH_2_CH_3_), 1.82–1.89 (m, 4H, 7,8-CH_2_), 2.67–2.72 (m, 2H, 6-CH_2_), 2.75 (q, *J =* 7.4 Hz, 2H, CH_2_CH_3_), 3.15–3.21 (m, 2H, 9-CH_2_), 4.32 (q, *J =* 7.1 Hz, 2H, OCH_2_CH_3_), 5.66 (br s, 2H, NH_2_). ^13^C-NMR δ: 11.54, 14.15, 20.98, 21.99, 24.87, 25.77, 27.29, 58.76, 109.30, 121.60, 124.12, 139.69, 142.42, 157.39, 160.55, 160.69. Anal. calcd. for C_16_H_20_N_2_O_3_: C 66.65; H 6.99; N 9.72%. Found: C 66.98; H 7.19; N 9.96%.

*Ethyl 1-amino-8,8-dimethyl-5-phenyl-8,9-dihydro-6H-furo[2,3-b]pyrano[4,3-d]pyridine-2-carboxylate* (**4n**). Colorless solid, yield 77%, mp 191−193 °C; IR *ν*/cm^−1^: 3453, 3336 (NH_2_), 1669 (C=O). ^1^H-NMR δ:1.34 (s, 6H, С(CH_3_)_2_), 1.42 (t, *J =* 7.1 Hz, 3H, CH_2_CH_3_), 3.21 (s, 2H, CH_2_), 4.36 (q, *J =* 7.1 Hz, 2H, CH_2_CH_3_), 4.67 (s, 2H, OCH_2_), 5.85 (s, 2H, NH_2_), 7.38−7.50 (m, 5H, Ph). ^13^C-NMR δ: 14.13, 26.24, 36.09, 59.01, 60.46, 68.81, 110.68, 121.94, 122.77, 127.57, 127.95, 128.20, 138.48, 139.21, 140.77, 154.47, 157.76, 160.46. Anal. calcd. for C_21_H_22_N_2_O_4_: C 68.84; H 6.05; N 7.65%. Found: C 69.20; H 6.27; N 7.91%.

#### 3.1.5. General Method for the Preparation of Compounds **5a**–**d**, **6a**–**n** and **7a**–**h**

A mixture of furo[2,3-*b*]pyridine **4** (10 mmol), the corresponding lactam (20 mmol) [2-pyrrolidinone (1.7 g); 2-piperidone (1.98 g), or 2-azepanone (2.26 g)] and phosphorus oxychloride (1.87 mL, 20 mmol) in absolute 1,2-dichloroethane (50 mL) was refluxed for 25 h. The solvent was distilled off to dryness and water (50 mL) was added to the residue. The separated crystals were filtered off, washed with water, dried, and recrystallized from ethanol.

*5-Methyl-1,2,3,4,11,12-hexahydropyrrolo[1″,2″:1′,2′]pyrimido[4′,5′:4,5]furo[2,3-c]isoquinolin-8(10H)-one* (**5a**). Colorless solid, yield 68%, mp 289−291 °C; IR *ν*/cm^−1^: 1686 (C=O). ^1^H-NMR δ: 1.83–1.99 (m, 4H, 2,3-CH_2_), 2.29–2.40 (m, 2H, 11-CH_2_), 2.52 (s, 3H, CH_3_), 2.71–2.76 (m, 2H, 4-CH_2_), 3.17 (t, *J =* 7.9 Hz, 2H, 12-CH_2_), 3.27–3.33 (m, 2H, 1-CH_2_), 4.17–4.22 (m, 2H, NCH_2_). ^13^C-NMR δ: 19.58, 20.79, 22.19, 25.42, 26.23, 31.72, 46.44, 110.07, 126.56, 134.93, 143.42, 143.46, 150.78, 157.01, 159.84, 160. 33. Anal. calcd. for C_17_H_17_N_3_O_2_: C 69.14; H 5.80; N 14.23%. Found: C 69.48; H 5.98; N 14.46%.

*5-Ethyl-1,2,3,4,11,12-hexahydropyrrolo[1″,2″:1′,2′]pyrimido[4′,5′:4,5]furo[2,3-c]isoquinolin-8(10H)-one* (**5b**). Cream solid, yield 70%; mp 208−210 °C; IR *ν*/cm^−^^1^: 1693 (C=O). ^1^H-NMR δ: 1.33 (t, *J =* 7.4 Hz, 3H, CH_2_CH_3_), 1.83–1.99 (m, 4H, 2,3-CH_2_), 2.29–2.40 (m, 2H, 11-CH_2_), 2.83 (q, *J =* 7.4 Hz, 2H, CH_2_CH_3_), 2.75–2.81 (m, 2H, 4-CH_2_), 3.18 (q, *J =* 7.9 Hz, 2H, 12-CH_2_), 3.29–3.35 (m, 2H, 1-CH_2_), 4.17–4.23 (m, 2H, NCH_2_). ^13^C-NMR δ: 11.55, 19.54, 20.77, 22.21, 24.79, 26.29, 27.37, 31.70, 46.42, 109.82, 125.90, 134.92, 143.40, 143.50, 150.73, 160.09, 160.26, 161.11. Anal. calcd. for C_18_H_19_N_3_O_2_: C 69.88; H 6.19; N 13.58%. Found: C 70.21; H 6.38; N 13.82%.

5-Isopropyl-2,2-dimethyl-1,4,11,12-tetrahydro-2H-pyrano[4″,3″:4′,5′]pyrido[3′,2′:4,5]furo[3,2-d]pyrrolo[1,2-a]pyrimidin-8(10H)-one (**5c**). Colorless solid, yield 65%; mp 244‒246 °C; IR ν/cm^−1^: 1701 (C=O). ^1^H-NMR δ: 1.31 (d, J = 6.6 Hz, 6H, CH(CH_3_)_2_), 1.34 (s, 6H, C(CH_3_)_2_), 2.31−2.41 (m, 2H, 11-CH_2_), 3.05 (sept., J = 6.6 Hz, 1H, CH(CH_3_)_2_), 3.21 (t, J = 8.1 Hz, 2H, 12-CH_2_), 3.23 (s, 2H, 1-CH_2_), 4.19− 4.25 (m, 2H, NCH_2_), 4.86 (s, 2H, OCH_2_). ^13^C-NMR δ: 19.58, 21.22, 25.83, 30.38, 31.70, 36.61, 38.95, 46.50, 59.66, 68.50, 110.19, 122.18, 135.31, 140.26, 143.08, 150.71, 160.67, 160.82, 161.95. Anal. calcd. for C_20_H_23_N_3_O_3_: C 67.97; H 6.56; N 11.89%. Found: C 68.32; H 6.77; N 12.15%.

2,2-Dimethyl-5-phenyl-1,4,11,12-tetrahydro-2H-pyrano[4″,3″:4′,5′]pyrido[3′,2′:4,5]furo[3,2-d]pyrrolo[1,2-a]pyrimidin-8(10H)-one (**5d**). Colorless solid, yield 69%; mp 292−293 °C; IR ν/cm^−^^1^: 1687 (C=O). ^1^H-NMR δ: 1.38 (s, 6H, С(CH_3_)_2_), 2.31–2.43 (m, 2H, 11-CH_2_), 3.22 (t, J = 7.9 Hz, 2H, 12-CH_2_), 3.30 (s, 2H, CH_2_), 4.19− 4.25 (m, 2H, NCH_2_), 4.76 (s, 2H, OCH_2_), 7.41−7.57 (m, 5H, Ph). ^13^C-NMR δ: 19.59, 26.20, 31.75, 36.73, 46.57, 60.50, 68.81, 111.20, 124.01, 127.66, 128.20, 128.33, 136.04, 138.07, 141.39, 142.88, 150.69, 154.79, 160.46, 160.98. Anal. calcd. for C_23_H_21_N_3_O_3_: C 71.30; H 5.46; N 10.85%. Found: C 71.67; H 5.69; N 11.12%.

*4-Isopropyl-2,3,9,10,11,12-hexahydrocyclopenta[4′,5′]pyrido[3′,2′:4,5]furo[3,2-d]pyrido[1,2-a]pyrimidin-7(1H)-one* (**6a**). Cream solid, yield 57%; mp 147‒148 °C; IR *ν*/cm^−1^: 1691 (C=O). ^1^H-NMR δ: 0.99 (d, *J* = 6.8 Hz, 6H, CH(CH_3_)_2_), 1.90−2.08 (m, 4H, 10,11-CH_2_), 2.22−2.33 (m, 2H, 2-CH_2_), 2.96−3.05 (m, 4H, 3,12-CH_2_), 3.19 (sept., *J* = 6.8 Hz, 1H, CH(CH_3_)_2_), 3.33 (t, *J* = 7.6 Hz, 2H, 1-CH_2_), 4.08 (t, *J* = 6.1 Hz, 2H, NCH_2_). ^13^C-NMR δ: 18.40, 20.96, 21.15, 24.43, 29.03, 31.08, 31.11, 32.83, 41.51, 109.04, 133.05, 134.96, 140.58, 149.65, 151.81, 156.19, 161.45, 161.70. Anal. calcd. for C_19_H_21_N_3_O_2_: C 70.57; H 6.55; N 12.99%. Found: C 70.91; H 6.75; N 13.24%.

*4-Butyl-2,3,9,10,11,12-hexahydrocyclopenta[4′,5′]pyrido[3′,2′:4,5]furo[3,2-d]pyrido[1,2-a]pyrimidin-7(1H)-one* (**6b**). Cream solid, yield 53%; mp 142‒144 °C; IR *ν*/cm^−1^: 1698 (C=O). ^1^H-NMR δ: 0.99 (t, *J* = 7.3 Hz, 3H, CH_2_CH_3_), 1.37−1.50 (m, 2H, CH_2_CH_3_), 1.71−1.81 (m, 2H, CH_2_C_2_H_5_), 1.91−2.08 (m, 4H, 10,11-CH_2_), 2.23−2.33 (m, 2H, 2-CH_2_), 2.78−2.83 (m, 2H, CH_2_C_3_H_7_), 2.96−3.02 (m, 4H, 3,12-CH_2_), 3.35 (t, *J* = 7.6 Hz, 2H, 1-CH_2_), 4.09 (t, *J* = 6.1 Hz, 2H, NCH_2_). ^13^C-NMR δ: 11.52, 18.40, 21.16, 21.90, 24.40, 29.25, 29.76, 31.10, 31.13, 34.94, 41.50, 109.02, 134.03, 134.94, 140.63, 149.44, 151.86, 156.23, 157.01, 161.55. Anal. calcd. for C_20_H_23_N_3_O_2_: C 71.19; H 6.87; N 12.45%. Found: C 71.51; H 7.06; N 12.68%.

*4-Cyclohexyl-2,3,9,10,11,12-hexahydrocyclopenta[4′,5′]pyrido[3′,2′:4,5]furo[3,2-d]pyrido[1,2-a]pyrimidin-7(1H)-one* (**6c**). Light yellow solid, yield 56%; mp 186‒188 °C. ^1^H-NMR δ: 1.31−1.50 and 1.64−2.08 (both m, 3H and 11H, 5CH_2_-C_6_H_11_, 10,11-CH_2_), 2.22−2.33 (m, 2H, 2-CH_2_), 2.74−2.87 (m, 1H, CH-C_6_H_11_), 2.99 (t, *J* = 7.6 Hz, 2H, 3-CH_2_), 3.01 (t, *J* = 6.6 Hz, 2H, 13-CH_2_), 3.34 (t, *J* = 7.6 Hz, 2H, 1-CH_2_), 4.09 (t, *J* = 6.1 Hz, 2H, NCH_2_). ^13^C-NMR δ: 18.40, 21.15, 24.42, 25.44, 25.90, 29.06, 30.84, 31.08, 31.14, 41.50, 43.16, 108.94, 133.21, 134.93, 140.62, 149.54, 151.81, 156.15, 160.73, 161.68. Anal. calcd. for C_22_H_25_N_3_O_2_: C 72.70; H 6.93; N 11.56%. Found: C 73.06; H 7.15; N 11.82%.

*5-Methyl-1,2,3,4,10,11,12,13-octahydro-8H-pyrido[1″,2″:1′,2′]pyrimido[4′,5′:4,5]furo[2,3-c]isoquinolin-8-one* (**6d**). Colorless solid, yield 61%; mp 282−284 °C; IR *ν*/cm^−^^1^: 1689 (C=O). ^1^H-NMR δ: 1.83–2.07 (m, 8H, 2,3,11,12-CH_2_), 2.52 (s, 3H, CH_3_), 2.71–2.77 (m, 2H, 4-CH_2_), 2.99 (t, *J =* 6.6 Hz, 2H, 13-CH_2_), 3.28–3.34 (m, 2H, 1-CH_2_), 4.08 (t, *J =* 6.1 Hz, 2H, NCH_2_). ^13^C-NMR δ: 18.42, 20.81, 21.16, 22.19, 22.22, 25.39, 26.20, 31.18, 41.43, 109.93, 126.49, 134.43, 141.52, 143.52, 151.83, 155.90, 157.07, 159.81. Anal. calcd. for C_18_H_19_N_3_O_2_: C 69.88; H 6.19; N 13.58%. Found: C 70.20; H 6.37; N 13.82%.

*5-Ethyl-1,2,3,4,10,11,12,13-octahydro-8H-pyrido[1″,2″:1′,2′]pyrimido[4′,5′:4,5]furo[2,3-c]isoquinolin-8-one* (**6e**). Colorless solid, yield 54%; mp 194‒196 °C; IR *ν*/cm^−1^: 1697 (C=O). ^1^H-NMR δ: 1.33 (t, *J =* 7.4 Hz, 3H, CH_2_CH_3_), 1.83–2.08 (m, 8H, 2,3,11,12-CH_2_), 2.76–2.82 (m, 2H, 4-CH_2_), 2.83 (q, *J =* 7.4 Hz, 2H, CH_2_CH_3_), 3.00 (t, *J =* 6.6 Hz, 2H, 13-CH_2_), 3.31–3.36 (m, 2H, 1-CH_2_), 4.09 (t, *J =* 6.1 Hz, 2H, NCH_2_). ^13^C-NMR δ: 11.55, 18.44, 20.81, 21.16, 22.27, 24.79, 26.29, 27.38, 31.18, 41.44, 109.72, 125.87, 134.47, 141.52, 143.63, 151.81, 155.85, 160.09, 161.21. Anal. calcd. for C_19_H_21_N_3_O_2_: C 70.57; H 6.55; N 12.99%. Found: C 70.91; H 6.74; N 13.24%.

*5-Isopropyl-1,2,3,4,10,11,12,13-octahydro-8H-pyrido[1″,2″:1′,2′]pyrimido[4′,5′:4,5]furo[2,3-c]isoquinolin-8-one* (**6f**). Light yellow solid, yield 58%; mp 169‒171 °C; IR *ν*/cm^−1^: 1695 (C=O). ^1^H-NMR δ: 1.29 (d, *J =* 6.6 Hz, 6H, CH(CH_3_)_2_), 1.84–2.08 (m, 8H, 2,3,11,12-CH_2_), 2.83–2.88 (m, 2H, 4-CH_2_), 3.00 (t, *J =* 6.6 Hz, 2H, 13-CH_2_), 3.33 (sept., *J =* 6.6 Hz, 1H, CH(CH_3_)_2_), 3.33–3.38 (m, 2H, 1-CH_2_), 4.09 (t, *J =* 6.1 Hz, 2H, NCH_2_). ^13^C-NMR δ: 18.44, 20.76, 21.17, 21.29, 22.42, 24.67, 26.48, 30.42, 31.18, 41.44, 109.79, 124.97, 134.56, 141.52, 144.05, 151.81, 155.82, 160.25, 164.99. Anal. calcd. for C_20_H_23_N_3_O_2_: C 71.19; H 6.87; N 12.45%. Found: C 71.50; H 7.05; N 12.68%.

*5-Isobutyl-1,2,3,4,10,11,12,13-octahydro-8H-pyrido[1″,2″:1′,2′]pyrimido[4′,5′:4,5]furo[2,3-c]isoquinolin-8-one* (**6g**). Colorless solid, yield 55%; mp 208‒209 °C; IR *ν*/cm^−1^: 1687 (C=O). ^1^H-NMR δ: 1.00 (d, *J =* 6.6 Hz, 6H, CH(CH_3_)_2_), 1.83–2.07 (m, 8H, 2,3,11,12-CH_2_), 2.21−2.35 (m, 1H, CH(CH_3_)_2_), 2.68 (d, *J =* 7.1 Hz, 2H, CHCH_2_), 2.76–2.82 (m, 2H, 4-CH_2_), 3.00 (t, *J =* 6.6 Hz, 2H, 13-CH_2_), 3.31–3.37 (m, 2H, 1-CH_2_), 4.09 (t, *J =* 6.1 Hz, 2H, NCH_2_). ^13^C-NMR δ: 18.44, 20.81, 21.18, 22.32, 22.36, 25.23, 26.36, 27.07, 31.18, 41.42, 42.98, 109.77, 126.41, 134.55, 140.98, 141.54, 143.76, 151.82, 155.85, 159.80. Anal. calcd. for C_21_H_25_N_3_O_2_: C 71.77; H 7.17; N 11.96%. Found: C 72.12; H 7.38; N 12.22%.

*2,2,5-Trimethyl-1,4,10,11,12,13-hexahydro-2H,8H-pyrano[4″,3″:4′,5′]pyrido[3′,2′:4,5]furo[3,2-d]pyrido[1,2-a]pyrimidin-8-one* (**6h**). Colorless solid, yield 60%; mp 278‒280 °C; IR *ν*/cm^−1^: 1698 (C=O). ^1^H-NMR δ: 1.33 (s, 6H, С(CH_3_)_2_), 1.91–2.08 (m, 4H, 11,12-CH_2_), 2.48 (s, 3H, CH_3_), 3.02 (t, *J =* 6.6 Hz, 2H, 13-CH_2_), 3.20 (s, 2H, CH_2_), 4.10 (t, *J =* 6.1 Hz, 2H, NCH_2_), 4.74 (s, 2H, OCH_2_). ^13^C-NMR δ: 18.32, 20.91, 21.07, 25.84, 31.15, 36.28, 41.47, 60.02, 68.68, 110.01, 123.80, 134.58, 139.73, 141.16, 151.86, 153.81, 156.63, 160.30. Anal. calcd. for C_19_H_21_N_3_O_3_: C 67.24; H 6.24; N 12.38%. Found: C 67.57; H 6.43; N 12.62%.

5-Ethyl-2,2-dimethyl-1,4,10,11,12,13-hexahydro-2H,8H-pyrano[4″,3″:4′,5′]pyrido[3′,2′:4,5] furo[3,2-d]pyrido[1,2-a]pyrimidin-8-one (**6i**). Colorless solid, yield 62%; mp 232‒234 °C; IR ν/cm^−1^: 1692 (C=O). ^1^H-NMR δ: 1.33 (s, 6H, С(CH_3_)_2_), 1.35 (t, J = 7.4 Hz, 3H, CH_2_CH_3_), 1.91–2.08 (m, 4H, 11,12-CH_2_), 2.74 (q, J = 7.4 Hz, 2H, CH_2_CH_3_), 3.02 (t, J = 6.6 Hz, 2H, 13-CH_2_), 3.22 (s, 2H, CH_2_), 4.10 (t, J = 6.1 Hz, 2H, NCH_2_), 4.79 (s, 2H, OCH_2_). ^13^C-NMR δ: 11.29, 18.34, 21.11, 25.81, 26.42, 31.14, 36.40, 41.39, 59.69, 68.56, 109.90, 123.05, 134.71, 139.83, 141.13, 151.76, 156.32, 158.01, 160.64. Anal. calcd. for C_20_H_23_N_3_O_3_: C 67.97; H 6.56; N 11.89%. Found: C 68.34; H 6.78; N 20.95%.

5-Isopropyl-2,2-dimethyl-1,4,10,11,12,13-hexahydro-2H,8H-pyrano[4″,3″:4′,5′]pyrido[3′,2′:4,5] furo[3,2-d]pyrido[1,2-a]pyrimidin-8-one (**6j**). Colorless solid, yield 57%; mp 239‒241 °C; IR ν/cm^−1^: 1697 (C=O). ^1^H-NMR δ: 1.31 (d, J = 6.7 Hz, 6H, CH(CH_3_)_2_), 1.34 (s, 6H, С(CH_3_)_2_), 1.91–2.08 (m, 4H, 11,12-CH_2_), 3.02 (t, J = 6.6 Hz, 2H, 13-CH_2_), 3.05 (sept., J = 6.7 Hz, 1H, CH(CH_3_)_2_), 3.24 (s, 2H, CH_2_), 4.11 (t, J = 6.1 Hz, 2H, NCH_2_), 4.86 (s, 2H, OCH_2_). ^13^C-NMR δ: 18.34, 21.11, 21.22, 25.82, 30.38, 31.13, 36.58, 41.40, 59.66, 68.49, 110.06, 122.12, 134.81, 140.35, 141.16, 151.76, 156.32, 160.78, 162.01. Anal. calcd. for C_21_H_25_N_3_O_3_: C 68.64; H 6.86; N 11.44%. Found: C 68.99; H 7.05; N 11.69%.

5-Butyl-2,2-dimethyl-1,4,10,11,12,13-hexahydro-2H,8H-pyrano[4″,3″:4′,5′]pyrido[3′,2′:4,5] furo[3,2-d]pyrido[1,2-a]pyrimidin-8-one (**6k**). Colorless solid, yield 61%; mp 163‒164 °C; IR ν/cm^−1^: 1696 (C=O). ^1^H-NMR δ: 1.00 (t, J = 7.3 Hz, 3H, CH_2_CH_3_), 1.33 (s, 6H, С(CH_3_)_2_), 1.39−1.54 (m, 2H, CH_2_CH_3_), 1.72−1.82 (m, 2H, CH_2_C_2_H_5_), 1.91–2.08 (m, 4H, 11,12-CH_2_), 2.69 (t, J = 7.6 Hz, 2H, CH_2_C_3_H_7_), 3.02 (t, J = 6.6 Hz, 2H, 13-CH_2_), 3.21 (s, 2H, CH_2_), 4.10 (t, J = 6.1 Hz, 2H, NCH_2_), 4.79 (s, 2H, OCH_2_). ^13^C-NMR δ: 13.53, 18.34, 21.11, 21.94, 25.81, 29.38, 31.13, 33.01, 36.43, 41.38, 59.79, 68.56, 109.94, 123.19, 134.74, 139.96, 141.16, 151.76, 156.33, 157.33, 160.56. Anal. calcd. for C_22_H_27_N_3_O_3_: C 69.27; H 7.13; N 11.02%. Found: C 69.58; H 7.30; N 11.25%.

5-Isobutyl-2,2-dimethyl-1,4,10,11,12,13-hexahydro-2H,8H-pyrano[4″,3″:4′,5′]pyrido[3′,2′:4,5] furo[3,2-d]pyrido[1,2-a]pyrimidin-8-one (**6l**). Colorless solid, yield 53%; mp 212‒215 °C; IR ν/cm^−1^: 1699 (C=O). ^1^H-NMR δ: 1.00 (d, J = 6.6 Hz, 6H, CH(CH_3_)_2_), 1.33 (s, 6H, С(CH_3_)_2_), 1.91–2.08 (m, 4H, 11,12-CH_2_), 2.23–2.37 (m, 1H, CHCH_2_), 2.57 (d, J = 7.0 Hz, 2H, CHCH_2_), 3.02 (t, J = 6.6 Hz, 2H, 13-CH_2_), 3.23 (s, 2H, CH_2_), 4.10 (t, J = 6.0 Hz, 2H, NCH_2_), 4.79 (s, 2H, OCH_2_). ^13^C-NMR δ: 18.33, 21.11, 22.16, 25.79, 27.31, 31.12, 36.45, 41.41, 42.06, 59.98, 68.52, 109.97, 123.62, 134.78, 140.04, 141.16, 151.77, 156.37, 156.72, 160.48. Anal. calcd. for C_22_H_27_N_3_O_3_: C 69.27; H 7.13; N 11.02%. Found: C 69.60; H 7.33; N 11.26%.

2,2-Dimethyl-5-phenyl-1,4,10,11,12,13-hexahydro-2H,8H-pyrano[4″,3″:4′,5′]pyrido[3′,2′:4,5] furo [3,2-d]pyrido[1,2-a]pyrimidin-8-one (**6m**). Colorless solid, yield 59%; mp 245‒247 °C; IR ν/cm^−1^: 1684 (C=O). ^1^H-NMR δ: 1.38 (s, 6H, С(CH_3_)_2_), 1.93–2.10 (m, 4H, 11,12-CH_2_), 3.05 (t, J = 6.6 Hz, 2H, 13-CH_2_), 3.32 (s, 2H, CH_2_), 4.12 (t, J = 6.1 Hz, 2H, NCH_2_), 4.76 (s, 2H, OCH_2_), 7.41−7.58 (m, 5H, Ph). ^13^C-NMR δ: 18.32, 21.09, 26.20, 31.18, 36.70, 41.51, 60.51, 68.80, 111.09, 123.96, 127.65, 128.20, 128.33, 135.51, 138.09, 140.97, 141.48, 151.76, 154.86, 156.63, 160.44. Anal. calcd. for C_24_H_23_N_3_O_3_: C 71.80; H 5.77; N 10.47%. Found: C 72.16; H 5.99; N 10.72%.

5-(2-Furyl)-2,2-dimethyl-1,4,10,11,12,13-hexahydro-2H,8H-pyrano[4″,3″:4′,5′]pyrido[3′,2′:4,5] furo[3,2-d]pyrido[1,2-a]pyrimidin-8-one (**6n**). Cream solid, yield 56%; mp 290‒292 °C; IR ν/cm^−1^: 1683 (C=O). ^1^H-NMR δ: 1.37 (s, 6H, С(CH_3_)_2_), 1.92–2.08 (m, 4H, 11,12-CH_2_), 3.03 (t, J = 6.6 Hz, 2H, 13-CH_2_), 3.29 (s, 2H, CH_2_), 4.11 (t, J = 6.1 Hz, 2H, NCH_2_), 5.16 (s, 2H, OCH_2_), 6.63 (dd, J = 3.5, 1.7 Hz, 1H, 4-CH_fur._), 7.24 (dd, J = 3.5, 0.8 Hz, 1H, 3-CH_fur._), 7.74 (dd, J = 1.7, 0.8 Hz, 1H, 5-CH_fur._). ^13^C-NMR δ: 18.25, 21.03, 25.90, 31.18, 36.94, 38.67, 41.55, 60.65, 68.18, 110.94, 111.79, 112.76, 122.43, 135.55, 141.04, 141.94, 143.10, 144.33, 151.79, 152.86, 156.96, 160.04. Anal. calcd. for C_22_H_21_N_3_O_4_: C 67.51; H 5.41; N 10.74%. Found: C 67.85; H 5.59; N 10.97%.

*4-Cyclohexyl-2,3,10,11,12,13-hexahydro-1H-cyclopenta[4″,5″]pyrido[3″,2″:4′,5′]furo[3′,2′:4,5] pyrimido[1,2-a]azepin-7(9H)-one* (**7a**). Colorless solid, yield 68%; mp 205‒206 °C; IR *ν*/cm^−1^: 1698 (C=O). ^1^H-NMR δ: 1.31−1.50 and 1.64−1.96 (both m, 3H and 13H, 5CH_2_-C_6_H_11_, 10,11,12-CH_2_), 2.22−2.34 (m, 2H, 2-CH_2_), 2.74−2.86 (m, 1H, CH-C_6_H_11_), 3.02 (t, *J* = 7.4 Hz, 2H, 3-CH_2_), 3.11−3.17 (m, 2H, 13-CH_2_), 3.36 (t, *J* = 7.6 Hz, 2H, 1-CH_2_), 4.40−4.46 (m, 2H, NCH_2_). ^13^C-NMR δ: 24.47, 25.43, 25.88, 27.07, 28.69, 29.07, 30.84, 31.14, 36.59, 41.89, 43.16, 109.15, 133.26, 135.03, 140.42, 149.56, 151.80, 160.76, 160.90, 161.79. Anal. calcd. for C_23_H_27_N_3_O_2_: C 73.18; H 7.21; N 11.13%. Found: C 73.51; H 7.40; N 11.37%.

*4-(2-Furyl)-2,3,10,11,12,13-hexahydro-1H-cyclopenta[4″,5″]pyrido[3″,2″:4′,5′]furo[3′,2′:4,5] pyrimido[1,2-a]azepin-7(9H)-one* (**7b**). Cream solid, yield 70%; mp 308‒310 °C; IR *ν*/cm^−1^: 1689 (C=O). ^1^H-NMR δ: 1.76–1.95 (m, 6H, 10,11,12-CH_2_), 2.28–2.39 (m, 2H, 2-CH_2_), 3.12–3.18 (m, 2H, 13-CH_2_), 3.02 (t, *J =* 7.4 Hz, 2H, 3-CH_2_), 3.36 (t, *J =* 7.6 Hz, 2H, 1-CH_2_), 4.41–4.46 (m, 2H, NCH_2_), 6.61 (dd, *J* = 3.5, 1.7 Hz, 1H, 4-CH_fur._), 7.19 (dd, *J* = 3.5, 0.7 Hz, 1H, 3-CH_fur._), 7.71 (dd, *J* = 1.7, 0.8 Hz, 1H, 5-CH_fur._). ^13^C-NMR δ: 24.39, 24.62, 26.95, 28.60, 30.80, 31.07, 36.59, 42.04, 110.02, 111.60, 111.85, 131.81, 135.75, 140.31, 142.74, 144.13, 151.88, 151.93, 152.93, 161.20, 161.66. Anal. calcd. for C_21_H_19_N_3_O_3_: C 69.79; H 5.30; N 11.63%. Found: C 70.14; H 5.51; N 11.89%.

*5-Methyl-1,2,3,4,11,12,13,14-octahydroazepino[1″,2″:1′,2′]pyrimido[4′,5′:4,5]furo[2,3-c] isoquinolin-8(10H)-one* (**7c**). Colorless solid, yield 66%; mp 259‒261 °C; IR *ν*/cm^−1^: 1698 (C=O). ^1^H-NMR δ: 1.75–2.00 (m, 10H, 2,3,11,12,13-CH_2_), 2.53 (m, 3H, CH_3_), 2.71–2.77 (m, 2H, 4-CH_2_), 3.11−3,17 (m, 2H, 14-CH_2_), 3.30–3.36 (m, 2H, 1-CH_2_), 4.39–4.45 (m, 2H, NCH_2_). ^13^C-NMR δ: 20.81, 22.20, 22.24, 24.49, 25.39, 26.21, 27.06, 28.73, 36.73, 41.87, 110.12, 126.55, 110.12, 141.30, 143.52, 151.81, 157.09, 159.90, 160.68. Anal. calcd. for C_19_H_21_N_3_O_2_: C 70.57; H 6.55; N 12.99%. Found: C 70.94; H 6.76; N 13.26%.

*5-Ethyl-1,2,3,4,11,12,13,14-octahydroazepino[1″,2″:1′,2′]pyrimido[4′,5′:4,5]furo[2,3-c] isoquinolin-8(10H)-one* (**7d**). Milky solid, yield 65%; mp 214‒215 °C; IR *ν*/cm^−1^: 1692 (C=O). ^1^H-NMR δ: 1.33 (t, *J =* 7.4 Hz, 3H, CH_2_CH_3_), 1.76–1.99 (m, 10H, 2,3,11,12,13-CH_2_), 2.76–2.82 (m, 2H, 4-CH_2_), 2.83 (q, *J =* 7.4 Hz, 2H, CH_2_CH_3_), 3.11−3,17 (m, 2H, 14-CH_2_), 3.31–3.38 (m, 2H, 1-CH_2_), 4.40–4.45 (m, 2H, NCH_2_). ^13^C-NMR δ: 11.56, 20.81, 22.29, 24.49, 24.79, 26.30, 27.07, 27.39, 28.73, 36.73, 41.85, 109.92, 125.90, 134.55, 141.29, 143.63, 151.78, 160.20, 160.60, 161.24. Anal. calcd. for C_20_H_23_N_3_O_2_: C 71.19; H 6.87; N 12.45%. Found: C 71.52; H 7.07; N 12.69%.

5-Isopropyl-2,2-dimethyl-1,4,11,12,13,14-hexahydro-2H-pyrano[4‴,3‴:4″,5″]pyrido[3″,2″:4′,5′] furo[3′,2′:4,5]pyrimido[1,2-a]azepin-8(10H)-one (**7e**). Colorless solid, yield 67%; mp 223‒225 °C; IR ν/cm^−1^: 1694 (C=O). ^1^H-NMR δ: 1.30 (d, J = 6.6 Hz, 6H, CH(CH_3_)_2_), 1.34 (s, 6H, C(CH_3_)_2_), 1.75−1.94 (m, 6H, 11,12,13-CH_2_), 3.04 (sept., J = 6.6 Hz, 1H, CH(CH_3_)_2_), 3.13−3.21 (m, 2H, 14-CH_2_), 3.24 (s, 2H, CH_2_), 4.40−4.47 (m, 2H, NCH_2_), 4.85 (s, 2H, OCH_2_). ^13^C-NMR δ: 21.20, 21.24, 24.53, 25.81, 27.08, 28.69, 30.38, 30.42, 36.61, 36.59, 41.93, 59.66, 68.49, 110.27, 122.15, 134.87, 140.35, 140.92, 151.76, 161.01, 162.04. Anal. calcd. for C_22_H_27_N_3_O_3_: C 69.27; H 7.13; N 11.02%. Found: C 69.58; H 7.32; N 11.25%.

5-Isobutyl-2,2-dimethyl-1,4,11,12,13,14-hexahydro-2H-pyrano[4‴,3‴:4″,5″]pyrido[3″,2″:4′,5′] furo[3′,2′:4,5]pyrimido[1,2-a]azepin-8(10H)-one (**7f**). Colorless solid, yield 64%; mp 197‒199 °C; IR ν/cm^−1^: 1701 (C=O). ^1^H-NMR δ: 0.99 (d, J = 6.6 Hz, 6H, CH(CH_3_)_2_), 1.32 (s, 6H, C(CH_3_)_2_), 1.74−1.95 (m, 6H, 11,12,13-CH_2_), 2.21–2.37 (m, 1H, CHCH_2_), 2.57 (d, J = 7.0 Hz, 2H, CHCH_2_), 3.12−3.21 (m, 2H, 14-CH_2_), 3.22 (s, 2H, CH_2_), 4.39−4.48 (m, 2H, NCH_2_), 4.79 (s, 2H, OCH_2_). ^13^C-NMR δ: 22.12, 24.53, 25.78, 27.10, 27.33, 28.70, 36.48, 36.60, 41.94, 42.05, 60.00, 68.54, 110.19, 123.68, 134.87, 140.05, 140.93, 151.78, 156.73, 160.57, 161.07. Anal. calcd. for C_23_H_29_N_3_O_3_: C 69.85; H 7.39; N 10.62%. Found: C 70.21; H 7.61; N 10.88%.

2,2-Dimethyl-5-phenyl-1,4,11,12,13,14-hexahydro-2H-pyrano[4‴,3‴:4″,5″]pyrido[3″,2″:4′,5′] furo[3′,2′:4,5]pyrimido[1,2-a]azepin-8(10H)-one (**7g**). Colorless solid, yield 71%; mp 228‒229 °C; IR ν/cm^−1^: 1702 (C=O). ^1^H-NMR δ: 1.39 (s, 6H, C(CH_3_)_2_), 1.78−1.96 (m, 6H, 11,12,13-CH_2_), 3.17−3.23 (m, 2H, 14-CH_2_), 3.33 (s, 2H, CH_2_), 4.43−4.49 (m, 2H, NCH_2_), 4.77 (s, 2H, OCH_2_), 7.41−7.56 (m, 5H, Ph). ^13^C-NMR δ: 24.49, 26.18, 27.02, 28.69, 36.65, 36.71, 42.04, 60.51, 68.78, 111.28, 123.97, 127.64, 128.20, 128.31, 135.59, 138.06, 140.73, 141.47, 151.75, 154.89, 160.52, 161.32. Anal. calcd. for C_25_H_25_N_3_O_3_: C 72.27; H 6.06; N 10.11%. Found: C 72.59; H 6.26; N 10.35%.

5-(2-Furyl)-2,2-dimethyl-1,4,11,12,13,14-hexahydro-2H-pyrano[4‴,3‴:4″,5″]pyrido[3″,2″:4′,5′] furo [3′,2′:4,5]pyrimido[1,2-a]azepin-8(10H)-one (**7h**). Cream solid, yield 69%; mp 299‒300 °C; IR ν/cm^−1^: 1693 (C=O). ^1^H-NMR δ: 1.38 (s, 6H, С(CH_3_)_2_), 1.77–1.95 (m, 6H, 11,12,13-CH_2_), 3.15−3.21 (m, 2H, 14-CH_2_), 3.30 (s, 2H, CH_2_), 4.42−4.47 (m, 2H, NCH_2_), 5.16 (s, 2H, OCH_2_), 6.63 (dd, J = 3.5, 1.7 Hz, 1H, 4-CH_fur._), 7.24 (dd, J = 3.5, 0.8 Hz, 1H, 3-CH_fur._), 7.74 (dd, J = 1.7, 0.8 Hz, 1H, 5-CH_fur._). ^13^C-NMR δ: 24.47, 25.85, 27.03, 28.72, 36.64, 36.94, 42.00, 60.65, 68.04, 111.09, 111.64, 112.63, 122.39, 135.65, 140.73, 141.81, 143.08, 143.86, 151.64, 153.14, 160.11, 161.28. Anal. calcd. for C_23_H_23_N_3_O_4_: C 68.13; H 5.72; N 10.36%. Found: C 68.47; H 5.91; N 10.61%.

### 3.2. Biological Evaluation

Compounds were studied for their possible neurotropic activities (anticonvulsant, sedative, anti-anxiety activity) as well as side effects on 450 white mice of both sexes weighing 18–24 g and 50 male rats of the Wistar line weighing 120–140 g. All groups of animals were maintained at 25 ± 2 ºC in the same room, on a common food ration. As reference compounds the known antiepileptic drug ethosuximide and tranquilizer diazepam were used. All the biological experiments were carried out in full compliance with the European Convention for the Protection of Vertebrate. All animal procedures were performed in accordance with the Guidelines for Care and Use of Laboratory Animals of “(ETS No 123, Strasbourg, 03/18/1986): Strasbourg (France).

#### 3.2.1. Evaluation of the Anticonvulsant Activity of the Synthesized Compounds

The anticonvulsant effect of the new synthesized compounds was investigated by tests: pentylenetetrazole, thiosemicarbazide convulsions, maximal electroshock (MES).

The pentylenetetrazole (PTZ) test is an experimental model for inducing myoclonic seizures, as well as for predicting the anxiolytic properties of compounds. Pentylenetetrazole (PTZ) is a common convulsant agent used in animal models to investigate the mechanisms of seizures. Out bred mice (weight 18–22 g) were used for the study. In the case of convulsions induced by PTZ, the PTZ was injected subcutaneously at 90 mg/kg, which induced convulsions in 95% of animals (CD_95%_). Each animal is placed into an individual plastic cage for observation lasting 1 h. Seizure and clonic convulsions are recorded. Substances were administered intraperitoneally (i.p.) at doses of 10, 25, 50, 75, 100, 150, 200 mg/kg in suspension with carboxymethylcellulose with Twin-80 45 min before administration of PTZ and applying electrical stimulation. The control animals were administered an emulsifier. Every dose of each test compound was studied in six animals.

MES test is used as an animal model for the generalized tonic seizures of epilepsy. The parameters of MES were: 50 mA, duration 0.2 s, the oscillation frequency 50 imp/s. Anticonvulsant properties of compounds was assessed by the prevention of tonic-extensor phase of convulsions.

Thiosemicarbazide being antimetabolite of GABA inhibitor (glutamic acid decarboxylase) in the brain is administered subcutaneously to mice at a dose of 18 mg/kg as a 0.5% solution causes clonic convulsions in animals. Antithiosemicarbazide activity was evaluated on latency time of the onset of seizures. Substances were administered intraperitoneally (i.p.) at doses of 100 mg/kg in suspension with carboxymethylcellulose with Twin-80 45 min before administration of thiosemicarbazide.

The comparative drug ethosuximide was administered in 200 mg/kg and diazepam in 2 mg/kg doses.

#### 3.2.2. Evaluation of the Psychotropic Properties of the Synthesized Compounds

Psychotropic properties of selected compounds were studied by tests: “open field”, “elevated plus maze” (EPM), “forced swimming”.

*Open field test.* The research-motor behavior of rats was studied on a modified “open field” model [30,31,32]. For this purpose, an installation was used, the bottom of which is divided into squares with holes (cells). Experiments were performed in the daytime with natural light. Within 5 min of the experiment, the indicators of sedative and activating behavior were determined, namely, the number of horizontal movements, standing on the hind legs (vertical movements), sniffing of the cells. The number of animals on this model was 8 for each compound, control, and reference drug. The studied compounds were administered to rats in the most effective dose of 50 mg/kg intraperitoneally as a suspension with methylcarboxycellulose with Tween-80.

*Elevated plus maze—EPM test.* Anti-anxiety and sedative effects were studied on a model of the “elevated plus maze” in mice. The labyrinth is a cruciform machine raised above the floor, having a pair of open and closed sleeves opposed to each other. Normal animals prefer to spend most of their time in the closed (dark) sleeves of the labyrinth. The anxiolytic effect of the compounds was estimated by the increase in the number of entries into open (light) sleeves and the time spent in, without increasing the total motor activity. This records the time spent in the closed sleeve, the number of attempts to enter the installation center. In the above model, the test compounds and the reference drug were injected intraperitoneally before the experiments. The control animals were administered an emulsifier. Results were processed statistically (*p* ≤ 0.05).

*Forced swimming test*. To assess “despair and depression” the model “compelling swimming” was used. Experimental animals were forced to swim in a glass container (height 22 cm, diameter 14 cm), filled 1/3 with water. Intact mices swim very actively, but soon they will be forced to immobilize. The latent period of immobilization, the total duration of active swimming, immobilization is fixed for 6 min (the experiments were conducted under natural light).

#### 3.2.3. Evaluation of Incoordination of Movements in the Rotating Rod Test

Adverse neurotoxic (muscle relaxant) effect of compounds was studied in doses of 50 to 500 mg/kg when administered intraperitoneally, as well as reference drugs in effective anticonvulsant doses. Miorelaxation was investigated by the test of a ″rotating rod″ in mice. To this end, mice were planted on a metal rod with a corrugated rubber coating, which rotated at a speed of 5 revolutions per minute. The number of animals that cannot stay on it for 2 min was determined. To determine the ED_50_, neurotoxic TD_50_ and LD_50_ the statistical method of penetration by Litchfield and Wilcoxon was used. The acute toxicity (LD_50_), was determined by calculating the number of dead animals after 24 h of exposure (i.p. injection) in doses 100–2200 mg/kg.

### 3.3. Docking Studies

Docking studies were performed using AutoDock 4.2 into the 3D structures of GABA_A_ receptor (PDB code: 4COF), SERT transporter (PDB code: 3F3A) and 5-HT_1A_ receptor (PDB code: 3NYA), retrieved from Protein Data Bank (PDB). For the preparation of ligand structures, 2D structure was sketched in chemdraw12.0 and converted to 3D, mol2 format, for each ligand. Hydrogens were added to the structures and used for docking. The Grid center was calculated at x = −20.558, y = −19.574 and z = 127.994 for GABA_A_ receptor, at x = −19.7478, y = 22.417 and z = −14.3006 for SERT transporter and at x = −8.207, y = 9.305 and z = −48.61 for 5-HT_1A_ receptor. The grid size was set to 110 × 110 × 110 xyz points with grid spacing of 0.375Å. For the simulation, default values of quaternation, translation and torsion steps were applied. The Lamarckian Genetic Algorithm with default parameters was applied for minimization. The number of docking runs was 100. The Accelrys Discovery Studio 2020 Client [40,41] and LigandScout were used for the graphical representations of all ligand-protein complexes.

### 3.4. Drug-Likeness

Drug-likeness is one important tool employed for predicting drug-like property. It is designated as an intricate balance of diverse molecular and structural features which plays a pivotal role in establishing whether the specific drug candidate is an oral drug or not. The targeted molecules were appraised for predicting the Drug-likeness based on 5 separate filters namely Egan [42], Ghose [43], Muegge [44], Veber [45] and Lipinski [46] rules accompanying bioavailability and Drug-likeness scores using the Molsoft software and SwissADME program (http://swissadme.ch) (accessed on 10 February 2021) using the ChemAxon’s Marvin JS structure drawing tool.

## 4. Conclusions

A method for the synthesis of new classes of condensed heterocyclic systems, such as pyridofuro[3,2-*d*]pyrrolo[1,2-*a*]pyrimidines, pyridofuro[3,2-*d*]pyrido[1,2-*a*]pyrimidines and pyridofuro[3′,2′:4,5]pyrimido[1,2-*a*]azepines, has been developed. The anticonvulsant activity combined by some psychotropic properties of the new compounds was evaluated.

The biological assays evidenced that some of the studied compounds showed a high anticonvulsant activity by antagonism with pentylenetetrazole being superior to ethosuximide, but inferior to diazepam. The compounds at 100 mg/kg dose increased latency of thiosemicarbazide seizures to 1.3–2.3 times compared with the control and reference drug diazepam. The findings suggest some GABA-ergic mechanism of action of substances. The toxicity of compounds is low and do not induce muscle relaxation in the studied doses. According to the study of psychotropic activity it was found that the selected compounds have an activating behavior and anxiolytic effects on the models of “open field” and EPM, defense mechanism against the fear in contrast to ethosuximide. The data obtained indicate the anxiolytic (anti-anxiety) activity of the derivatives of pyrimidines, especially pronounced in compounds **6n**, **6b**, **7c**. Diazepam at a dose of 2 mg/kg has the same properties, in contrast to ethosuximide. The studied compounds at a dose of 50 mg/kg, increasing the latent time of first immobilization (active swimming time), exhibit some antidepressant effect similarly to diazepam. The results show that the compounds can be effective in various types of human epilepsy associated with mental disorders.

The experimentally active compounds **6b**, **6j**, **6k**, **6n**, **7c**, and **7h** appeared to have good *in-silico* predictions with a Drug-likeness score ranging from 0.26 to 0.97 for compound **6b** without any violation.

## Figures and Tables

**Table 1:** Furo[3,2-*d*]pyrrolo[1,2-*a*]pyrimidines **5**, furo[3,2-*d*]pyrido[1,2-*a*]pyrimidines **6** and furo[3′,2′:4,5]pyrimido[1,2-*a*]azepines **7**.

Compound	X	R	n	R^1^	Yield (%)
**5a**	CH_2_	H	1	Me	68
**5b**	CH_2_	H	1	Et	70
**5c**	O	Me	1	*i*-Pr	65
**5d**	O	Me	1	Ph	69
**6a**	CH_2_	H	0	*i*-Pr	57
**6b**	CH_2_	H	0	*n*-Bu	53
**6c**	CH_2_	H	0	C_6_H_11_	56
**6d**	CH_2_	H	1	Me	61
**6e**	CH_2_	H	1	Et	54
**6f**	CH_2_	H	1	*i*-Pr	58
**6g**	CH_2_	H	1	*i*-Bu	55
**6h**	O	Me	1	Me	60
**6i**	O	Me	1	Et	62
**6j**	O	Me	1	*i*-Pr	57
**6k**	O	Me	1	*n*-Bu	61
**6l**	O	Me	1	*i*-Bu	53
**6m**	O	Me	1	Ph	59
**6n**	O	Me	1	2-furyl	56
**7a**	CH_2_	H	0	C_6_H_11_	68
**7b**	CH_2_	H	0	2-furyl	70
**7c**	CH_2_	H	1	Me	66
**7d**	CH_2_	H	1	Et	65
**7e**	O	Me	1	*i*-Pr	67
**7f**	O	Me	1	*i*-Bu	64
**7g**	O	Me	1	Ph	71
**7h**	O	Me	1	2-furyl	69

**Table 2:** PASS prediction results.

No.	Pa	Pi	Activity	No	Pa	Pi	Activity
**5c**	0.275	0.043	Anticonvulsant	**6k**	0.286	0.129	Anticonvulsant
**5d**	0.423	0.055	Anticonvulsant	**6l**	0.386	0.039	Anticonvulsant
**6b**	0.281	0.121	Anticonvulsant	**6m**	0.430	0.053	Anticonvulsant
**6c**	0.276	0.030	Anticonvulsant	**6n**	0.284	0.127	Anticonvulsant
**6h**	0.364	0.081	Anticonvulsant	**7e**	0.362	0.082	Anticonvulsant
**6i**	0.267	0.142	Anticonvulsant	**7f**	0.336	0.098	Anticonvulsant
**6j**	0.362	0.082	Anticonvulsant	**7g**	0.430	0.053	Anticonvulsant

**Table 3:** Anticonvulsant activity and toxicity of the examined compounds **6b**,**j**,**k**,**n** and **7c**,**h**.

Compound	ED50 * mg/kg (by PTZ Antagonism)	TD50 * mg/kg	LD50 * mg/kg	TI	Latency of Convulsions Induced by TSC, min	
					М ± м	I **
**Control**	–	–	–		69 ± 2.0	1.0
**6b**	35.0 (23.0 ÷ 56.1) ***	>200	450 (375 ÷ 548)	13	123 ± 15.2	1.78
**6j**	35.0 (23.0 ÷ 56.7)	>200	580.0 (464 ÷ 725)	17	90.0 ± 12.3	1.3
**6k**	30.0 (17.6 ÷ 51.0)	>200	620 (504 ÷ 762.6)	21	125 ± 10.8	1.8
**6n**	41.0 (22.5 ÷ 74.6)	>200	930 (808.7 ÷ 1070)	23	158 ± 13.5	2.3
**7c**	42.0 (22.5 ÷ 75.1)	>200	890 (712 ÷ 1112.5)	21	113 ± 10.1	1.64
**7h**	30.0 (17.6 ÷ 51.0)	>200	680 (557 ÷ 829.6)	23	116 ± 16.8	1.68
**Ethosuximide** **(200 mg/kg)**	155 (117.5 ÷ 204.5)	520 (413÷655)	1325 (1200 ÷ 1462)	8.5	118 ± 14.0	1.7
**Diazepam** **(2 mg/kg)**	0.5 (0.4 ÷ 0.7)	2.7 (1.4 ÷ 5.5)	180 (128.5 ÷ 252.0)	360	65 ± 3.5	0.9

* *p* = 0.05 at a probability level I, ** Latent period index. *** Confidence intervals ED50 are shown in parentheses.

**Table 4:** Research activity of compounds **6b**,**j**,**k**,**n** and **7c**,**h** at a dose of 50 mg/kg, ethosuximide at 200 mg/kg and diazepam at a dose of 2 mg/kg in the “open field” model.

Compound	Amount (Absolute Data during 5 min) *		
	Horizontal Displacement	Vertical Displacement	Cells
**Control**	14.4 (11.5 ÷ 18.0)	3.4 (2.8 ÷ 4.1)	0.8 (0.4 ÷ 1.2)
**6b**	57.4 (44.6 ÷ 70.2) **	9.6 (7.3 ÷ 11.9) **	4.0 (2.8 ÷ 5.2) **
**6j**	57.6 (30.9 ÷ 84.3) **	11.0 (7.4 ÷ 14.6) **	4.2 (3.0 ÷ 5.4)**
**6k**	49.6 (39.7 ÷ 62.0) **	9.0 (6.9 ÷ 11.1) **	2.4 (1.3 ÷ 3.5) **
**6n**	64.6 (48.5 ÷ 80.7) **	7.0 (5.8 ÷ 8.4) **	5.0 (2.4 ÷ 7.4) **
**7c**	51.0 (36.6 ÷ 65.4) **	8.5 (4.6 ÷ 12.4) **	3.2 (1.2 ÷ 5.2) **
**7h**	55.0 (42.6 ÷ 67.4) **	12.0 (5.9 ÷ 18.1)**	3.4 (2.1 ÷ 4.7) **
**Ethosuximide**	16.8 (13.4 ÷ 21.0)	3.6 (3.0 ÷ 4.4)	0.6 (0.5 ÷ 0.72)
**Diazepam**	33.6 (27.7 ÷ 40.7) **	6.4 (5.4 ÷ 7.4) **	5.0 (4.0 ÷ 6.25) **

*p ≤ 0.05 at a probability level. ** The differences are statistically significant compared with the control.

**Table 5:** Influence of compounds **6b**,**j**,**k**,**n**; **7c**,**h** and compared drugs on the EPM model (5 min of research).

Compound Dose 50 mg/kg	Time Spent in Closed Arms /s/*	Number of Entries into the Closed Arms *	Time Spent in the Center /s/*	Time Spent in the Open Arms /s/*
**Control**	271.8 (246.2 ÷ 294.4)	5.0 (3.5 ÷ 7.7)	28.1 (23.0 ÷ 33.7)	–
**6j**	212 (184.3 ÷ 243.8) **	2.6 (0.8 ÷ 4.4)	85.0 (51.6 ÷ 118.4) **	–
**6k**	197 (168.3 ÷ 230.5) **	4.4 (1.9 ÷ 6.9)	102.4 (75.2 ÷ 177.6) **	–
**7h**	209.8 (182.4 ÷ 241) **	8.0 (5.5 ÷ 10.5)	90.2 (50.5 ÷ 129.9) **	–
**6n**	182.0 (155.6 ÷ 209) **	6.2 (4.0 ÷ 8.2)	118.0 (75.1 ÷ 193.1) **	21.8 (18.3 ÷ 40) **
**6b**	166 (123.7 ÷ 208.7) **	6.0 (4.2 ÷ 7.8)	116 (81.7 ÷ 115.9) **	17.0 (5.9 ÷ 28.1) **
**7c**	178.0 (130.9 ÷ 225) **	4.0 (2.2 ÷ 6.2)	108.0 (80.1 ÷ 135.9) **	14.0 (3.3 ÷ 24.7) **
**Ethosuximide** **(200 mg/kg)**	247.2 (212.9 ÷ 277.5)	8.1 (5.6 ÷ 10.6)	52.8 (44.0 ÷ 63.4) **	–
**Diazepam ** **(2 mg/kg)**	257.5 (226.2 ÷ 288.8)	5.5 (4.58 ÷ 6.6)	42.5 (34.8 ÷ 51.85) **	57 (47.5 ÷ 68.4) **

*p ≤ 0.05 at a probability level; ** The differences are statistically significant compared with the control.

**Table 6:** Effect of compounds **6b**,**j**,**k**,**n** and **7c**,**h** and reference preparations on “forced swimming” (study for 6 min).

Compound	Dose mg/kg	Time of Active Swimming (s), Latent Period First Immobilization *	Total Time of Immobilization (s) *	Total Time of Active Swimming (s) *
**Control**	–	127.5 (106.3 ÷ 155.6)	42.8 (29.4 ÷ 57.2)	317.2 (302.8 ÷ 331.6)
**6b**	50	132.0 (111.4 ÷ 152.6)	56.0 (28.6 ÷ 83.4)	304.0 (265.9 ÷ 342.1)
**6j**	50	168.0 (140 ÷ 201.6)	135.2 (87.4 ÷ 183.0) **	140.8 (84.3 ÷ 196.7) **
**6k**	50	188.0 (163.5 ÷ 216.2) **	79.0 (59.5 ÷ 98.5) **	281.0 (226.8 ÷ 355.2)
**6n**	50	146.0 (120.0 ÷ 175.2)	44.0 (35.9 ÷ 52.1)	316.0 (293.4 ÷ 338.6)
**7c**	50	164.0 (138.3 ÷ 189.7)	49.0 (36.3 ÷ 61.7)	311.0 (275.5 ÷ 346.5)
**7h**	50	172.0 (139.4 ÷ 204.6)	81.0 (65.1 ÷ 96.9) **	279.0 (263.2 ÷ 294.8) **
**Ethosuximi** **de**	200	125 (102.4 ÷ 152.5)	98 (75.3 ÷ 127.4) **	262 (199.9 ÷ 324.1)
**Diazepam**	2	174 (144.0 ÷ 204.0)	24 (20.0 ÷ 28.8) **	336 (282.6 ÷ 389.4)

*p ≤ 0.05 at a probability level. ** The differences are statistically significant compared with the control.

**Table 7:** Molecular docking scoring function that estimates free energy of binding (kcal/mol) into GABA_A_ receptor (PDB code: 4COF).

Compound	Est. Binding Energy (kcal/mol)	I-H	Residues Involved in Hydrogen Bond Formation	Hydrophobic Interactions	Aromatic Interactions
**6b**	−8.02	1	Thr202	Ala25, Tyr62, Leu99, Phe200	Phe200
**6j**	−7.14	1	Tyr202	Tyr62, Leu99, Phe200	Phe200
**6k**	−7.95	1	Thr202	Thr176, Phe200, Ala201	-
**6n**	−6.37	1	Thr62	Leu99, Phe200	-
**7c**	−6.25	-	-	Ty157, Thr202, Tyr205	-
**7h**	−7.63	1	Thr62	Leu99, Phe200, Thr202	-
**Diazepam**	−8.90	1	Thr202	Tyr62, Thr176, Phe200, Ala201, Tyr205	Phe200

**Table 8:** Molecular docking scoring function that estimates free energy of binding (kcal/mol) in SERT transporter (PDB ID: 3F3A).

No.	Est. Binding Energy (kcal/mol)	I-H	Residues Involved in Hydrogen Bond Formation	Residues Involved in Van der Waals Interactions	Residues Involved in pi-pi Interactions
**6b**	−8.96	1	Arg7	Arg431, Gly432, Gly433, Lys436, Asp267	Asp265, Ile434
**6j**	−7.93	1	Arg7	Asp267, Gly432, Gly433, Lys436	Lys264
**6k**	−7.26	-	-	Arg7, Arg431, Gly432, Gly433	Asp265, Lys264
**6n**	−10.25	2	Arg7, Gln266	Asp267, Gly432, Gly433	Arg7, Arg263, Lys264, Asp265, Ile434
**7c**	−8.72	1	Arg7	Asp267, Gly432, Gly433	Asp265, Lys264
**7h**	−7.85	1	Asp265	Arg7, Gly432, Lys436	Asp265

**Table 9:** Molecular docking free binding energies (kcal/mol) in 5-HT_1A_ receptor (PDB ID: 3NYA).

No.	Est. Binding Energy (kcal/mol)	I-H	Residues Involved in Hydrogen Bond Formation	Residues Involved in Van der Waals Interactions	Residues Involved in pi-pi Interactions
**6b**	−10.16	2	Asn312, Tyr316	Trp109, Asp113, Thr118, Ser204, Phe108, Trp286	Val114, Val117
**6j**	−7.35	1	Tyr308	Asp113, Thr118, Ala200, Ser204, Trp286, Asn312	Val114, Phe290
**6k**	−8.13	1	Asn312	Asp113, Ala200, Ser207, Phe108, Trp286, Tyr308	Val114, Val117, Tyr199
**6n**	−11.21	3	Asn293, Tyr308, Tyr316	Trp109, Thr110, Asp113, Thr118, Ala200, Ser204, Ser207, Phe108, Trp286, Asn312	Val114, Val117, Phe193, Tyr199, Phe290
**7c**	−9.67	2	Tyr308, Tyr316	Asp113, Ser204, Ser207, Phe108, Asn312	Val114, Phe193, Phe290
**7h**	−6.88	-	-	Trp109, Thr110, Asp113, Ala200, Ser204, Phe108, Trp286, Asn312	Val114, Val117, Phe290
**Alprenolol**	−13.19	4	Asp113, Asn312, Tyr316	Met82, Thr110, Tyr118, Ser203, Ser207, Trp286, Phe289, Ala200, Tyr308	Trp109, Val114, Phe193, Tyr199, Phe290

**Table 10:** Drug likeness predictions and Physicochemical-Pharmacokinetic/ADME properties of tested compounds.

No.	MW	Number of HBA ^a^	Number of HBD ^b^	Log *P*_o/w_ (iLOGP) ^c^	Log S ^d^	TPSA ^e^	BBB Permeant ^f^	Lipinski, Ghose, Veber, Egan, and Muegge Violations	Bioavailability SCORE	Drug-Likeness Model Score
**5a**	295.34	4	0	2.83	Moderately soluble	60.92	Yes	0	0.55	0.61
**5b**	309.36	4	0	3.11	Moderately soluble	60.92	Yes	0	0.55	0.90
**5c**	323.35	5	0	0	Moderately soluble	70.15	No	0	0.55	0.33
**5d**	357.36	5	0	0	Poorly soluble	70.15	Yes	0	0.55	0.33
**6a**	323.39	4	0	3.44	Moderately soluble	60.92	Yes	0	0.55	0.61
**6b**	337.42	4	0	3.60	Poorly soluble	60.92	Yes	0	0.55	0.97
**6c**	363.45	4	0	3.80	Poorly soluble	60.92	Yes	0	0.55	0.64
**6d**	309.36	4	0	3.07	Moderately soluble	60.92	Yes	0	0.55	0.57
**6e**	323.39	4	0	3.31	Moderately soluble	60.92	Yes	0	0.55	0.63
**6f**	337.42	4	0	3.56	Moderately soluble	60.92	Yes	0	0.55	0.55
**6g**	351.44	4	0	3.68	Poorly soluble	60.92	Yes	0	0.55	1.01
**6h**	339.39	5	0	3.28	Moderately soluble	70.15	Yes	0	0.55	0.22
**6i**	353.41	5	0	3.48	Poorly soluble	70.15	Yes	0	0.55	0.51
**6j**	367.44	5	0	3.73	Poorly soluble	70.15	Yes	0	0.55	0.57
**6k**	381.47	5	0	3.99	Poorly soluble	70.15	Yes	0	0.55	0.52
**6l**	381.47	5	0	4.00	Poorly soluble	70.15	Yes	0	0.55	0.54
**6m**	401.46	5	0	3.76	Poorly soluble	70.15	Yes	0	0.55	0.33
**6n**	391.42	6	0	3.62	Poorly soluble	83.29	Yes	0	0.55	0.26
**7a**	377.48	4	0	3.95	Poorly soluble	60.92	Yes	0	0.55	0.64
**7b**	361.39	5	0	3.49	Poorly soluble	74.06	Yes	0	0.55	0.53
**7c**	323.29	4	0	3.24	Moderately soluble	60.92	Yes	0	0.55	0.57
**7d**	337.42	4	0	3.43	Poorly soluble	60.92	Yes	0	0.55	0.51
**7e**	381.47	5	0	3.84	Poorly soluble	70.15	Yes	0	0.55	0.54
**7f**	395.49	5	0	4.05	Poorly soluble	70.15	Yes	0	0.55	0.66
**7g**	415.48	5	0	3.93	Poorly soluble	70.15	Yes	0	0.55	0.33
**7h**	405.45	6	0	3.77	Poorly soluble	83.29	No	0	0.55	0.26

(a) number of hydrogen bond acceptors; (b) number of hydrogen bond donors; (c) lipophilicity; (d) Water solubility (SILICOS-IT [S=Soluble]); (e) topological polar surface area (Å^2^); (f) Blood Brain Barrier permeant.

## Data Availability

The study did not report any data.

## Supplementary Materials

The following are available online. The copies of ^1^H-NMR and ^13^C-NMR spectra for all new synthesized compounds have been submitted along with the manuscript.

## Sample Availability

Samples of the compounds **1**–**7** are available from the authors.
