# Supplementary material for: Synthesis and Neurotropic Activity of New Heterocyclic Systems: Pyridofuro[3,2-d]pyrrolo[1,2-a]pyrimidines, Pyridofuro[3,2-d]pyrido[1,2-a]pyrimidines and Pyridofuro[3′,2′:4,5]pyrimido[1,2-a]azepines
Source: Molecules. 2021 Jun 1;26(11):3320. doi: 10.3390/molecules26113320 (PMC8198642; doi:10.3390/molecules26113320)

Supporting Information File

Synthesis and neurotropic activity of new heterocyclic systems: pyridofuro[3,2-*d*]-pyrrolo[1,2-*a*]pyrimidines, pyridofuro[3,2-*d*]pyrido[1,2-*a*]pyrimidines and pyridofuro[3',2':4,5]pyrimido[1,2-*a*]azepines

Samvel N. Sirakanyan[1],* Domenico Spinelli[2],* Athina Geronikaki[3],* Victor Kartsev[4], Elmira K. Hakobyan[1], Anthi Petrou[3], Ruzanna G. Paronikyan[1], Ivetta M. Nazaryan[1], Hasmik H. Akopyan[1], Anush A. Hovakimyan[1]

1 Scientific Technological Center of Organic and Pharmaceutical Chemistry of National Academy of Science of Republic of Armenia, Institute of Fine Organic Chemistry of A.L. Mnjoyan, Armenia 0014, Yerevan, Ave. Azatutyan 26; e-mail: shnnr@mail.ru
2 Dipartimento di Chimica G. Ciamician, Alma Mater Studiorum-Università di Bologna, Via F. Selmi 2, Bologna 40126, Italy; e-mail: domenico.spinelli@unibo.it
3 Aristotle University of Thessaloniki, School of Pharmacy, Thessaloniki 54124, Greece; e-mail: geronik@pharm.auth.gr
4 InterBioScreen, a/ya 218, Moscow 119019, Russia; e-mail: vkartsev@ibscreen.chg.ru

*Corresponding authors Emails:

Domenico Spinelli – domenico.spinelli@unibo.it

Athina Geronikaki – geronik@pharm.auth.gr

Samvel Sirakanyan – shnnr@mail.ru

The copies of ^{1}H and ^{13}C NMR spectra of all new synthesized compounds

2a

Molecular Structure Research Centre, Yerevan, Armenia, Varian Mercury-300VX

H1 300.088 MHz, nt = 16, np = 32000, temp = 30.0 C, lb = -0.2, solvent = DMSO/CCl4 1/3

Dec 9 2020

HA-286

ANUSH_TEMA ha-286

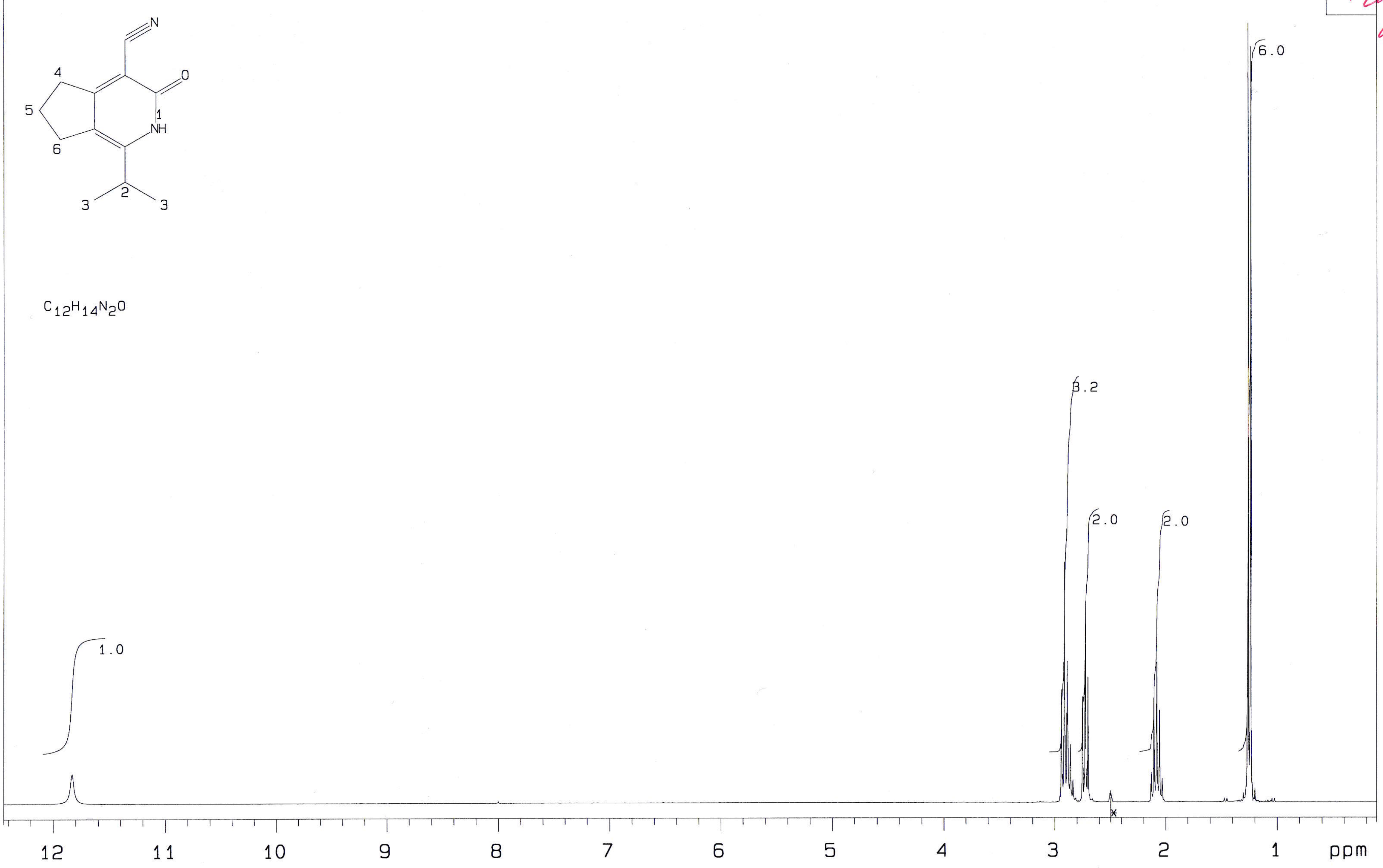

2a

Molecular Structure Research Centre, Yerevan, Armenia, Varian Mercury-300VX

C13 75.465 MHz, nt = 336, np = 19998, temp = 30.0 C, lb = 1.0, solvent = DMSO/CCl4 1/3

Dec 9 2020

ANUSH_TEMA ha-286

HA-286

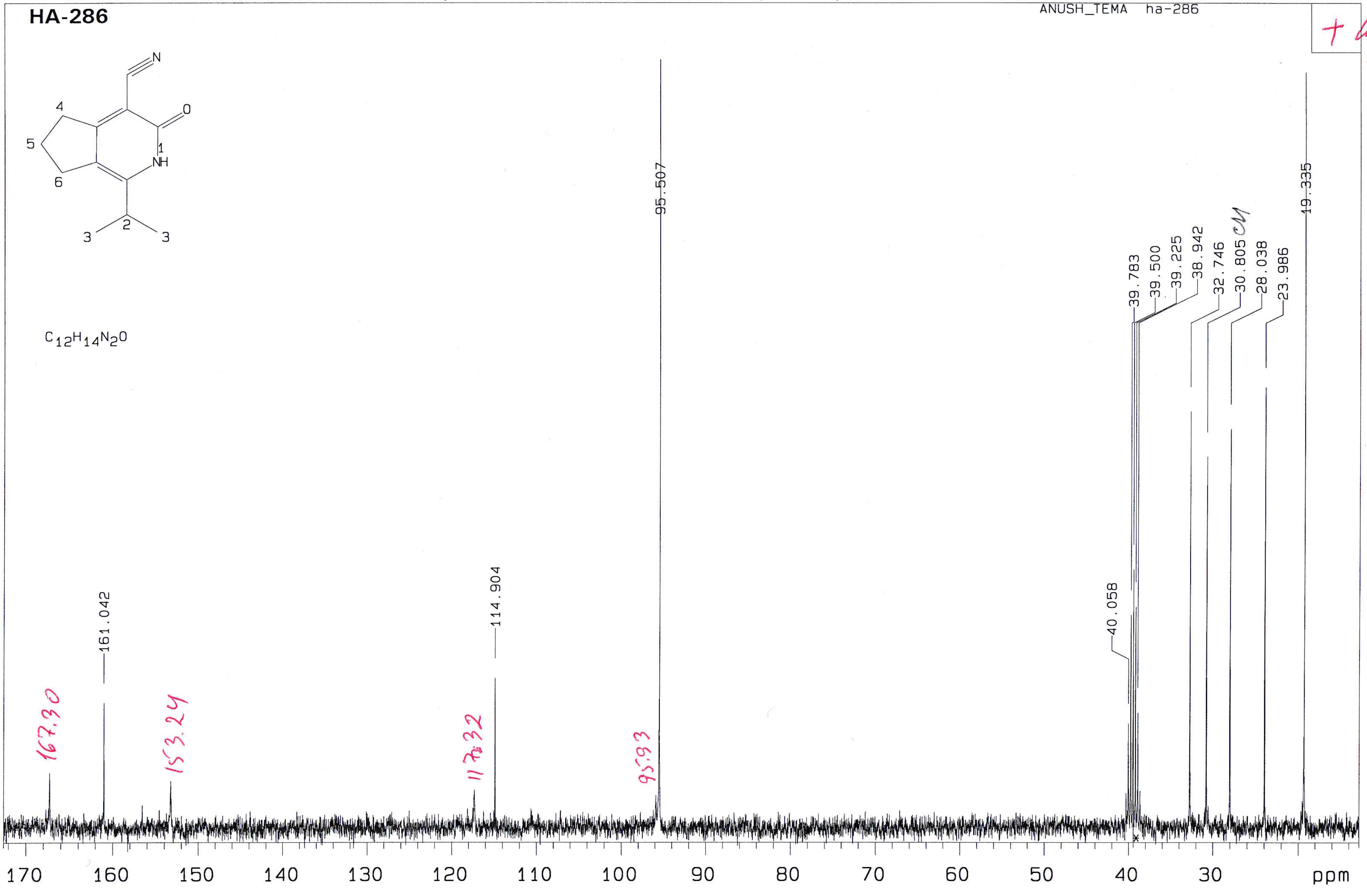

+

2b

Molecular Structure Research Centre, Yerevan, Armenia, Varian Mercury-300VX

H1 300.088 MHz, nt = 16, np = 32000, temp = 30.0 C, lb = -0.2, solvent = DMSO/CCl4 1/3

Dec 8 2020

SS-006

ANUSH_TEMA ss-006

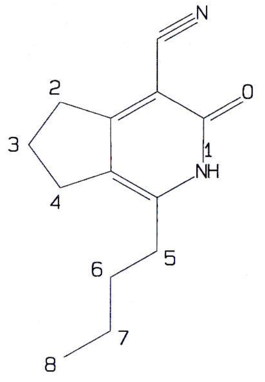

$C_{13}H_{16}N_2O$

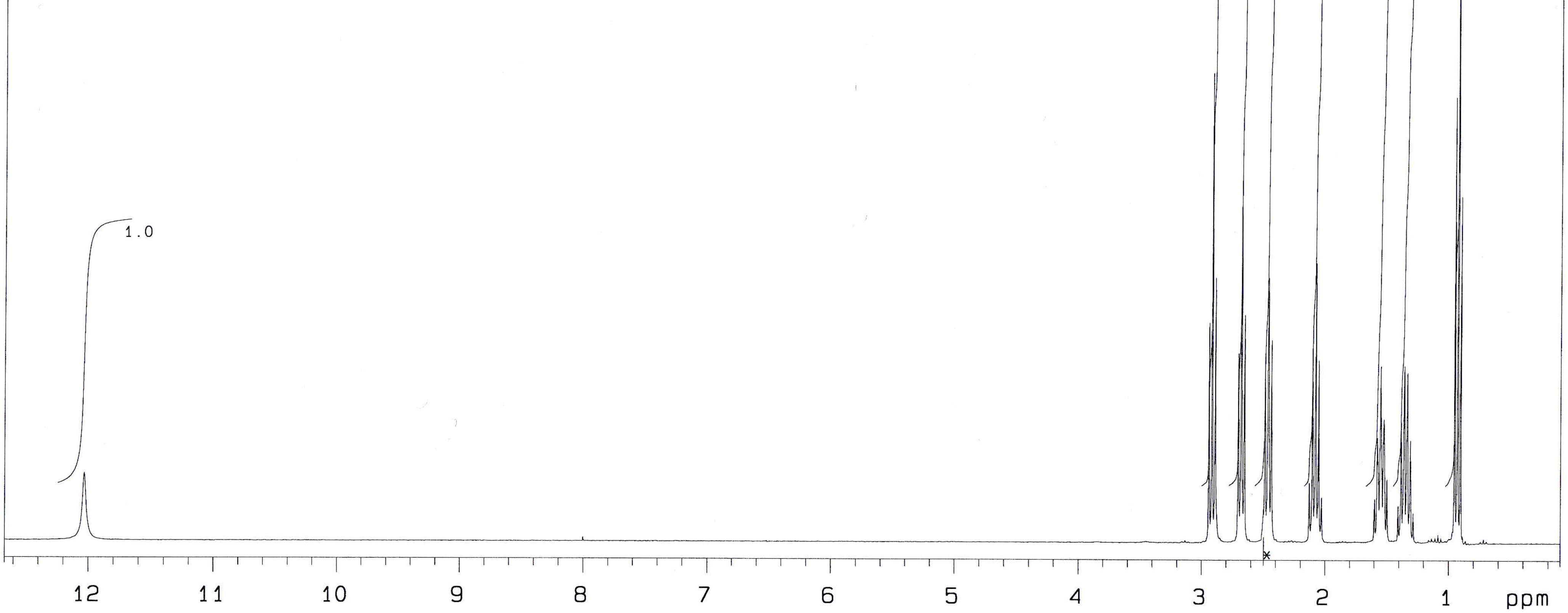

2b

Molecular Structure Research Centre, Yerevan, Armenia, Varian Mercury-300VX

C13 75.465 MHz, nt = 128, np = 19998, temp = 30.0 C, lb = 1.0, solvent = DMSO/CCl4 1/3

Dec 8 2020

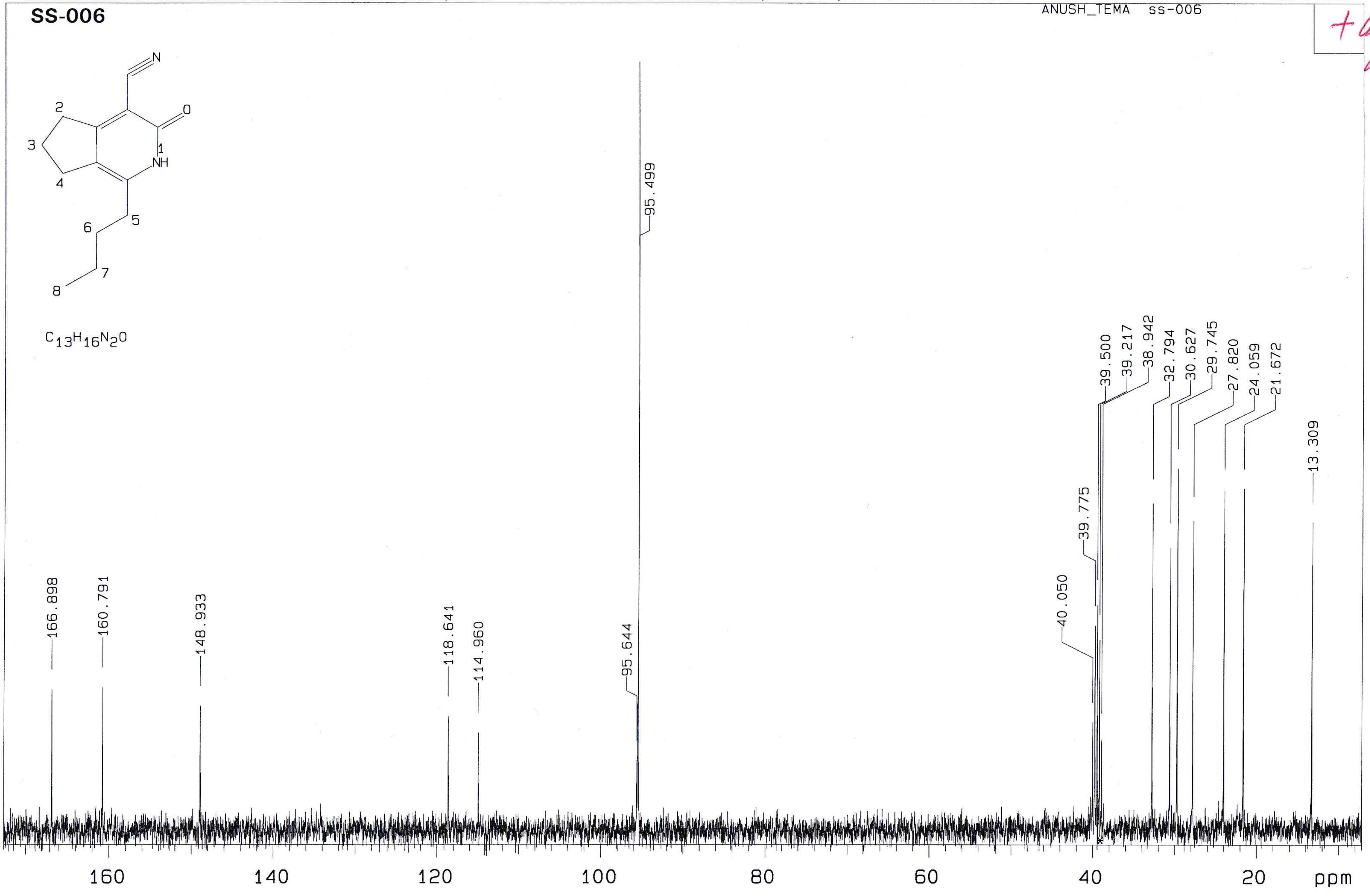

2c

Molecular Structure Research Centre, Yerevan, Armenia, Varian Mercury-300VX

H1 300.088 MHz, nt = 16, np = 32000, temp = 30.0 C, lb = -0.2, solvent = DMSO/CCl4 1/3

Dec 7 2020

HA-046

ANUSH_TEMA ha-046

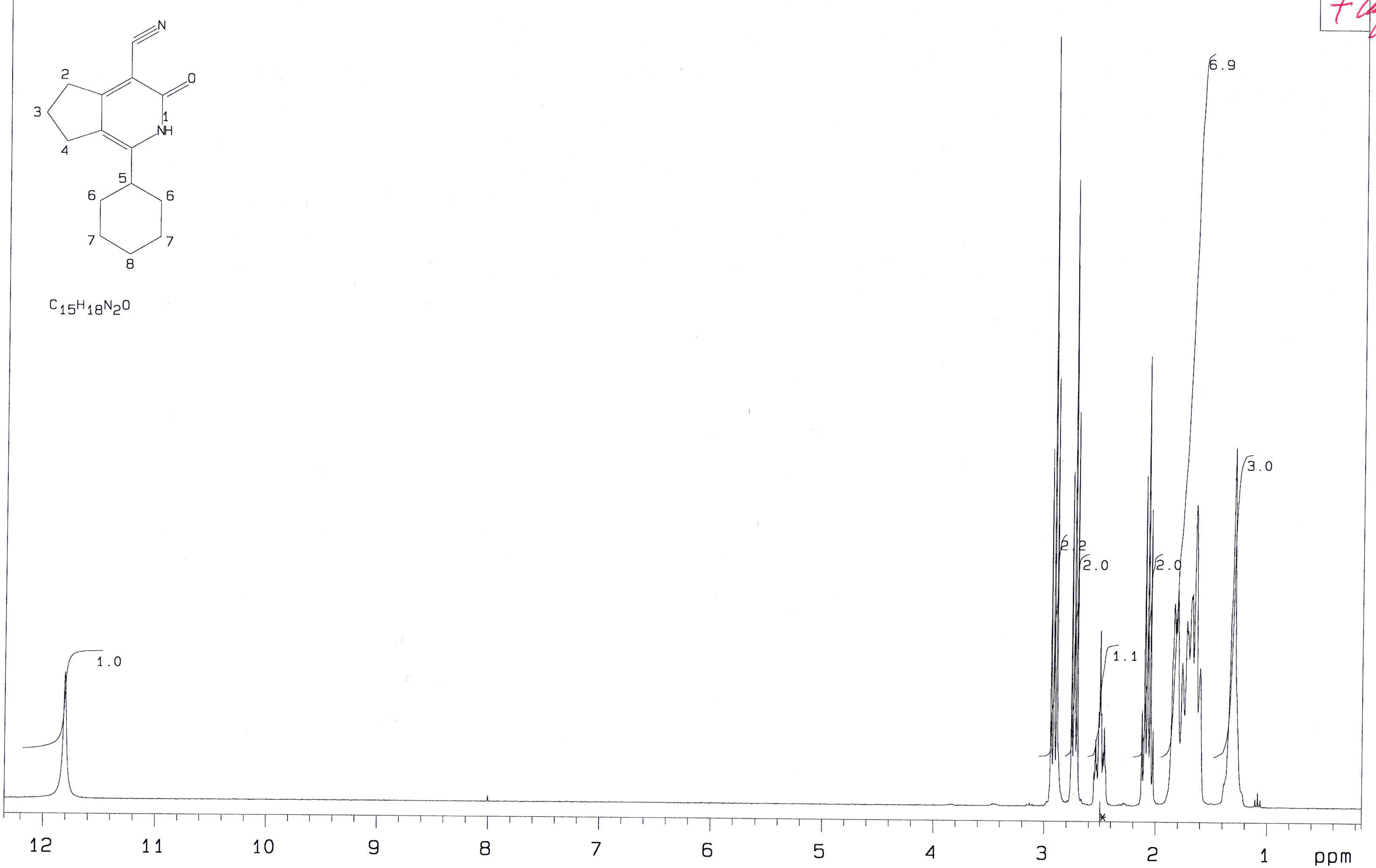

2c

Molecular Structure Research Centre, Yerevan, Armenia, Varian Mercury-300VX

C13 75.464 MHz, nt=320, np=19998, temp=30.0 C, lb=1.0, solvent=CDCL3

Dec 7 2020

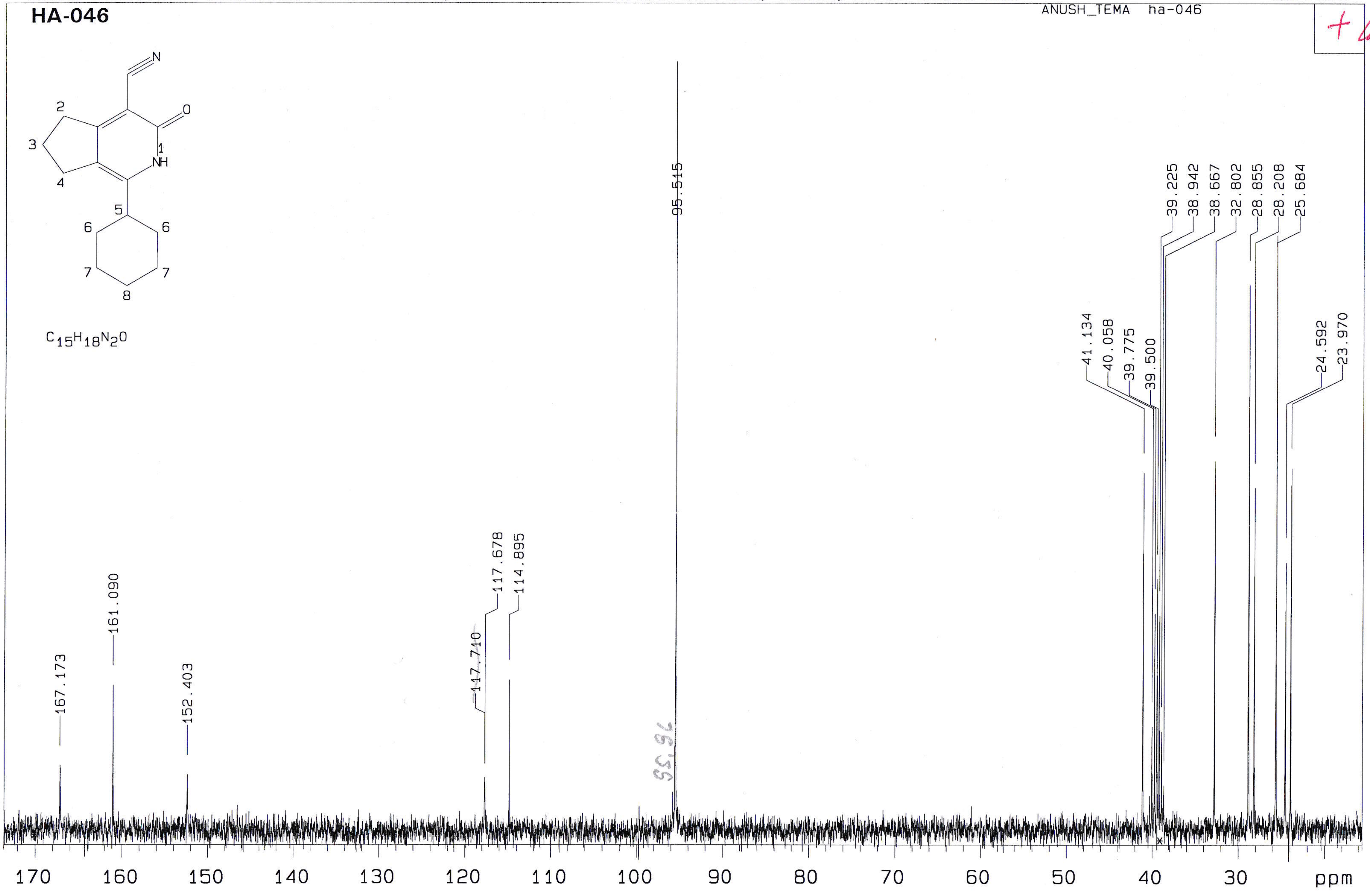

2d

Molecular Structure Research Centre, Yerevan, Armenia, Varian Mercury-300VX

H1 300.077 MHz, nt = 16, np = 16000, temp = 30.0 C, lb = -0.2, solvent = DMSO/CCl4 1/3

Aug 17 2007

MAR-068

SAMV_07 mar-068

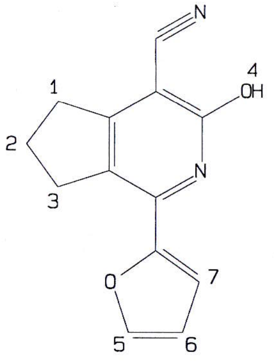

$C_{13}H_{10}N_2O_2$

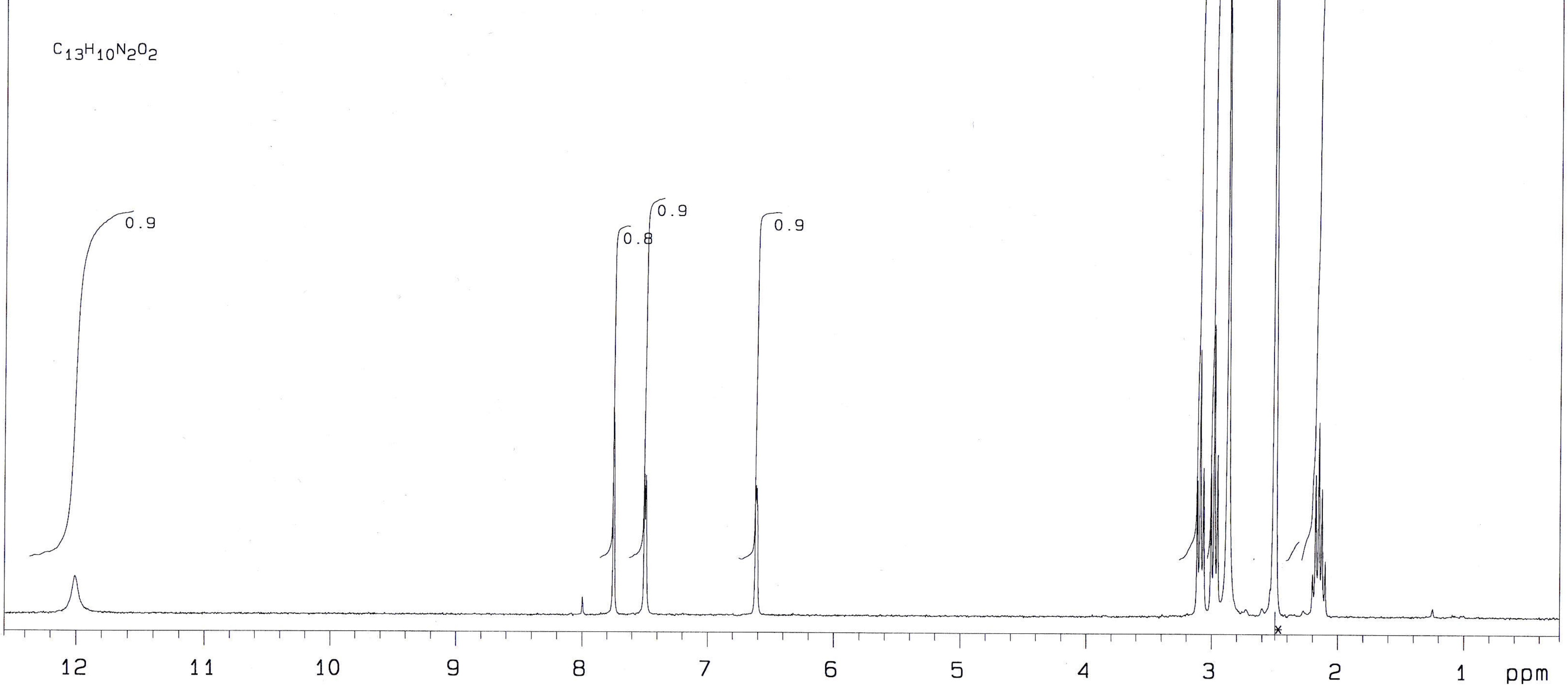

+

2d

Molecular Structure Research Centre, Yerevan, Armenia, Varian Mercury-300VX

C13 75.462 MHz, nt=45936, np=19998, temp=30.0 C, lb=1.0, solvent=DMSO/CCl4 1/3

Jan 26 2016

SAMV_16 he-054

HE-054

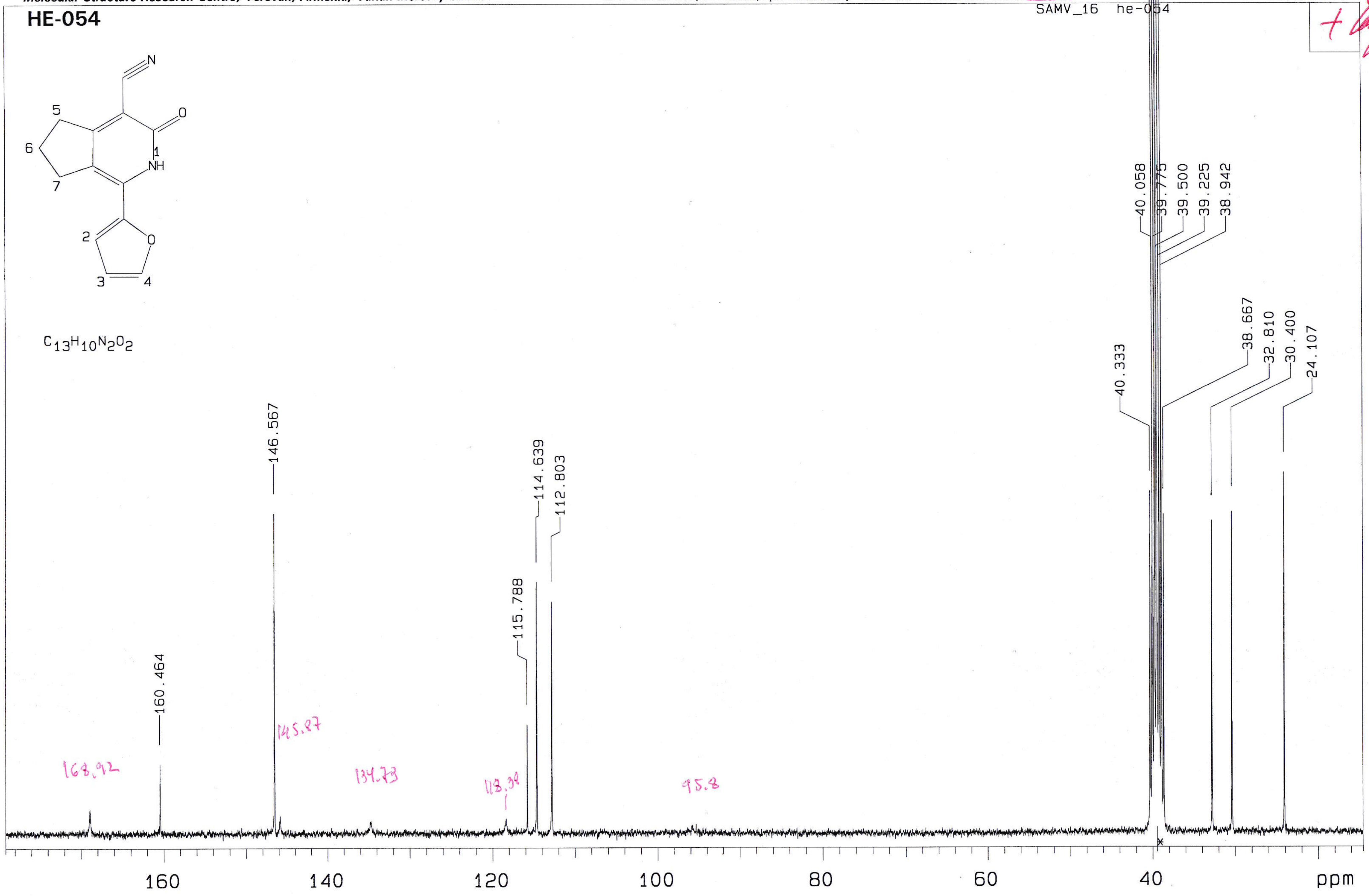

2g

Molecular Structure Research Centre, Yerevan, Armenia, Varian Mercury-300VX

H1 300.088 MHz, nt = 16, np = 32000, temp = 30.0 C, lb = -0.2, solvent = DMSO/CCl4 1/3

Dec 9 2020

ANUSH_TEMA ha-323

HA-323

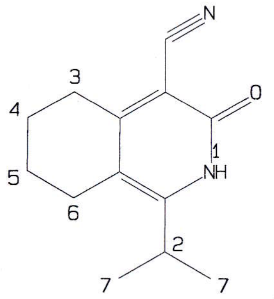

$C_{13}H_{16}N_2O$

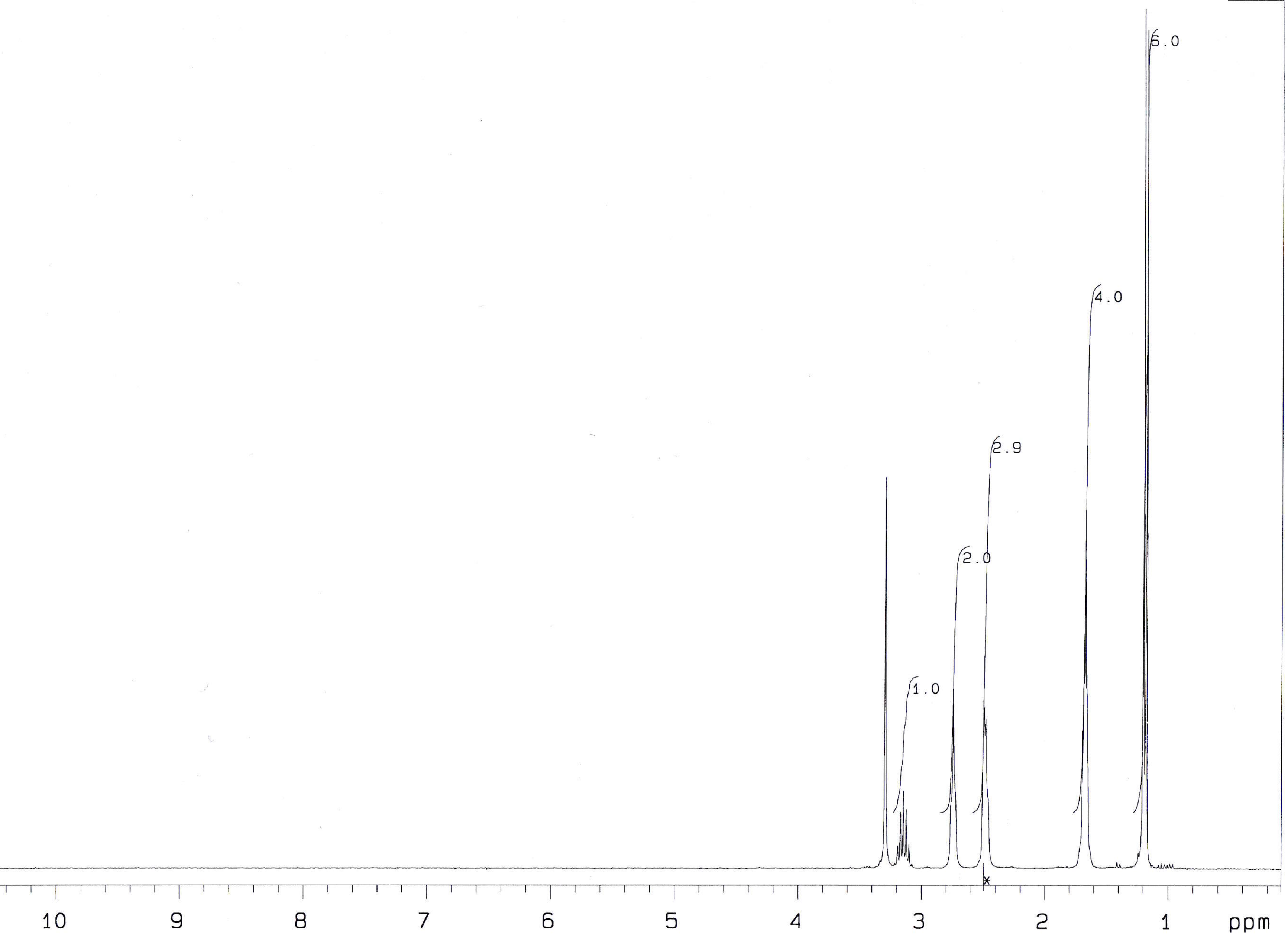

2h

Molecular Structure Research Centre, Yerevan, Armenia, Varian Mercury-300VX H1 300.088 MHz, nt = 16, np = 32000, temp = 30.0 C, lb = -0.2, solvent = DMSO/CCl4 1/3 Dec 15 2020

T20-004

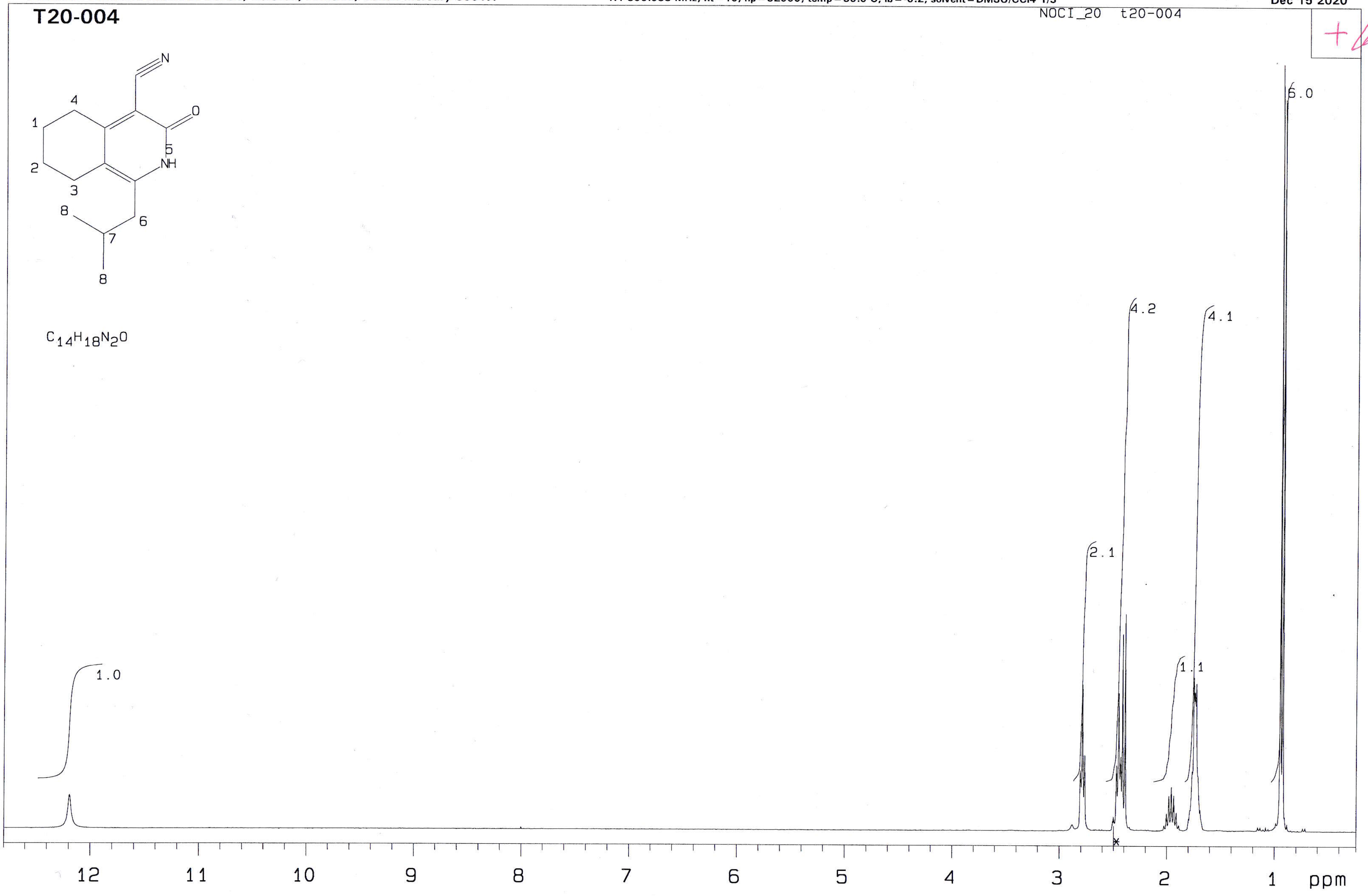

2h

Molecular Structure Research Centre, Yerevan, Armenia, Varian Mercury-300VX

C13 75.465 MHz, nt = 208, np = 19998, temp = 30.0 C, lb = 1.0, solvent = DMSO/CCl4 1/3

Dec 15 2020

T20-004

NOCI_20 t20-004

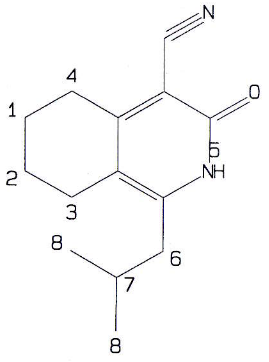

$C_{14}H_{18}N_2O$

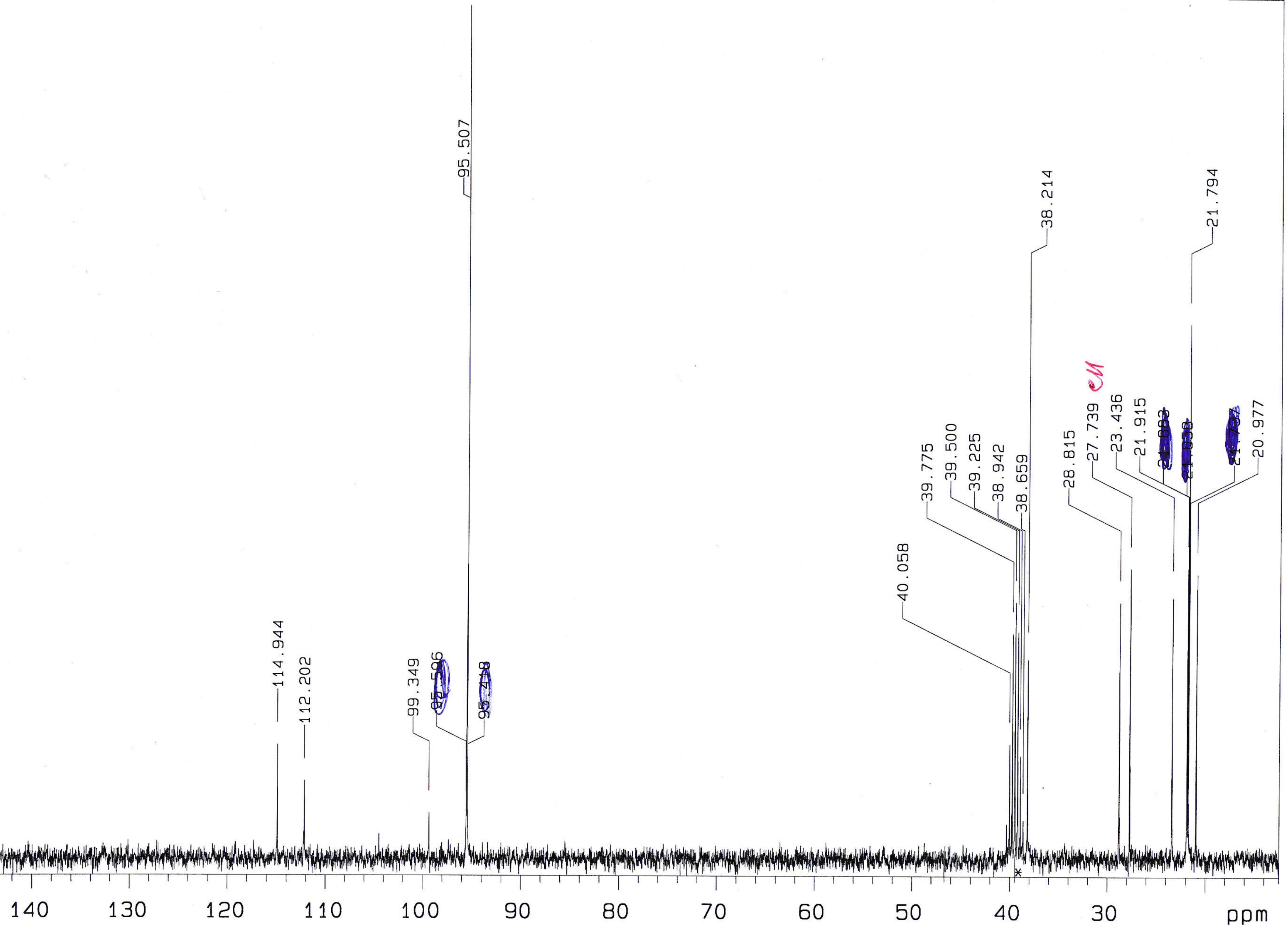

3c

Molecular Structure Research Centre, Yerevan, Armenia, Varian Mercury-300VX

H1 300.088 MHz, nt = 16, np = 32000, temp = 30.0 C, lb = -0.2, solvent = DMSO/CCl4 1/3

Dec 10 2020

HA-050

ANUSH_TEMA ha-050

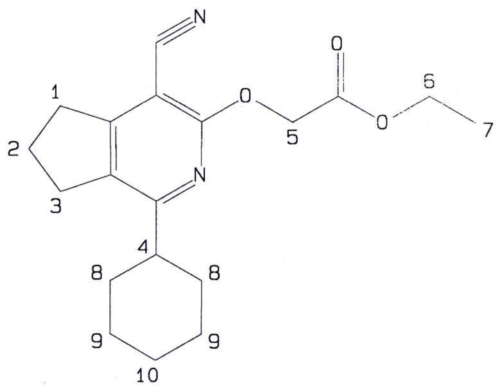

$C_{19}H_{24}N_2O_3$

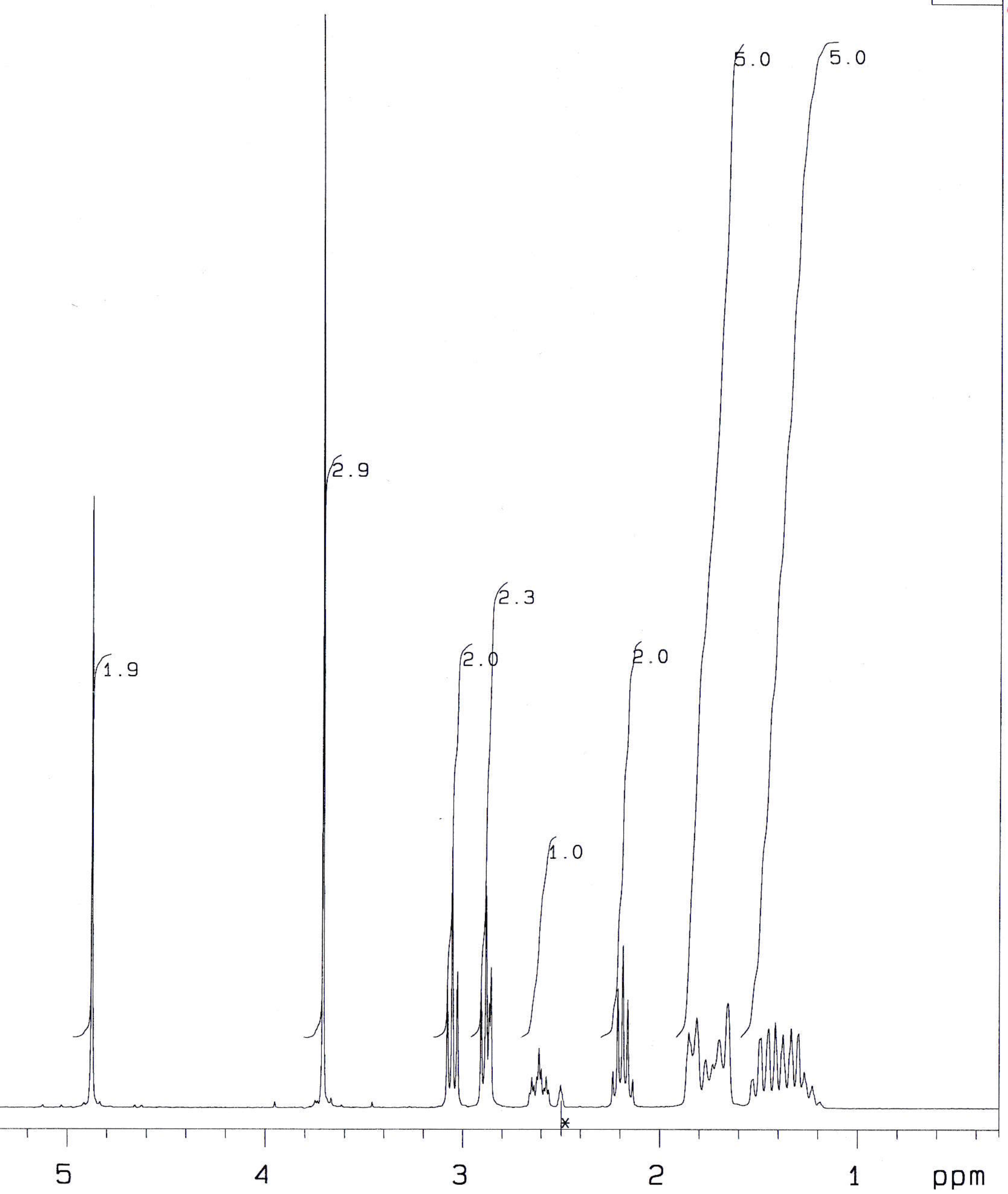

3c

Molecular Structure Research Centre, Yerevan, Armenia, Varian Mercury-300VX

C13 75.465 MHz, nt = 128, np = 19998, temp = 30.0 C, lb = 1.0, solvent = DMSO/CCl4 1/3

Dec 10 2020

HA-050

ANUSH_TEMA ha-050

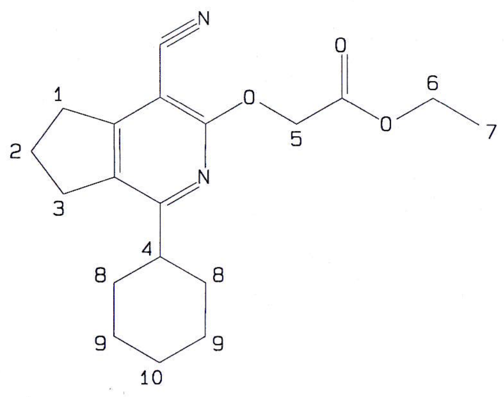

$C_{19}H_{24}N_2O_3$

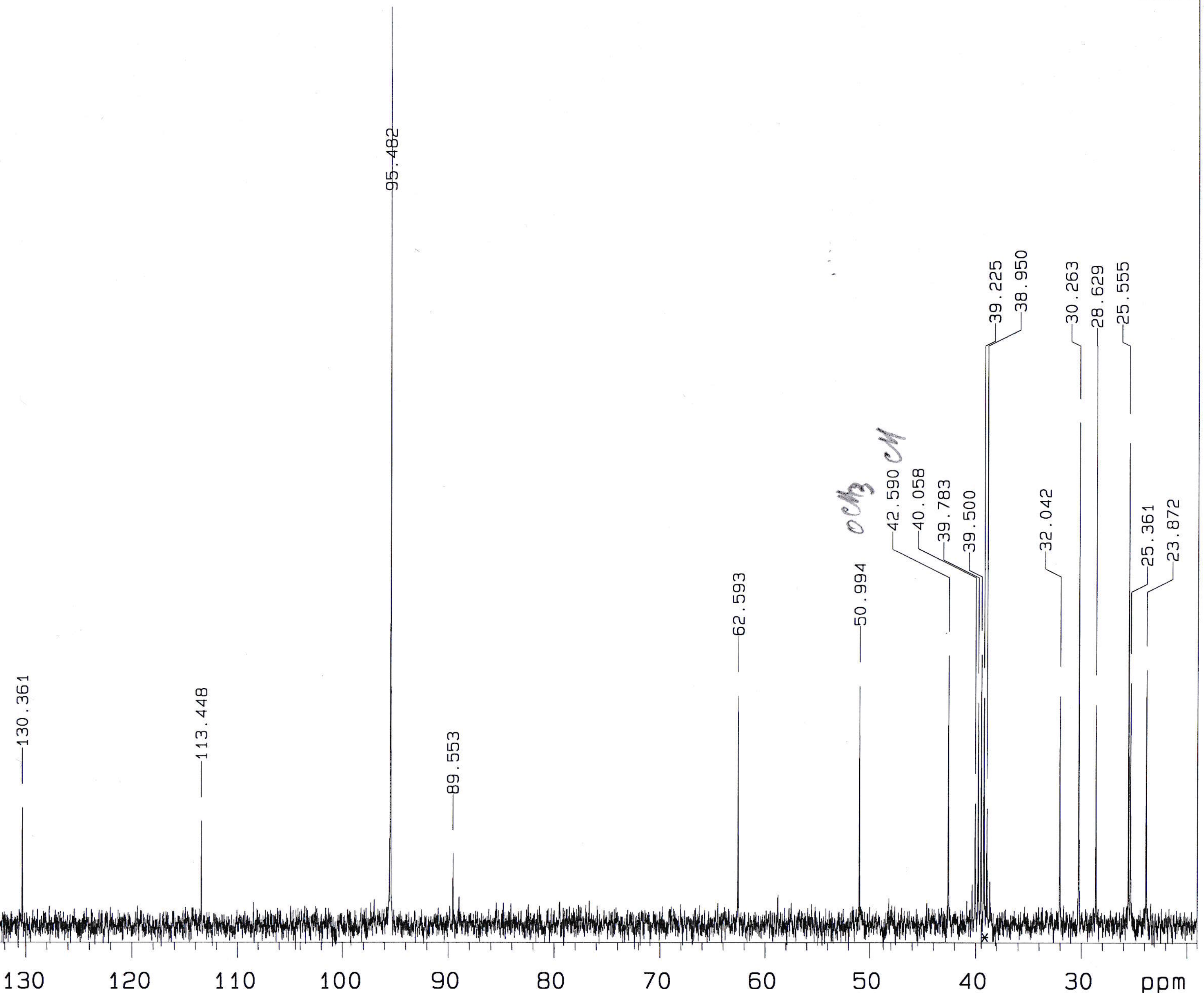

3n

Molecular Structure Research Centre, Yerevan, Armenia, Varian Mercury-300VX

H1 300.088 MHz, nt = 16, np = 32000, temp = 30.0 C, lb = -0.2, solvent = DMSO/CCl4 1/3

Dec 8 2020

AE0135-0403

ANUSH_TEMA ae0135-0403

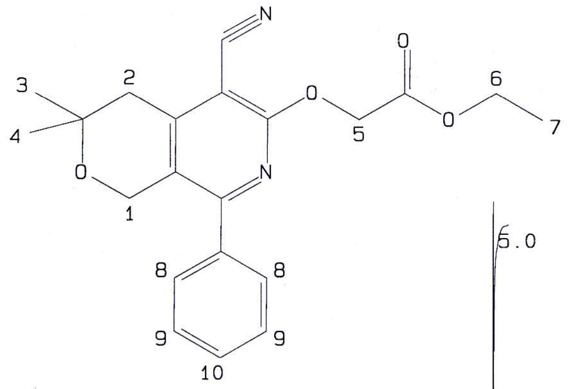

$C_{21}H_{22}N_2O_4$

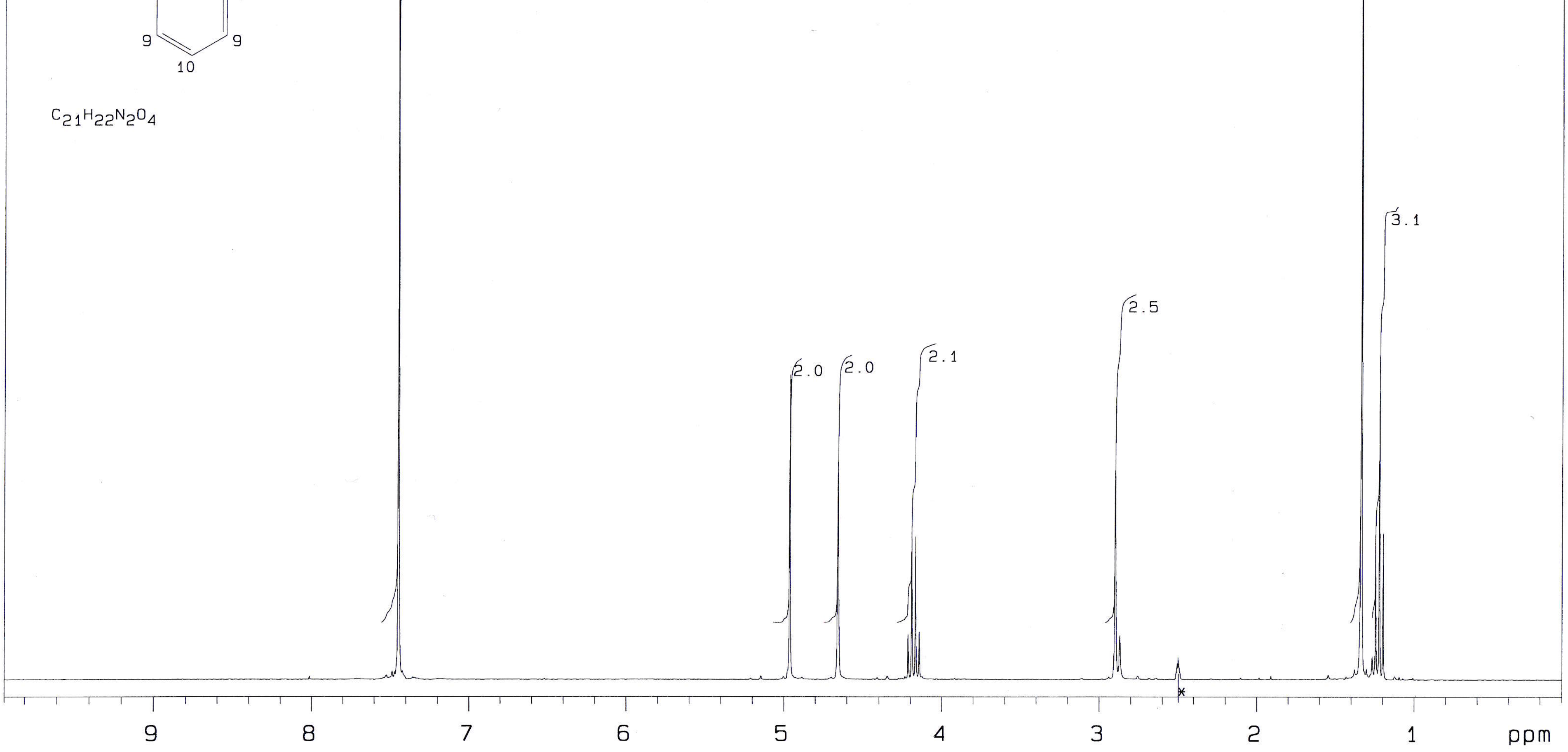

3n

Molecular Structure Research Centre, Yerevan, Armenia, Varian Mercury-300VX

C13 75.465 MHz, nt = 256, np = 19998, temp = 30.0 C, lb = 1.0, solvent = DMSO/CCl4 1/3

Dec 8 2020

AE0135-0403

ANUSH_TEMA ae0135-0403

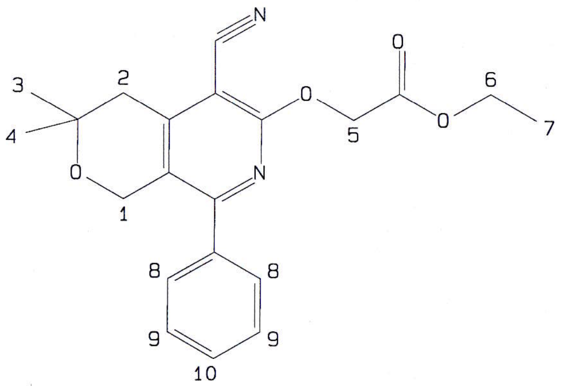

$C_{21}H_{22}N_2O_4$

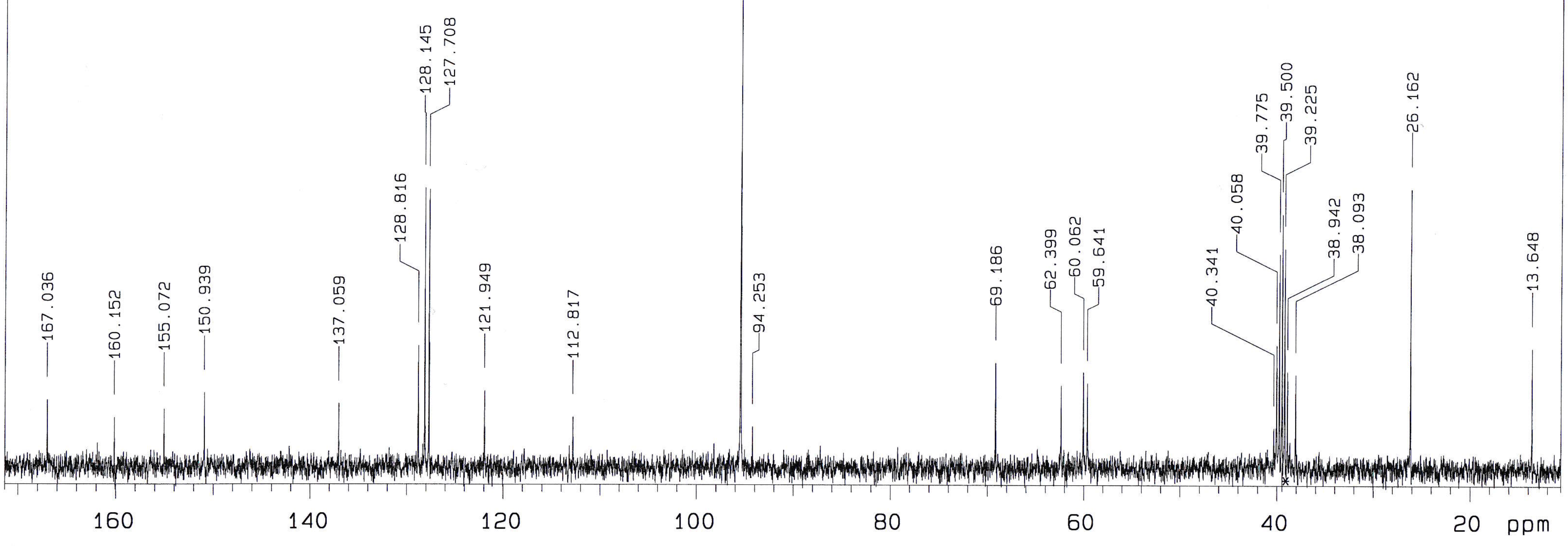

4C

Molecular Structure Research Centre, Yerevan, Armenia, Varian Mercury-300VX

H1 300.088 MHz, nt = 16, np = 32000, temp = 30.0 C, lb = -0.2, solvent = DMSO/CCl4 1/3

Dec 8 2020

ANUSH_TEMA ha-051

HA-051

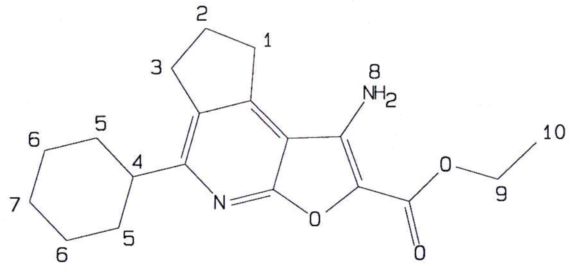

$C_{19}H_{24}N_2O_3$

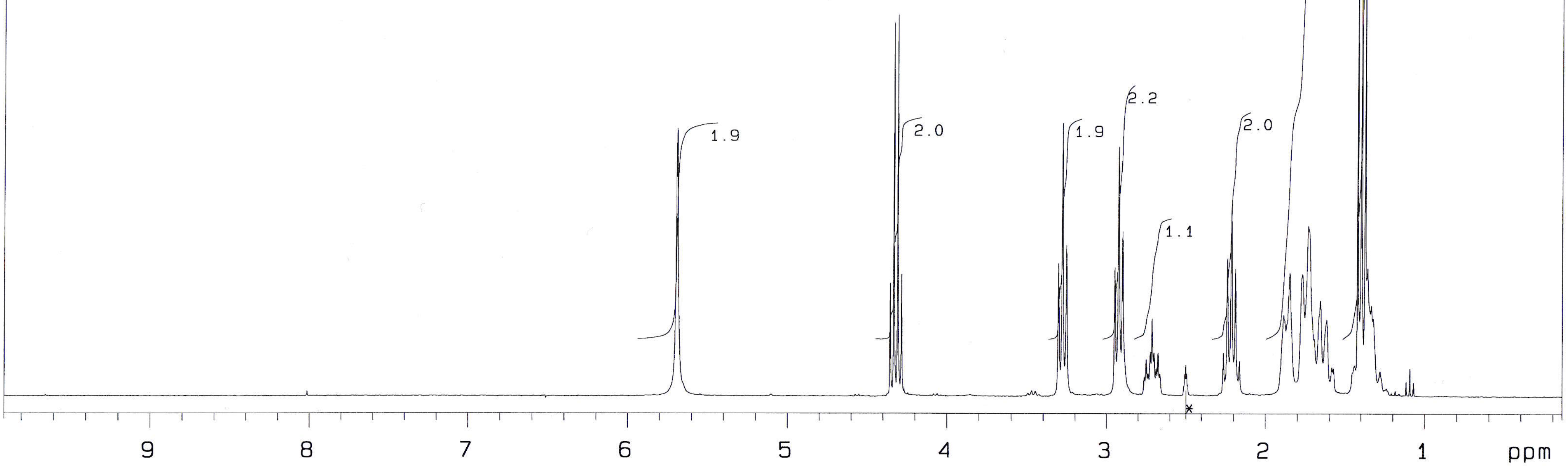

4c

Molecular Structure Research Centre, Yerevan, Armenia, Varian Mercury-300VX

C13 75.465 MHz, nt = 272, np = 19998, temp = 30.0 C, lb = 1.0, solvent = DMSO/CCl4 1/3

Dec 8 2020

HA-051

ANUSH_TEMA ha-051

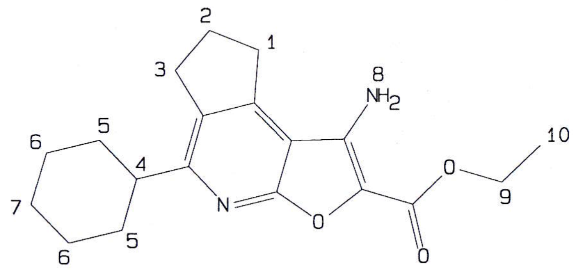

$C_{19}H_{24}N_2O_3$

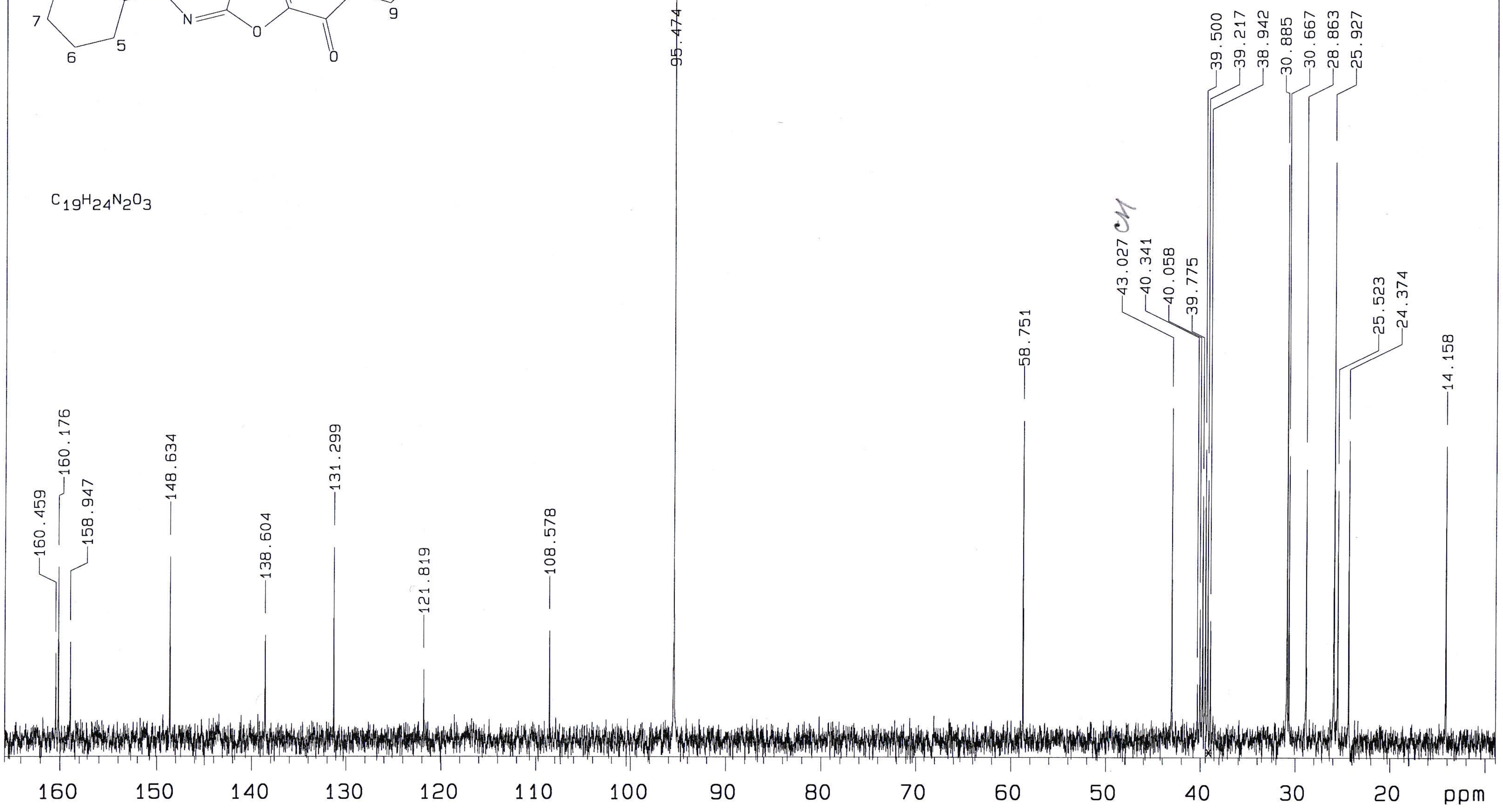

4f

Molecular Structure Research Centre, Yerevan, Armenia, Varian Mercury-300VX

H1 300.088 MHz, nt = 16, np = 32000, temp = 30.0 C, lb = -0.2, solvent = DMSO/CCl4 1/3

Mar 31 2017

SAMV_17 he-180

HE-180

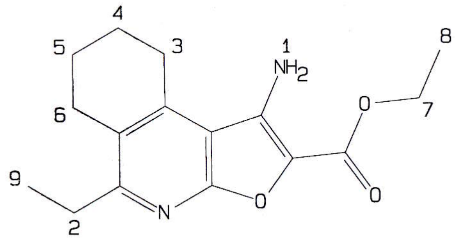

$C_{16}H_{20}N_2O_3$

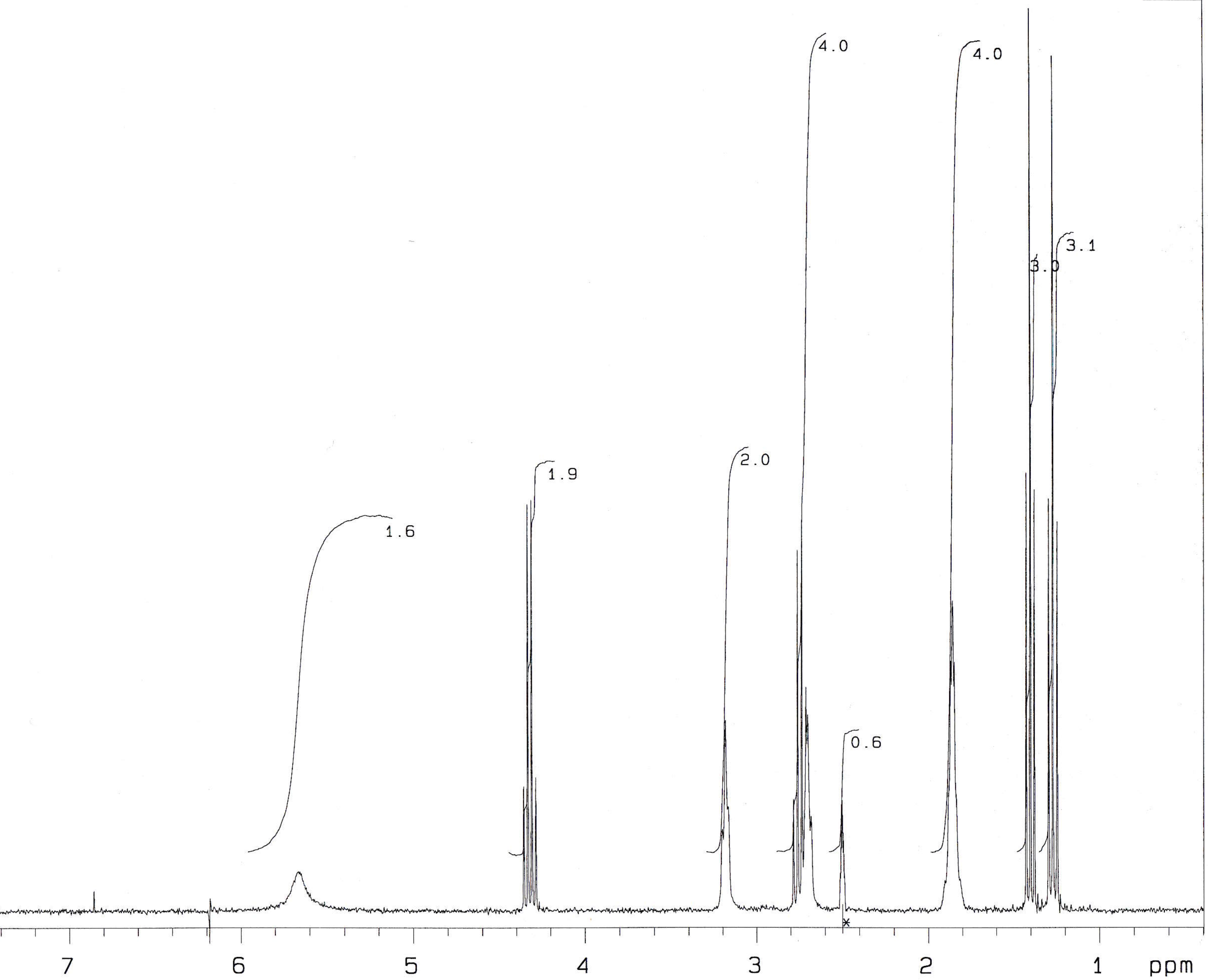

Molecular Structure Research Centre, Yerevan, Armenia, Varian Mercury-300VX

C13 75.465 MHz, nt=256, np=19998, temp=30.0 C, lb=2.0, solvent=DMSO/CCl4 1/3

Mar 31 2017

4f

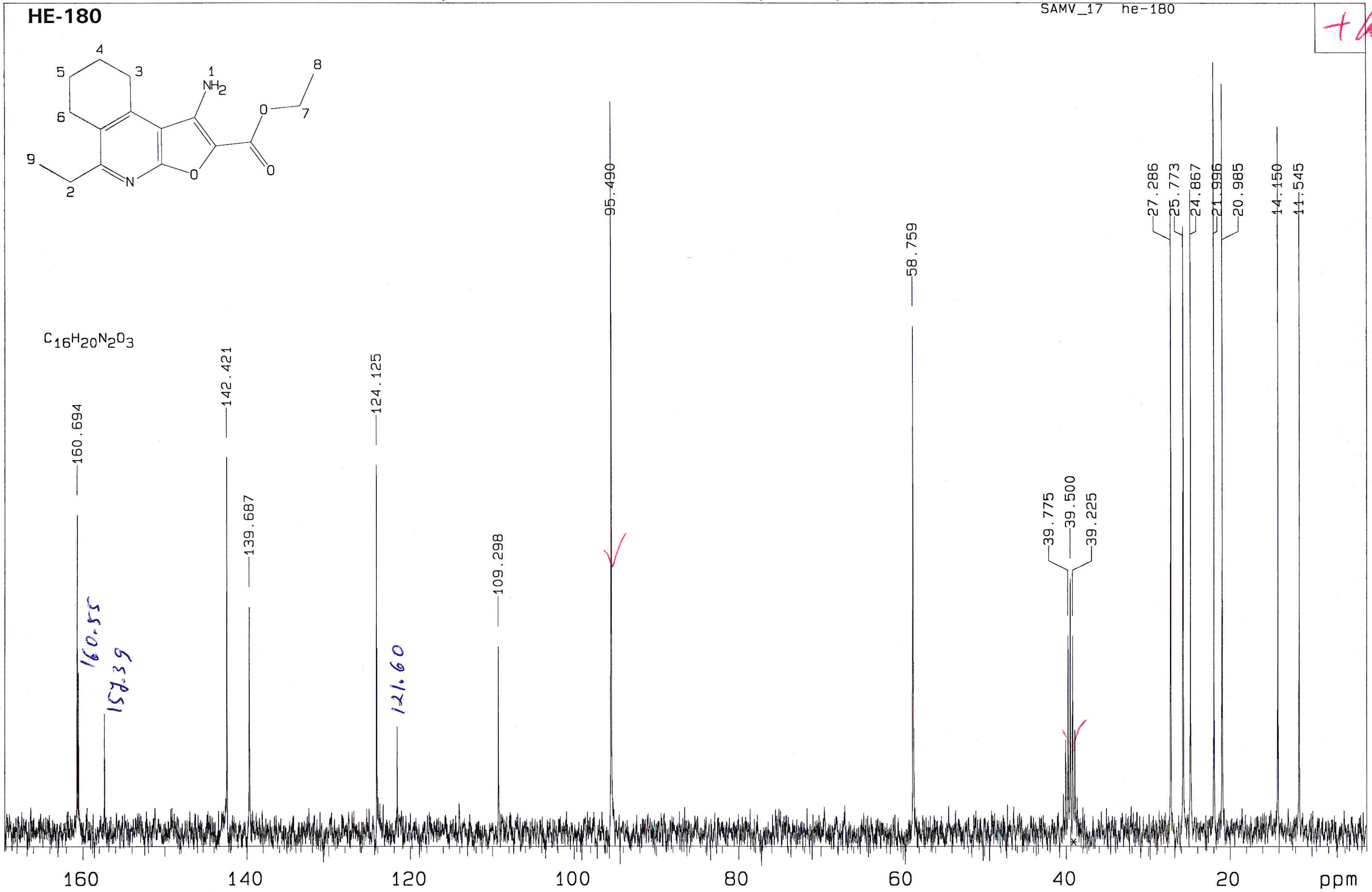

4n

Molecular Structure *Research Centre, Yerevan, Armenia, Varian Mercury-300VX*

H1 300.077 MHz, nt = 16, np = 16000, temp = 30.0 C, lb = -0.2, solvent = DMSO/CCl4 1/3

Oct 15 2015

HA-865

SAMV_15 ha-865

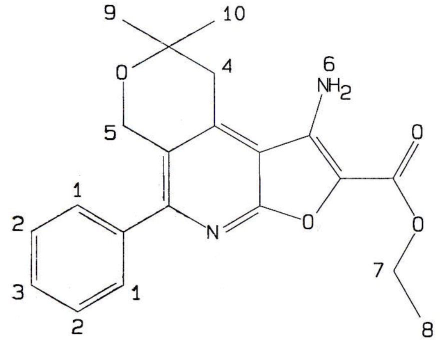

$C_{21}H_{22}N_2O_4$

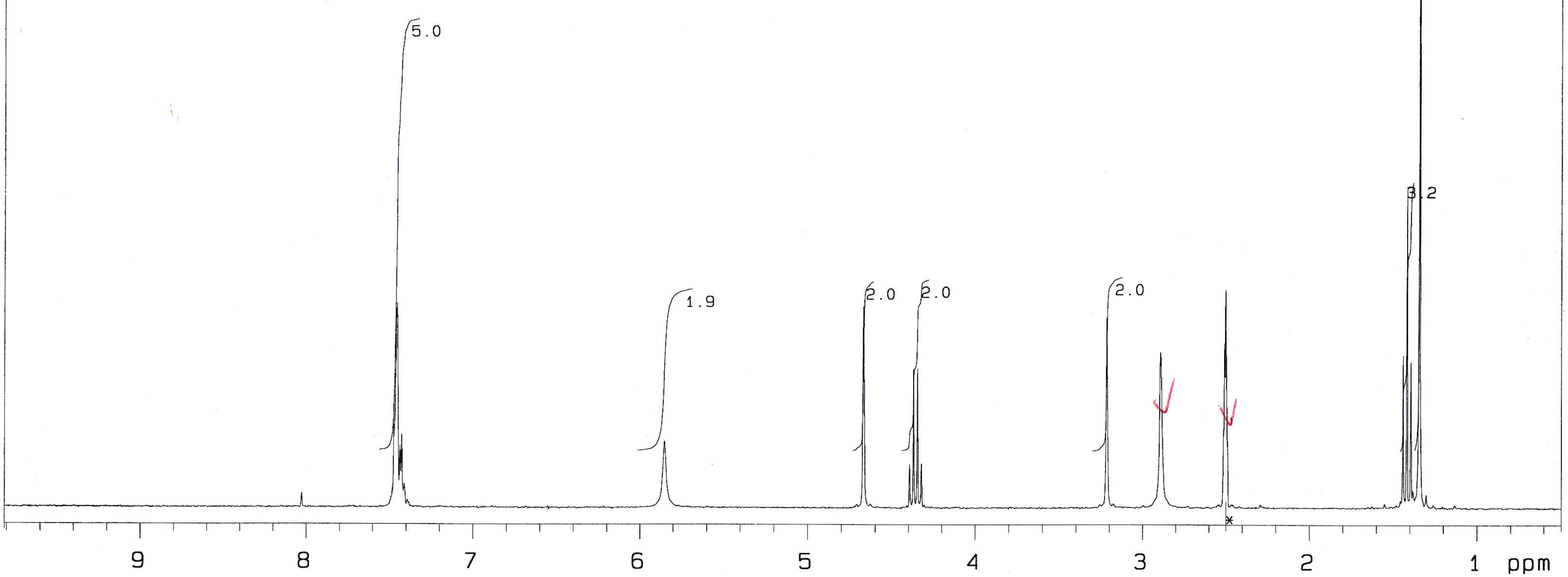

4r

Molecular Structure Research Centre, Yerevan, Armenia, Varian Mercury-300VX

C13 75.462 MHz, nt=2320, np=19998, temp=30.0 C, lb=1.0, solvent=DMSO/CCl4 1/3

Oct 15 2015

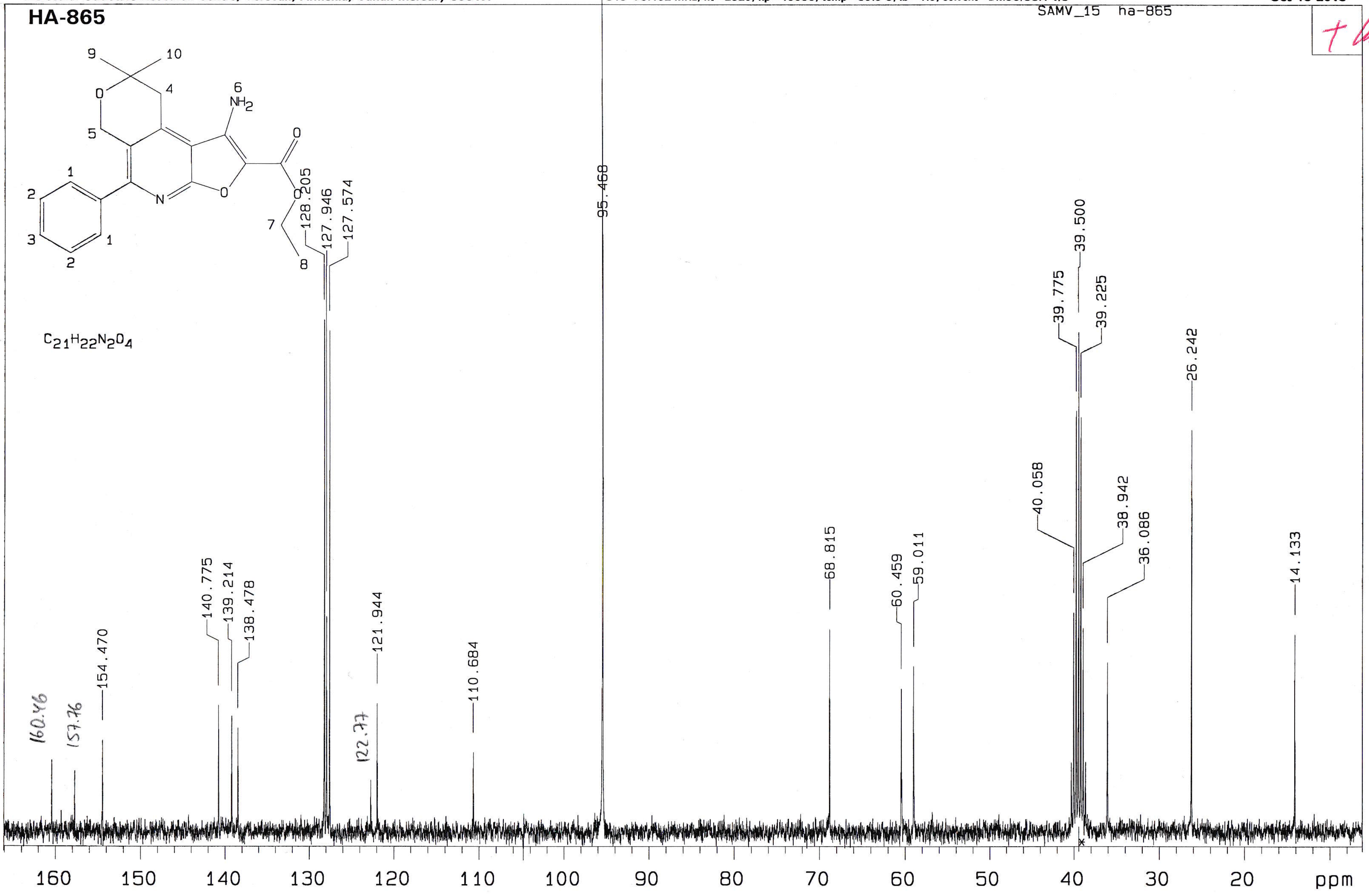

5a

Molecular Structure Research Centre, Yerevan, Armenia, Varian Mercury-300VX

H1 300.088 MHz, nt=16, np=32000, temp=30.0 C, lb=-0.2, solvent=DMSO/CCl4 1/3

Apr 12 2017

HA-972

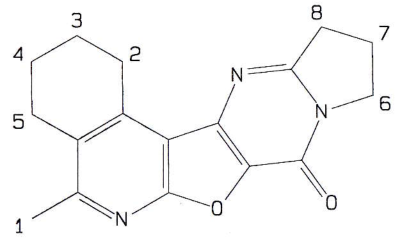

$C_{17}H_{17}N_3O_2$

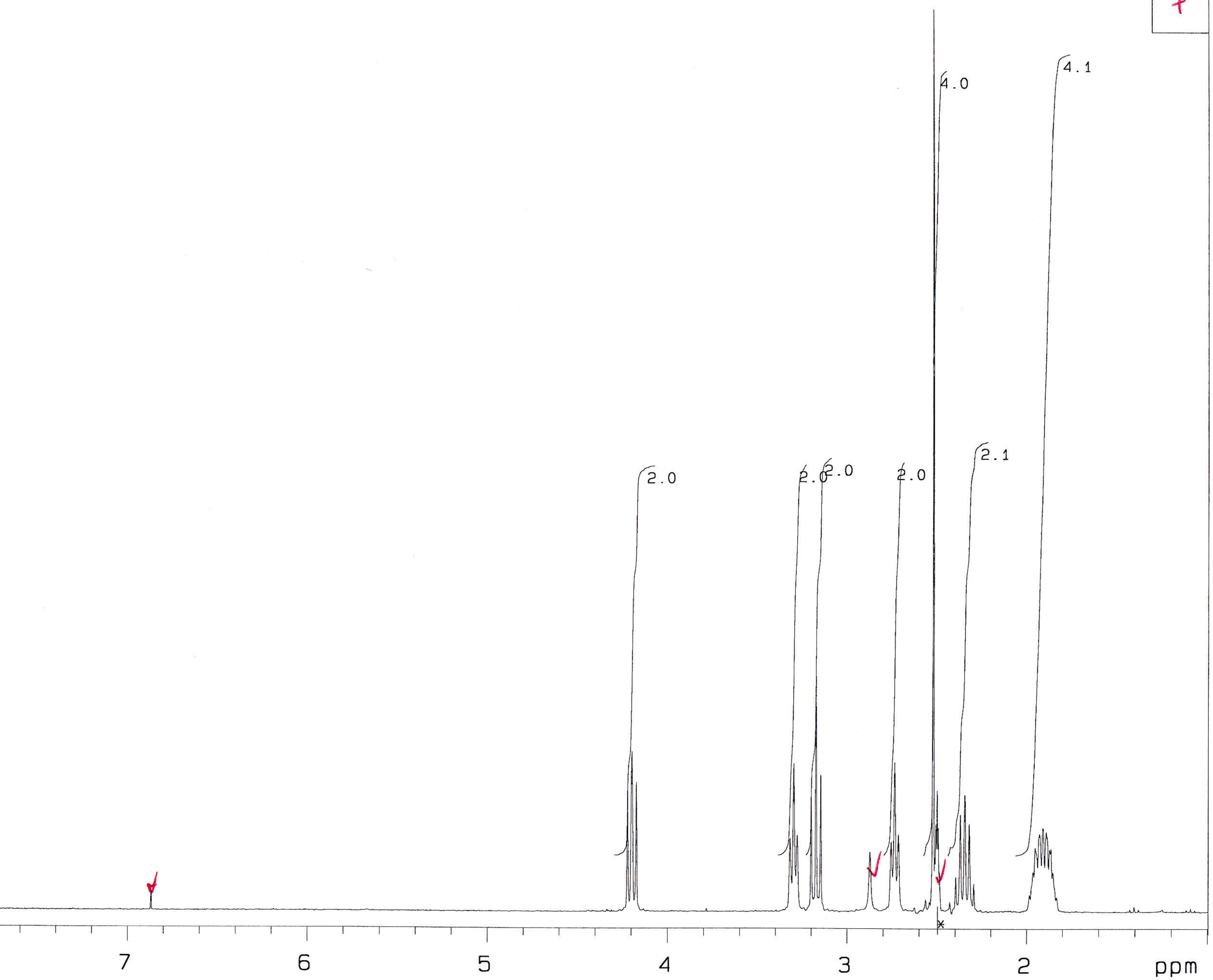

5a

Molecular Structure Research Centre, Yerevan, Armenia, Varian Mercury-300VX

C13 75.465 MHz, nt = 1936, np = 19998, temp = 30.0 C, lb = 1.0, solvent = DMSO/CCl4 1/3

Apr 12 2017

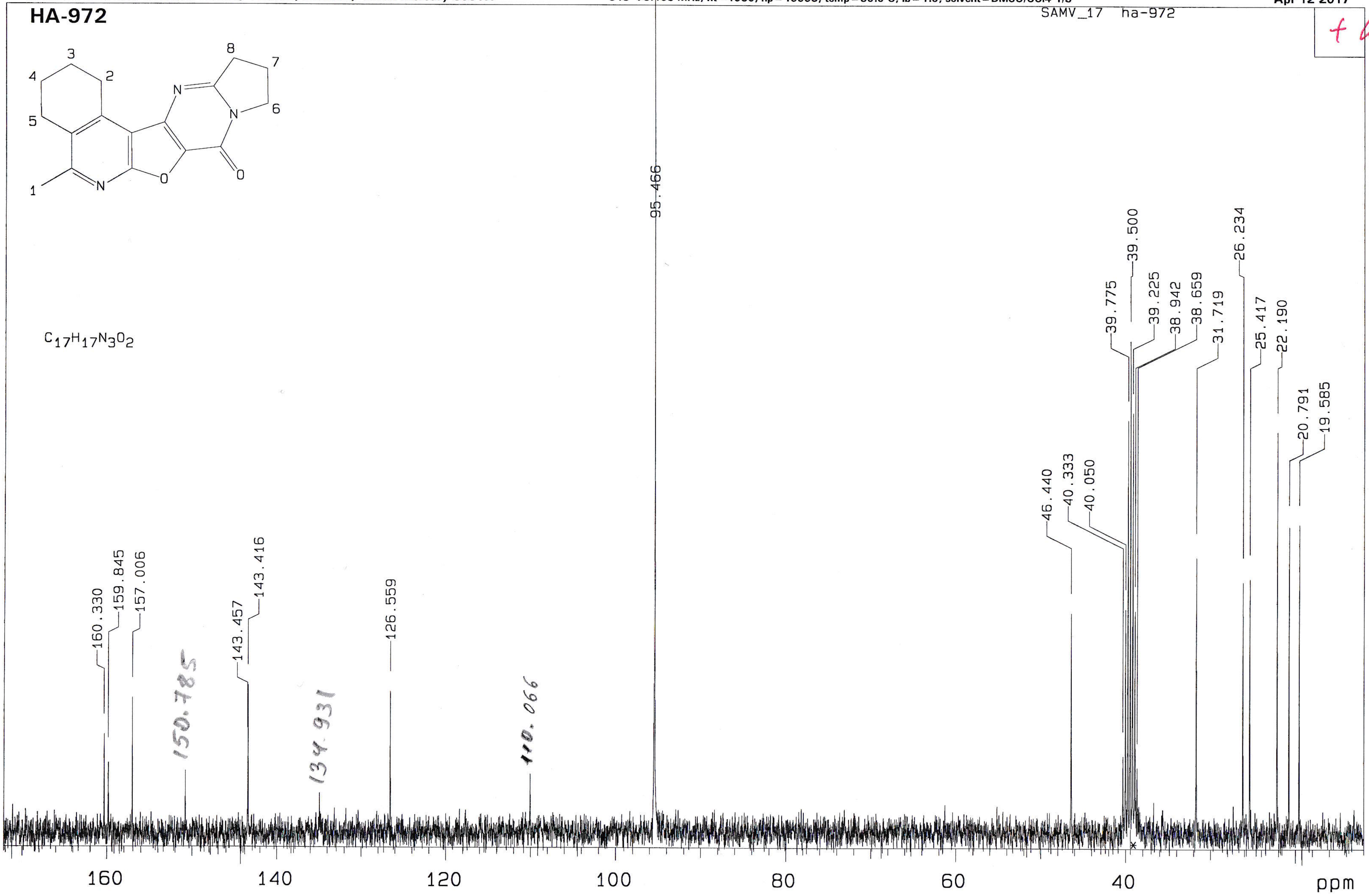

5b

Molecular Structure Research Centre, Yerevan, Armenia, Varian Mercury-300VX **H1 300.088 MHz, nt=16, np=32000, temp=30.0 C, lb=-0.2, solvent=DMSO/CCl4 1/3** **Apr 19 2017**

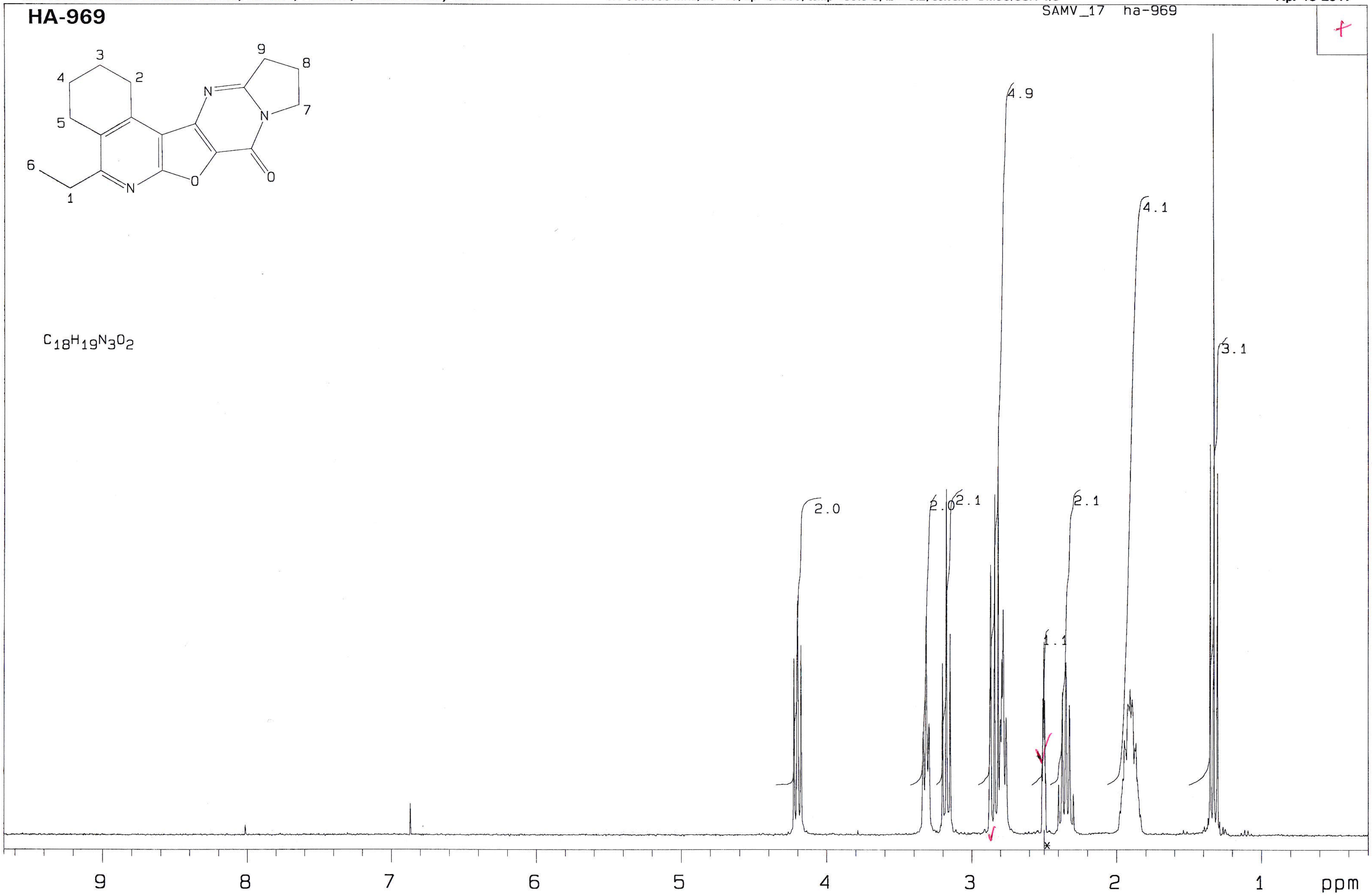

5b

Molecular Structure Research Centre, Yerevan, Armenia, Varian Mercury-300VX

C13 75.465 MHz, nt=544, np=19998, temp=30.0 C, lb=1.0, solvent=DMSO/CCl4 1/3

Apr 19 2017

HA-969

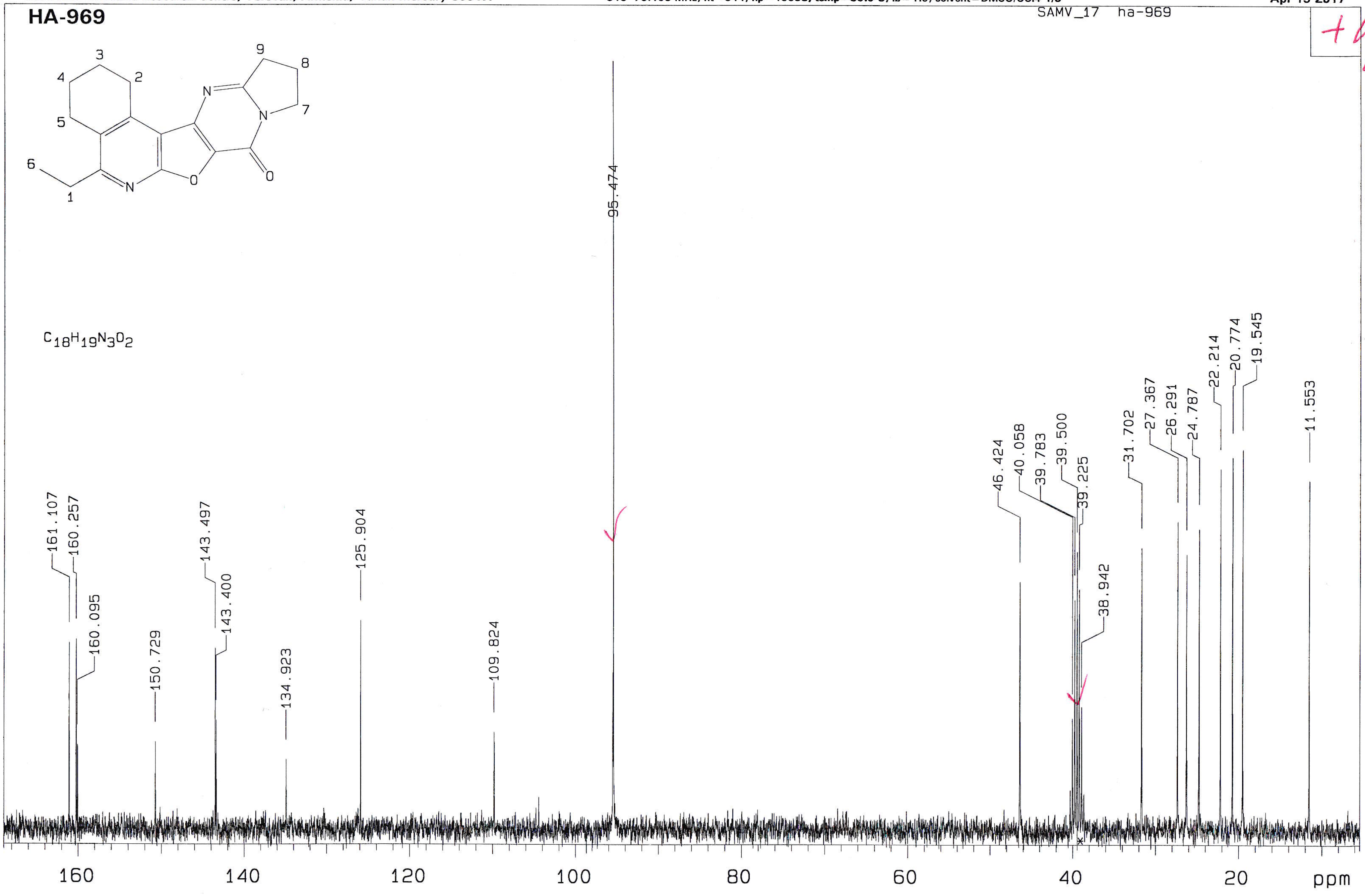

5c

Molecular Structure Research Centre, Yerevan, Armenia, Varian Mercury-300VX

H1 300.088 MHz, nt=16, np=32000, temp=30.0 C, lb=-0.2, solvent=DMSO/CCl4 1/3

Feb 16 2017

SAMV_17 ha-954-1

HA-954-1

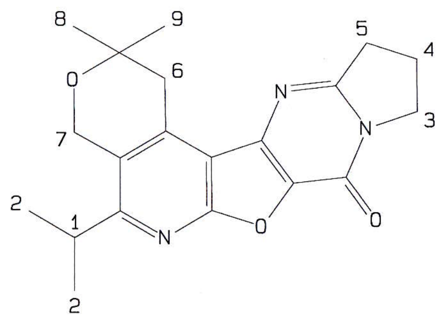

$C_{20}H_{23}N_3O_3$

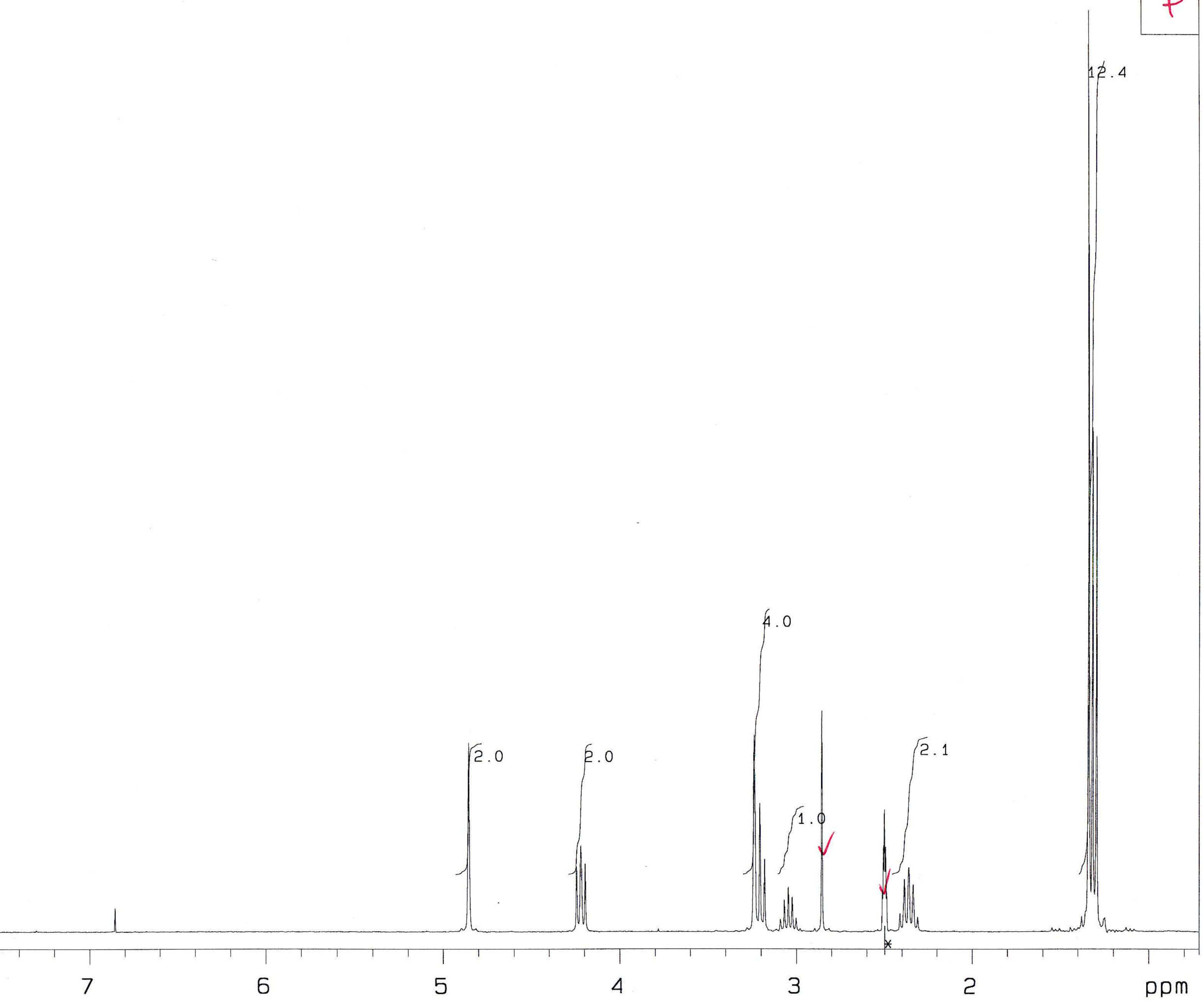

5c

Molecular Structure Research Centre, Yerevan, Armenia, Varian Mercury-300VX

C13 75.465 MHz, nt=848, np=19998, temp=30.0 C, lb=1.0, solvent=DMSO/CCl4 1/3

Feb 15 2017

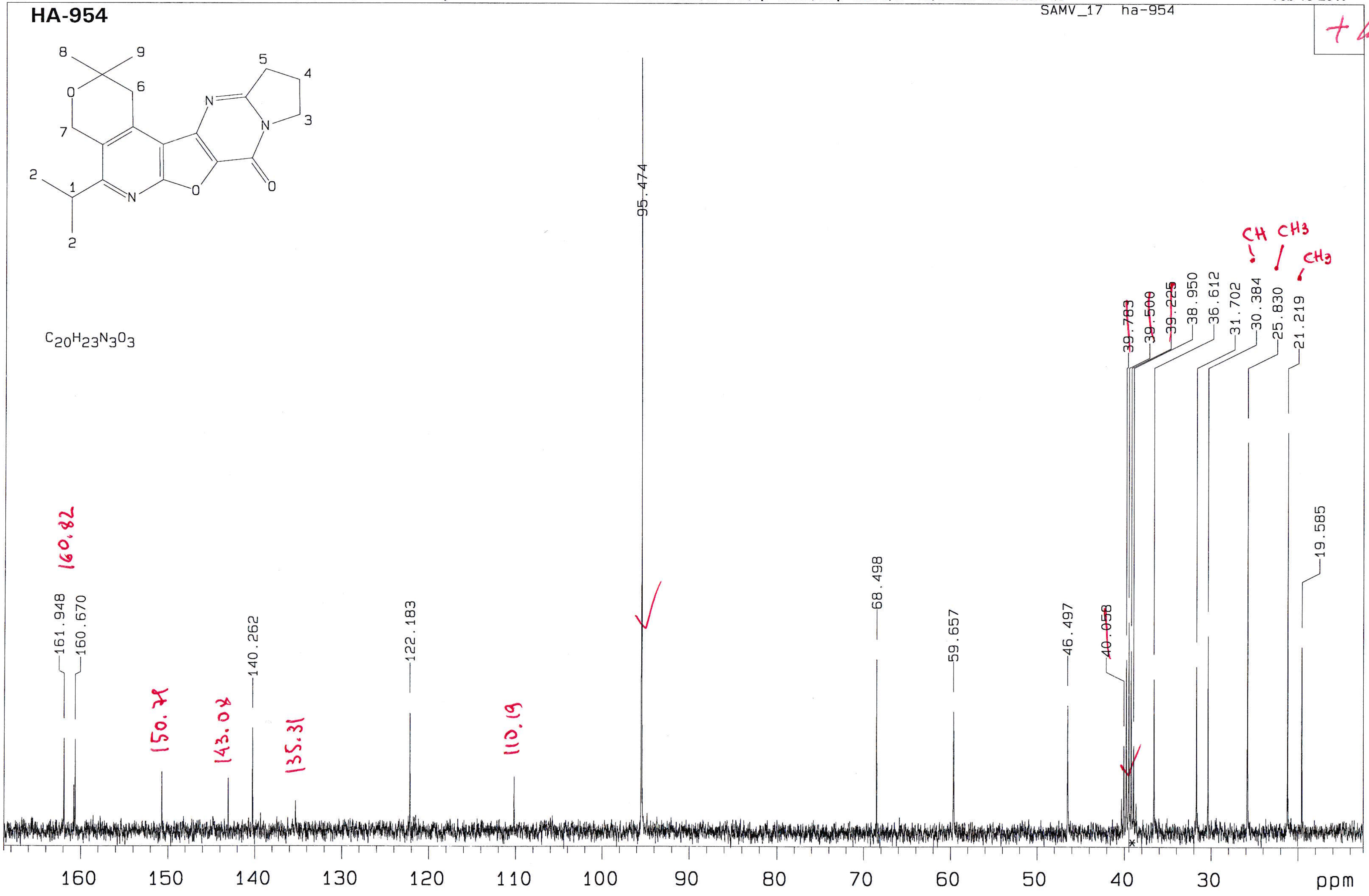

5a

Molecular Structure Research Centre, Yerevan, Armenia, Varian Mercury-300VX

H1 300.088 MHz, nt = 16, np = 32000, temp = 30.0 C, lb = -0.2, solvent = DMSO/CCl4 1/3

Feb 24 2017

HA-959

SAMV_17 ha-959

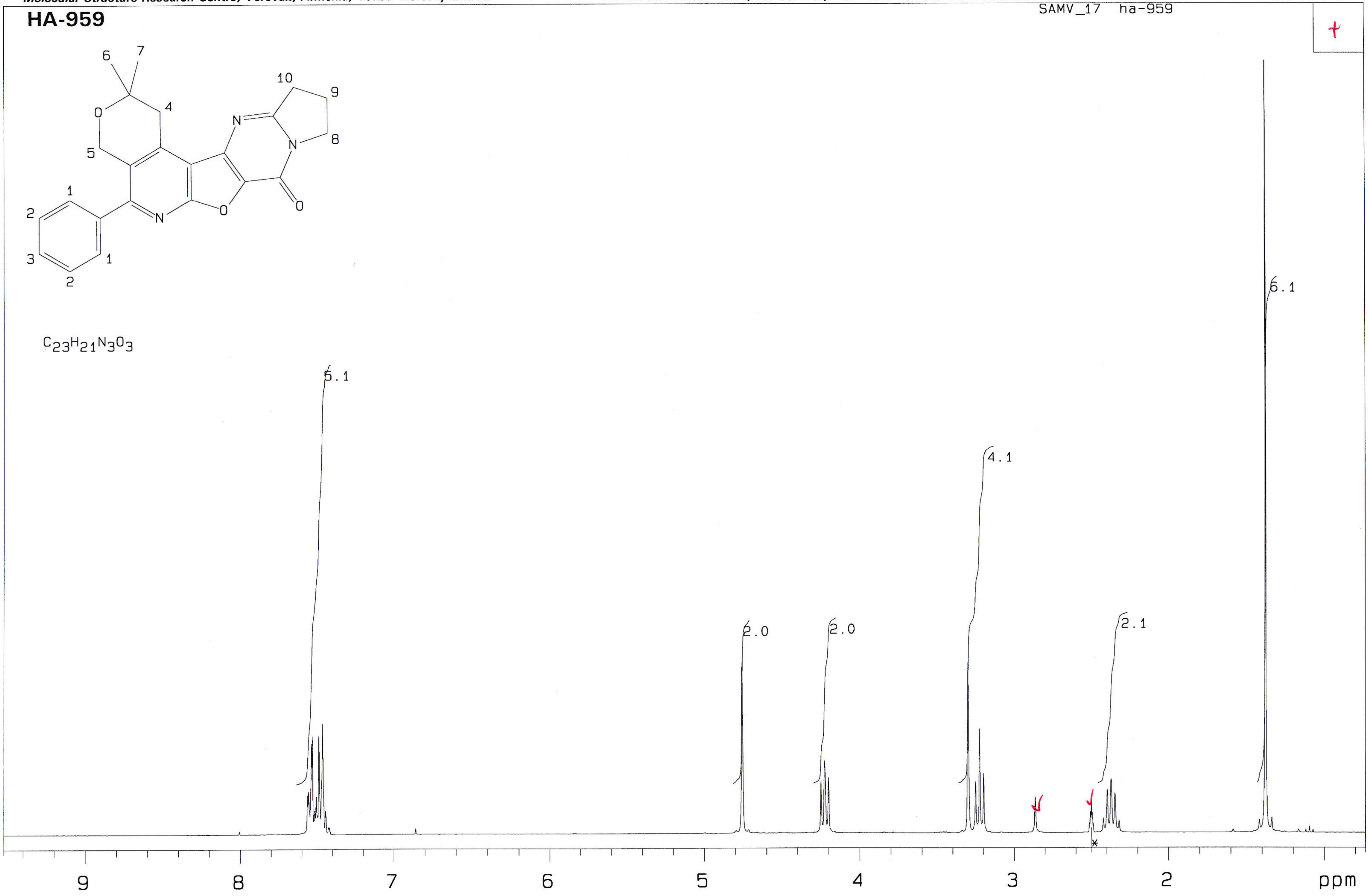

5d

Molecular Structure Research Centre, Yerevan, Armenia, Varian Mercury-300VX

C13 75.465 MHz, nt = 384, np = 19998, temp = 30.0 C, lb = 1.0, solvent = DMSO/CCl4 1/3

Feb 24 2017

HA-959

SAMV_17 ha-959

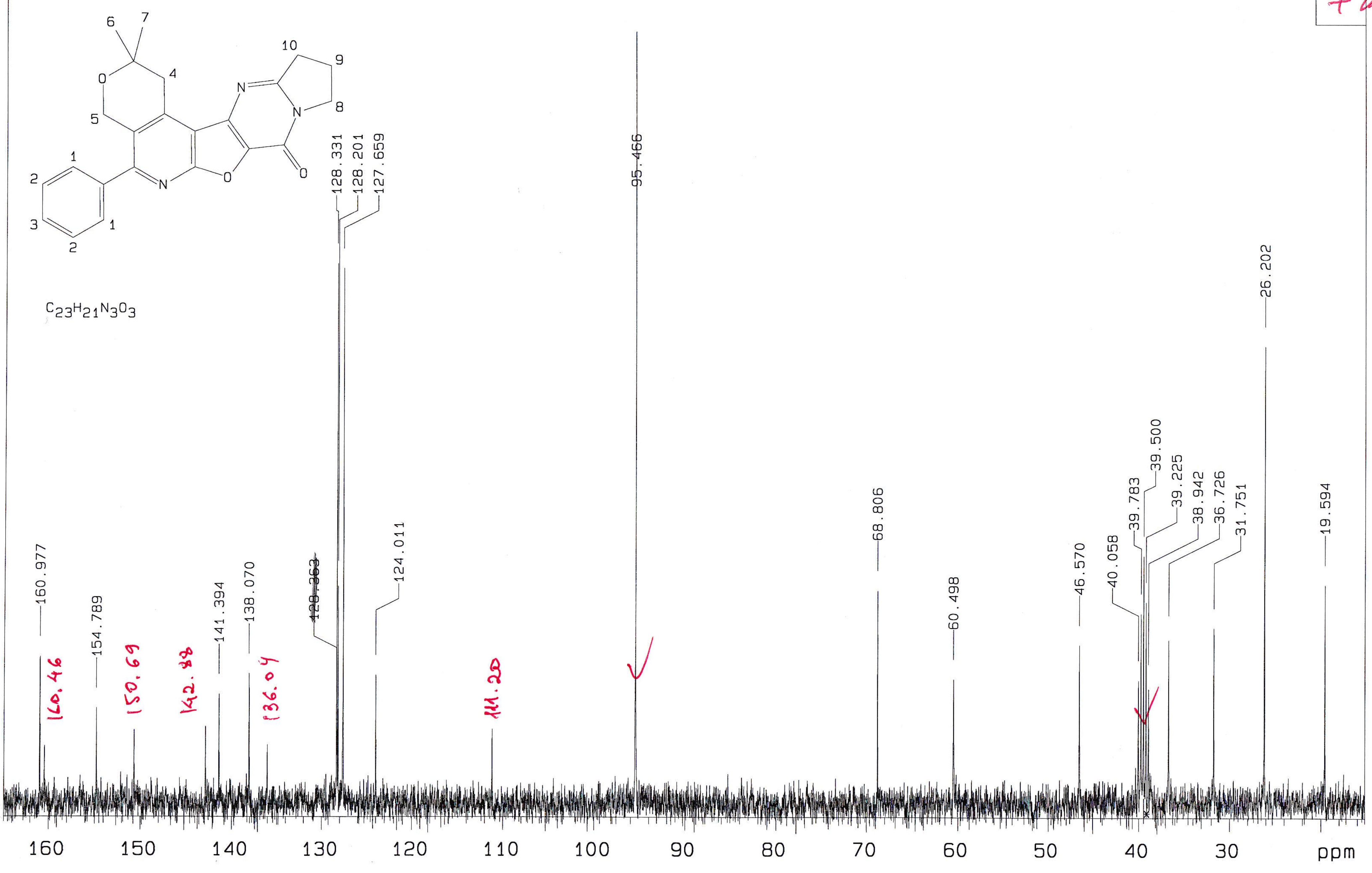

6a

Molecular Structure Research Centre, Yerevan, Armenia, Varian Mercury-300VX

H1 300.088 MHz, nt=16, np=32000, temp=30.0 C, lb=-0.2, solvent=DMSO/CCl4 1/3

Feb 2 2017

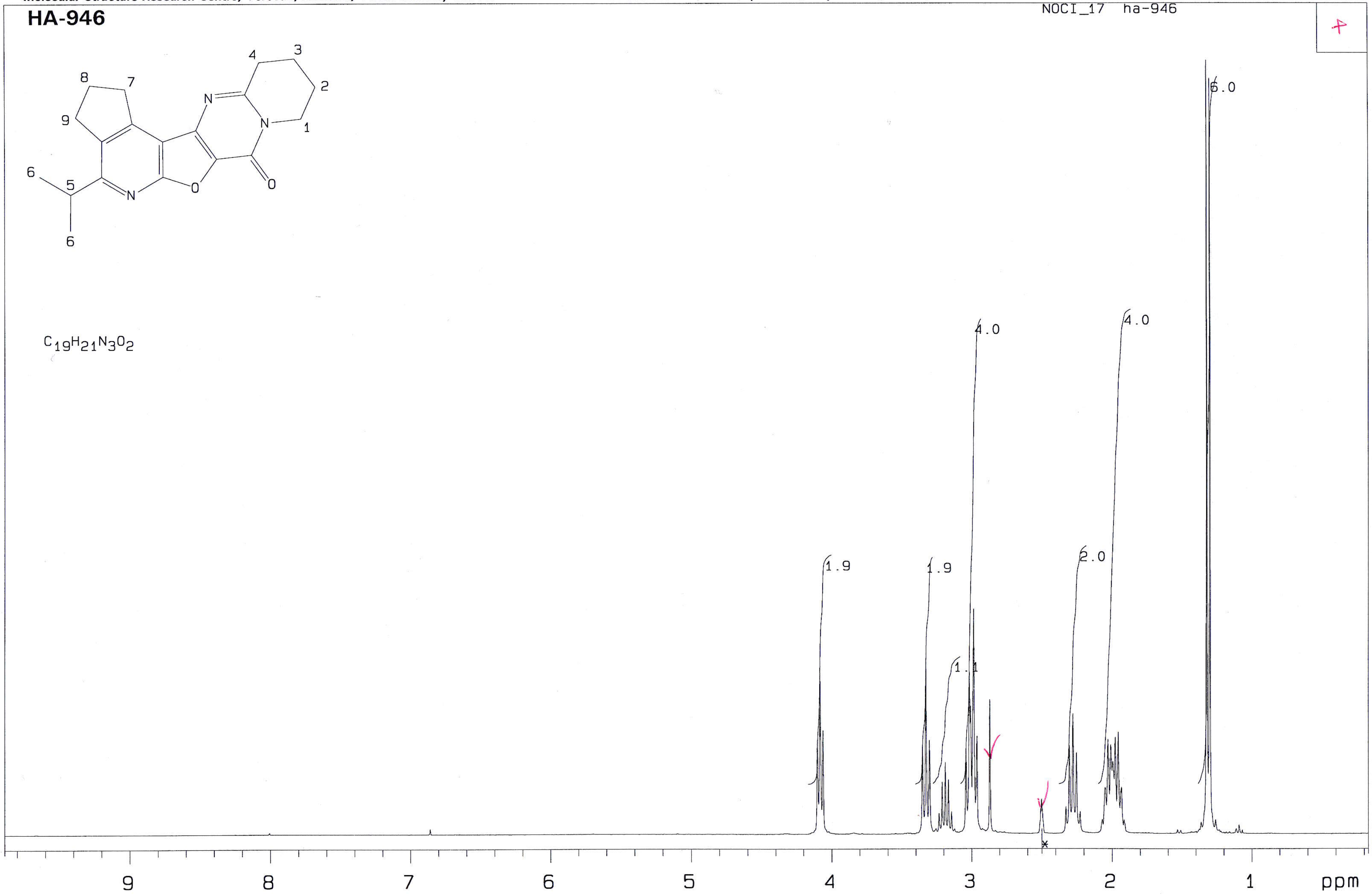

6a

Molecular Structure Research Centre, Yerevan, Armenia, Varian Mercury-300VX

C13 75.465 MHz, nt = 272, np = 19998, temp = 30.0 C, lb = 1.0, solvent = DMSO/CCl4 1/3

Feb 2 2017

NOCI_17 ha-946

HA-946

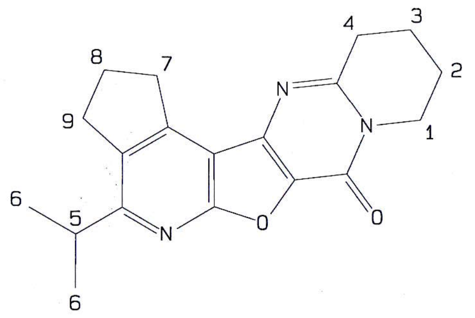

$C_{19}H_{21}N_3O_2$

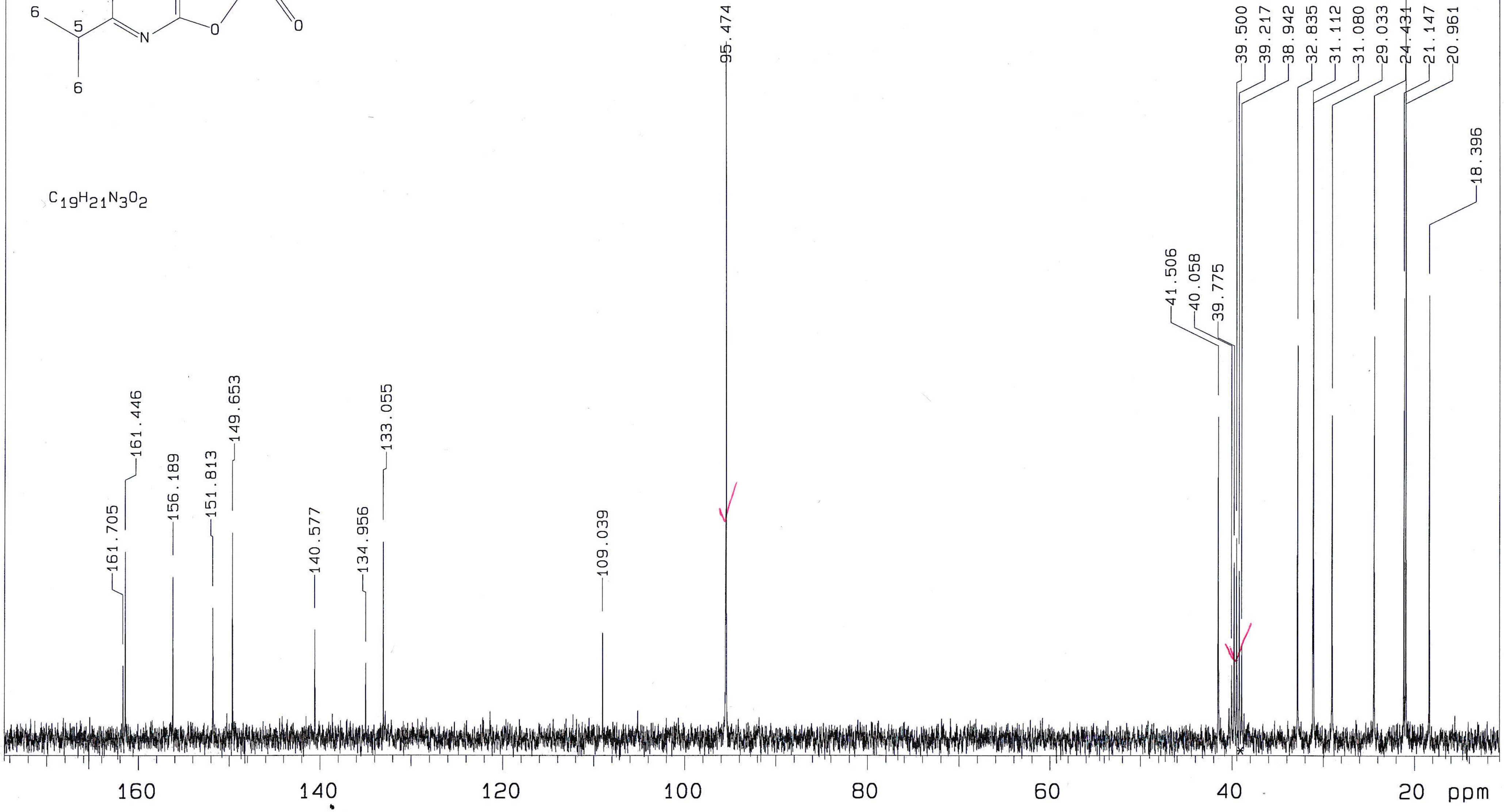

Molecular Structure Research Centre, Yerevan, Armenia, Varian Mercury-300VX

H1 300.088 MHz, nt=16, np=32000, temp=30.0 C, lb=-0.2, solvent=DMSO/CCl4 1/3

Feb 14 2017

6b

SAMV_17 ha-952

HA-952

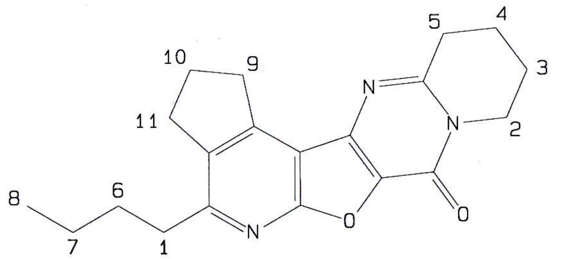

$C_{20}H_{23}N_3O_2$

0.99 (3H, т, J=7.3, CH$_3$)
1.37–1.50 (2H, m, CH$_2$CH$_3$)
1.71–1.81 (2H, m, CH$_2$C$_2$H$_5$)
1.91–2.08 (4H, m, 2·CH$_2$)
2.23–2.33 (2H, m, CH$_2$)
2.78–2.83 (2H, m, CH$_2$C$_3$H$_7$)
2.96–3.02 (4H, m, 2·CH$_2$)
3.35 (2H, т, J=7.6, CH$_2$)
4.09 (2H, т, J=6.1, CH$_2$)

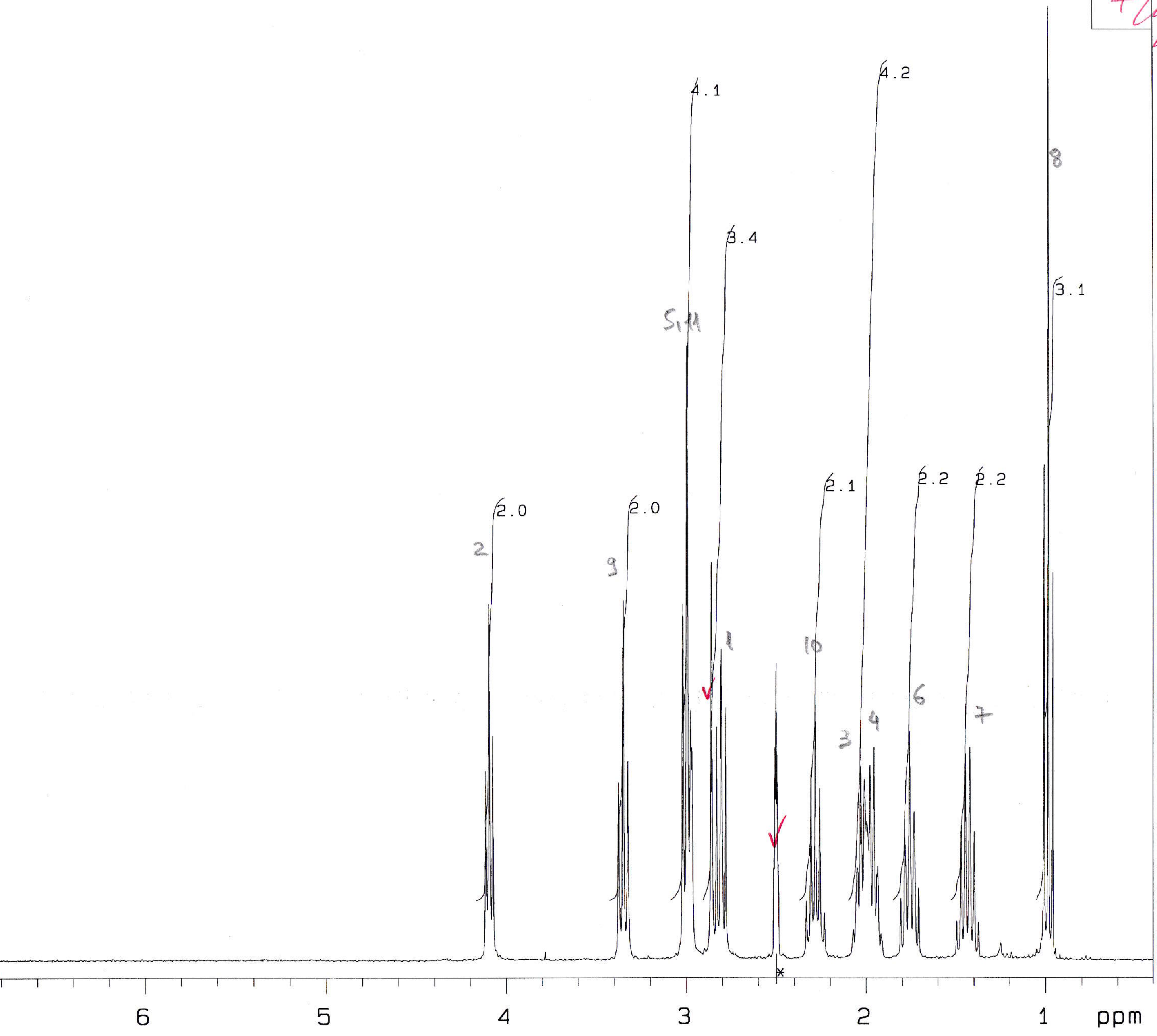

6b

Molecular Structure Research Centre, Yerevan, Armenia, Varian Mercury-300VX C13 75.465 MHz, nt = 672, np = 19998, temp = 30.0 C, lb = 1.0, solvent = DMSO/CCl4 1/3 **Feb 14 2017**

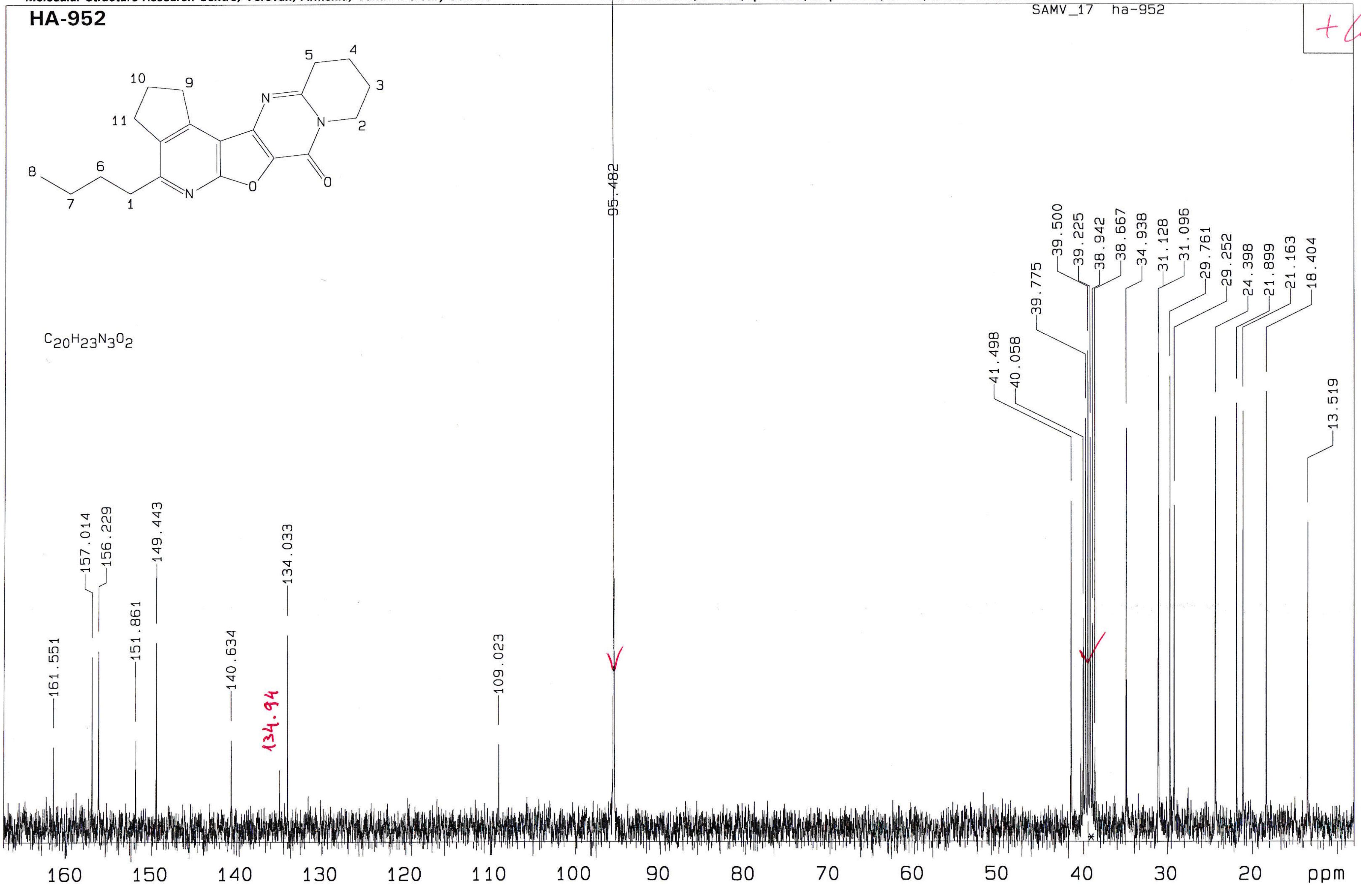

6c

Molecular Structure Research Centre, Yerevan, Armenia, Varian Mercury-300VX H1 300.088 MHz, nt = 16, np = 32000, temp = 30.0 C, lb = -0.2, solvent = DMSO/CCl4 1/3 **Feb 24 2017**

HA-958

SAMV_17 ha-958

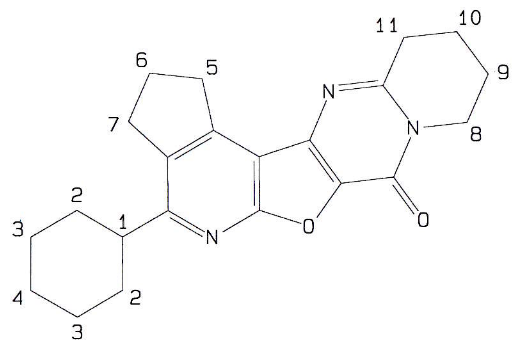

$C_{22}H_{25}N_3O_2$

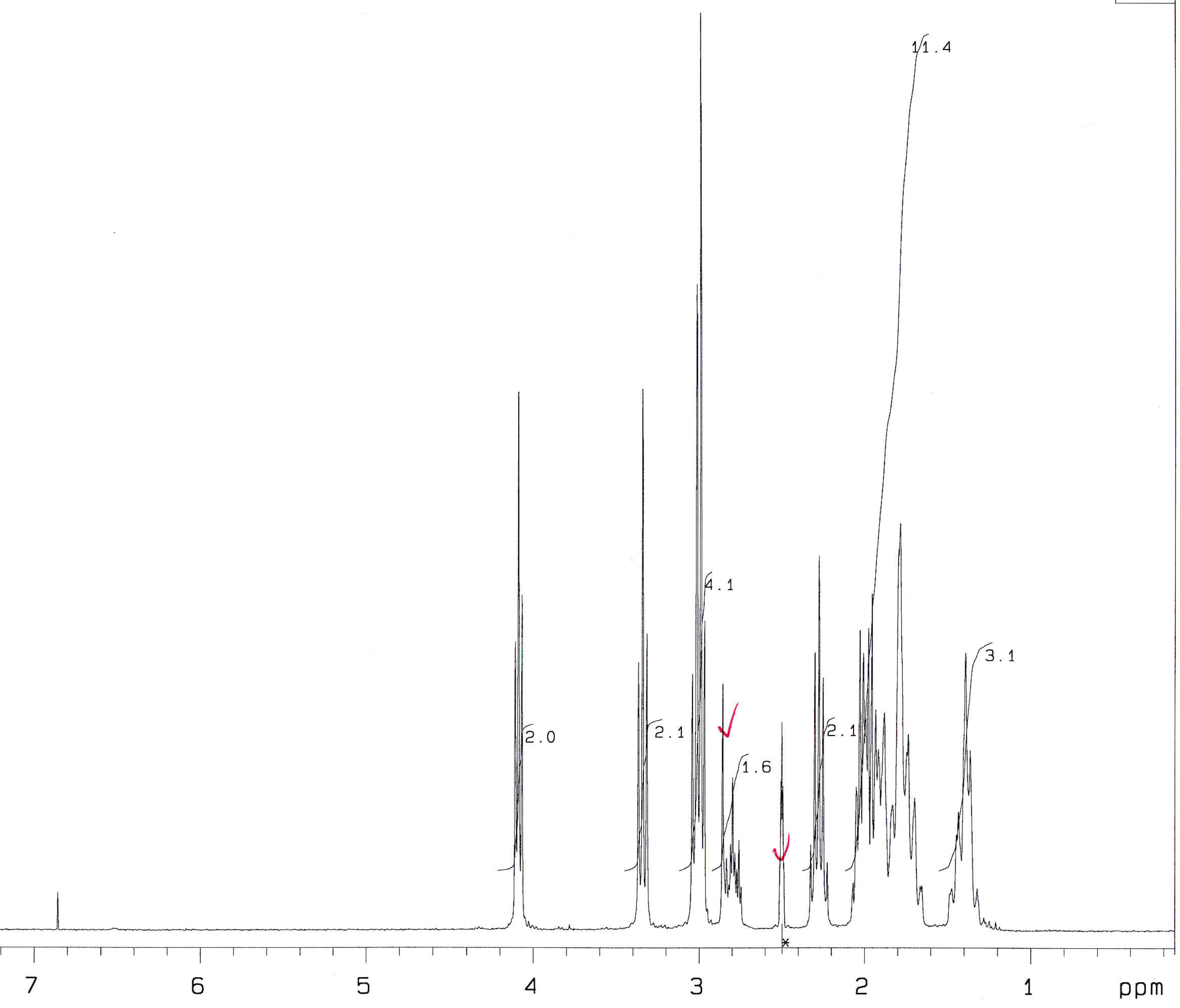

6c

Molecular Structure Research Centre, Yerevan, Armenia, Varian Mercury-300VX

C13 75.465 MHz, nt = 256, np = 19998, temp = 30.0 C, lb = 1.0, solvent = DMSO/CCl4 1/3

Feb 24 2017

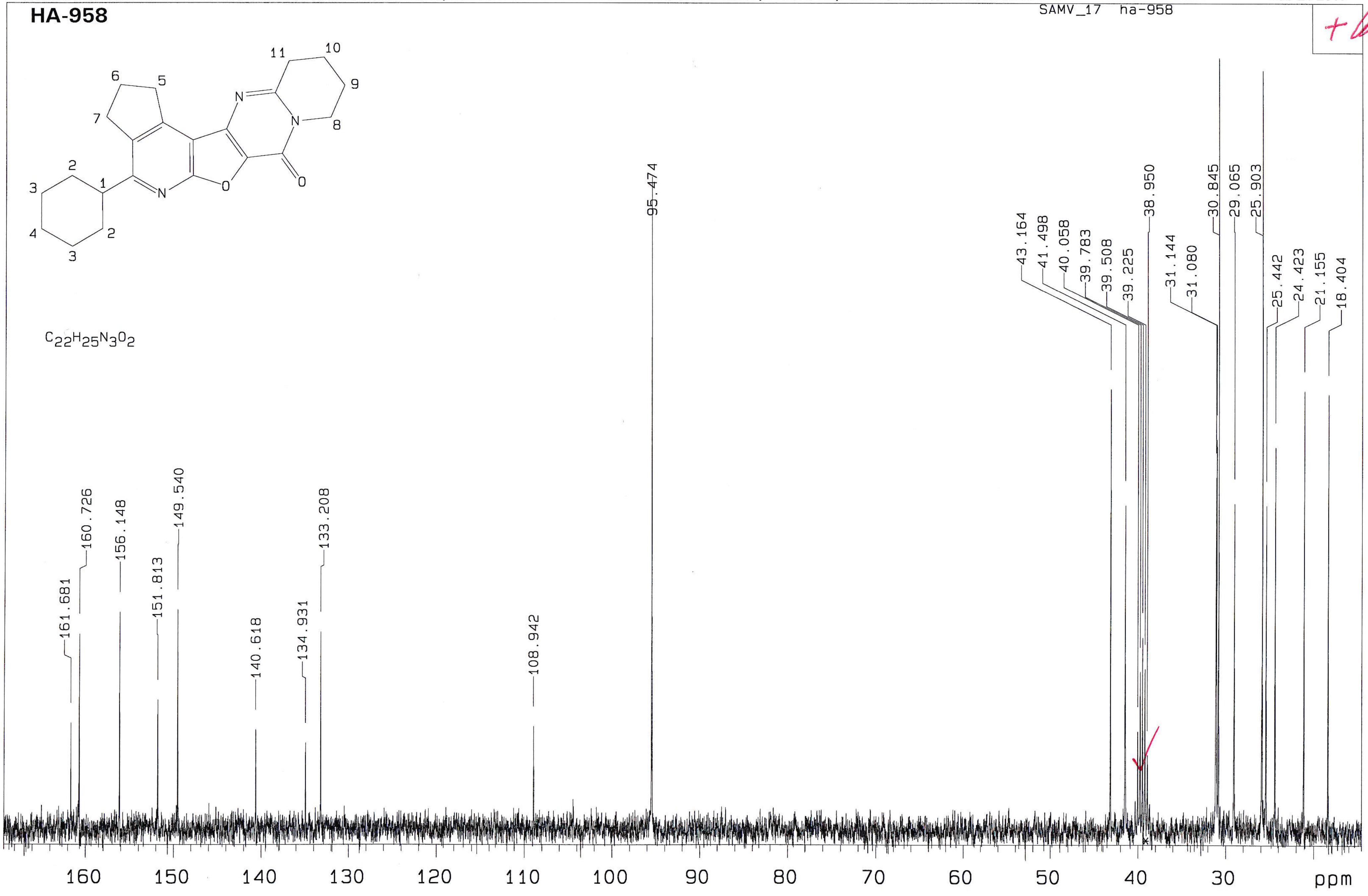

Molecular Structure Research Centre, Yerevan, Armenia, Varian Mercury-300VX

H1 300.088 MHz, nt = 16, np = 32000, temp = 30.0 C, lb = -0.2, solvent = DMSO/CCl4 1/3

Apr 6 2017

HA-970-1

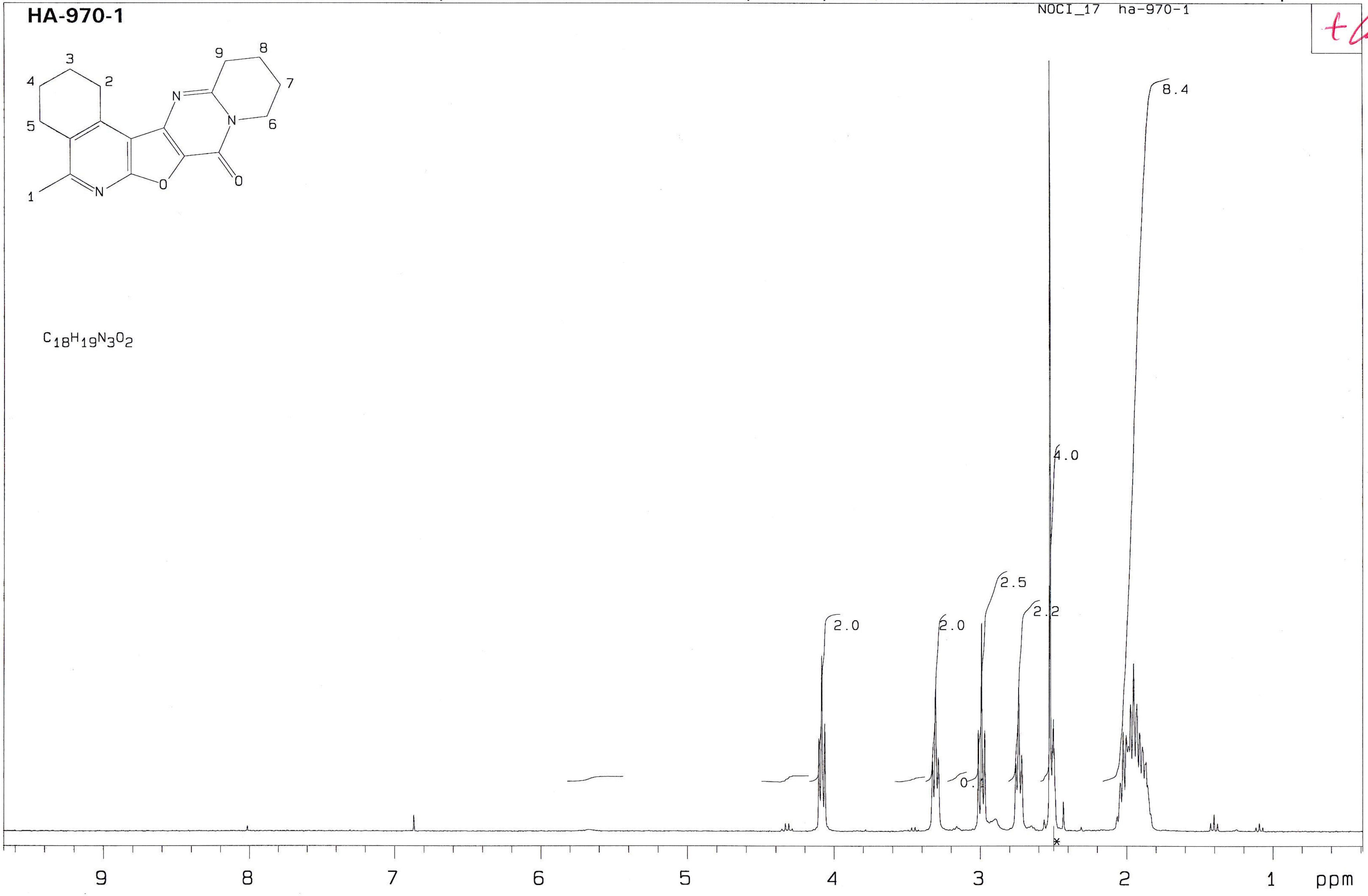

6d

Molecular Structure Research Centre, Yerevan, Armenia, Varian Mercury-300VX

C13 75.465 MHz, nt=4208, np=19998, temp=30.0 C, lb=1.0, solvent=DMSO/CCl4 1/3

Apr 6 2017

NOCI_17 ha-970-1

HA-970-1

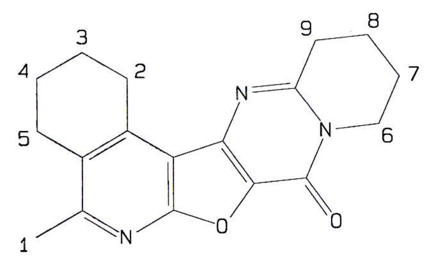

$C_{18}H_{19}N_3O_2$

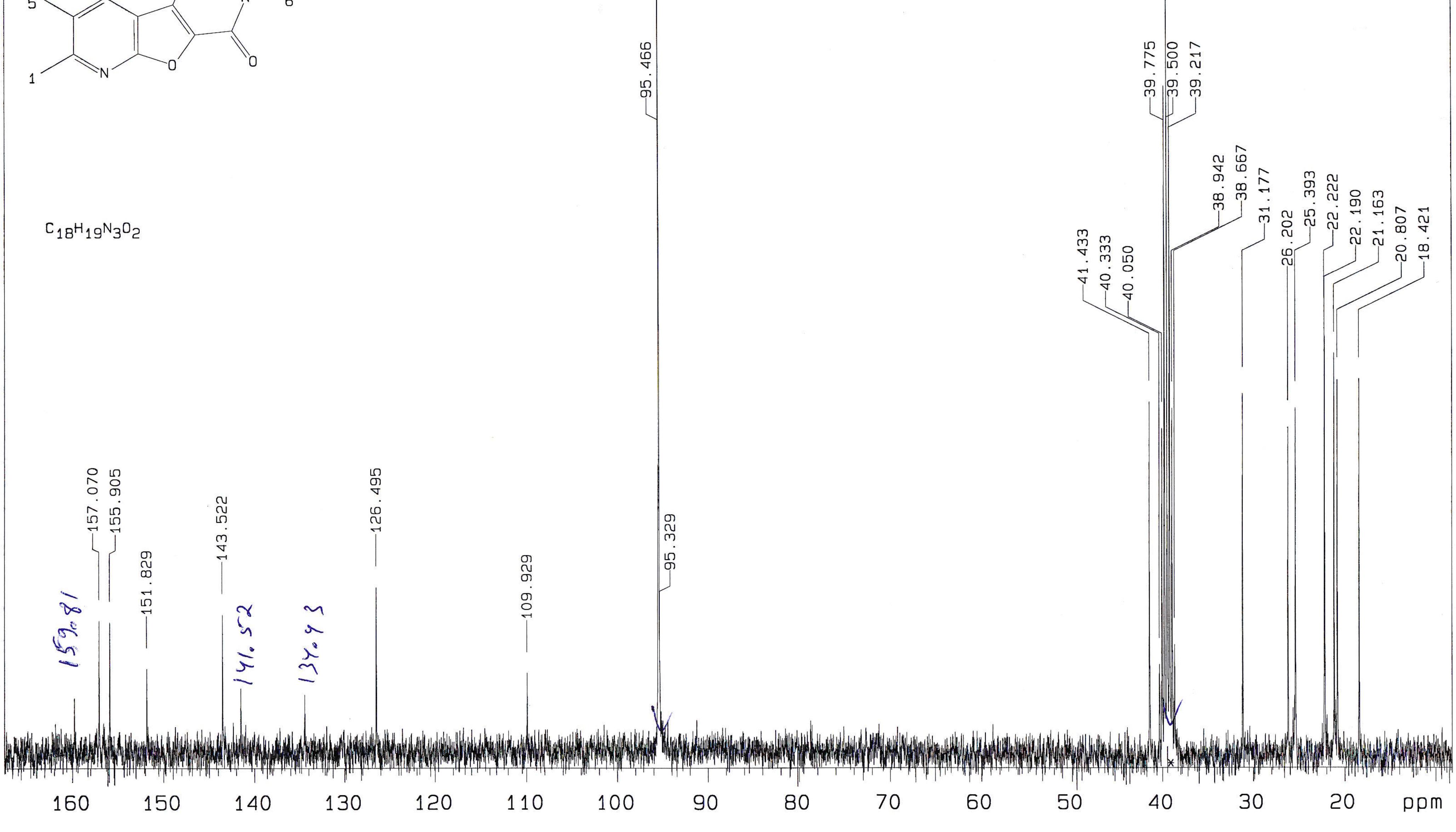

6e

Molecular Structure Research Centre, Yerevan, Armenia, Varian Mercury-300VX H1 300.088 MHz, nt = 16, np = 16000, temp = 30.0 C, lb = -0.2, solvent = DMSO/CCl4 1/3 Apr 5 2017

HA-967

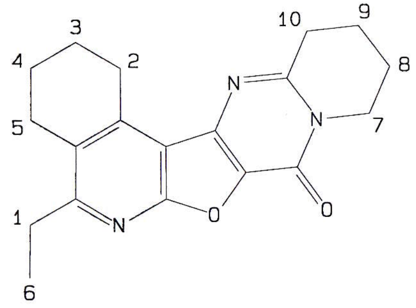

$C_{19}H_{21}N_3O_2$

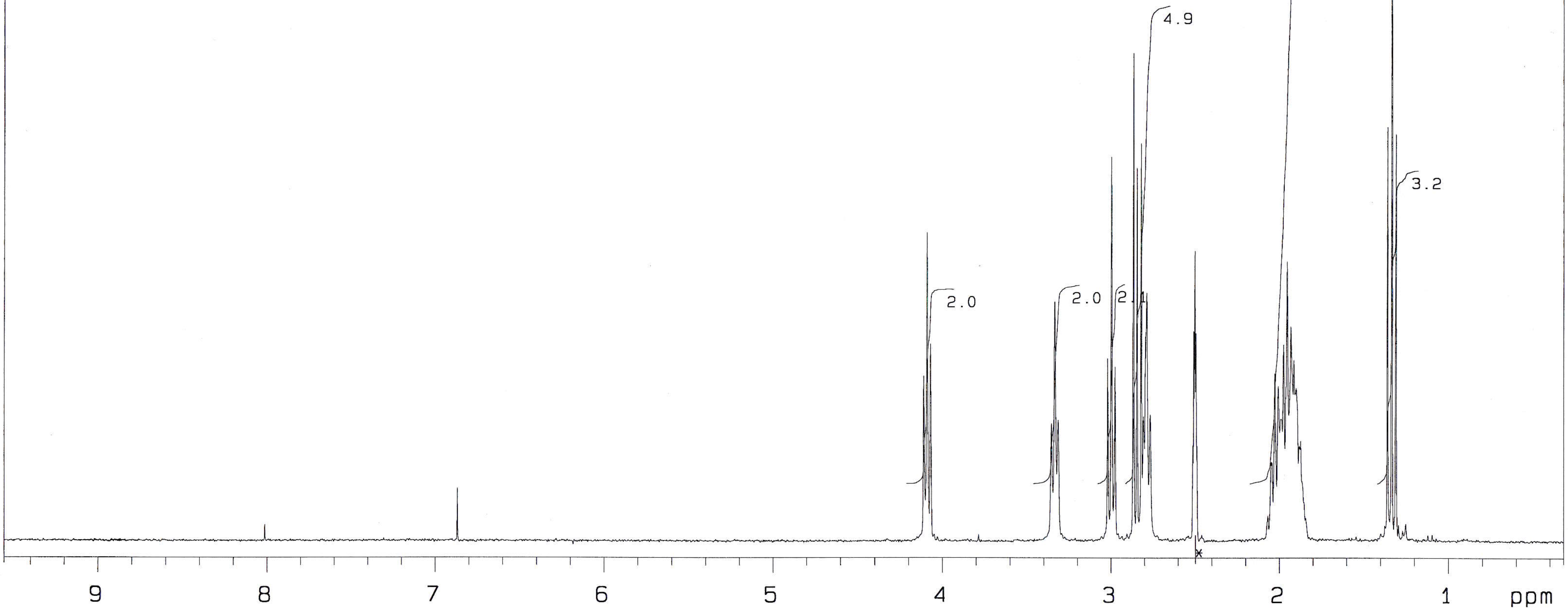

Molecular Structure Research Centre, Yerevan, Armenia, Varian Mercury-300VX

C13 75.465 MHz, nt = 1760, np = 19998, temp = 30.0 C, lb = 1.0, solvent = DMSO/CCl4 1/3

Apr 5 2017

6e

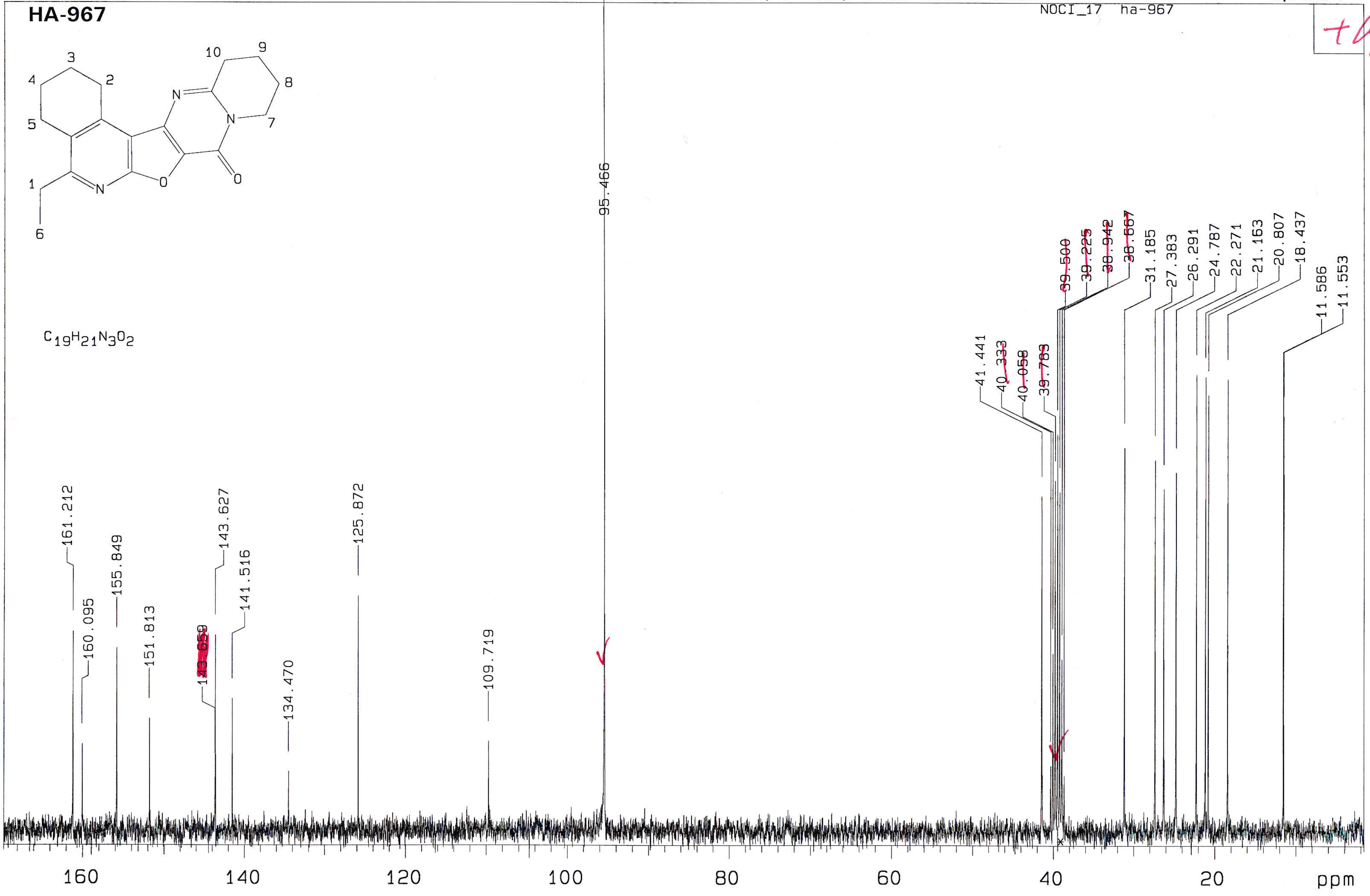

6f

Molecular Structure Research Centre, Yerevan, Armenia, Varian Mercury-300VX   H1 300.088 MHz, nt = 16, np = 32000, temp = 30.0 C, lb = -0.2, solvent = DMSO/CCl4 1/3   Mar 28 2017

HA-950

SAMV_17 ha-950

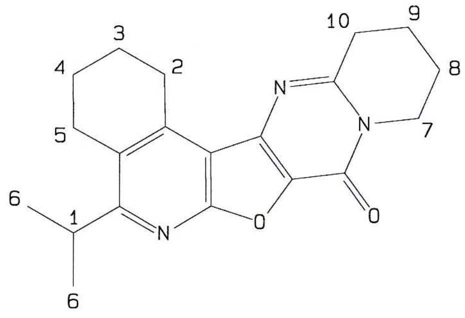

$C_{20}H_{23}N_3O_2$

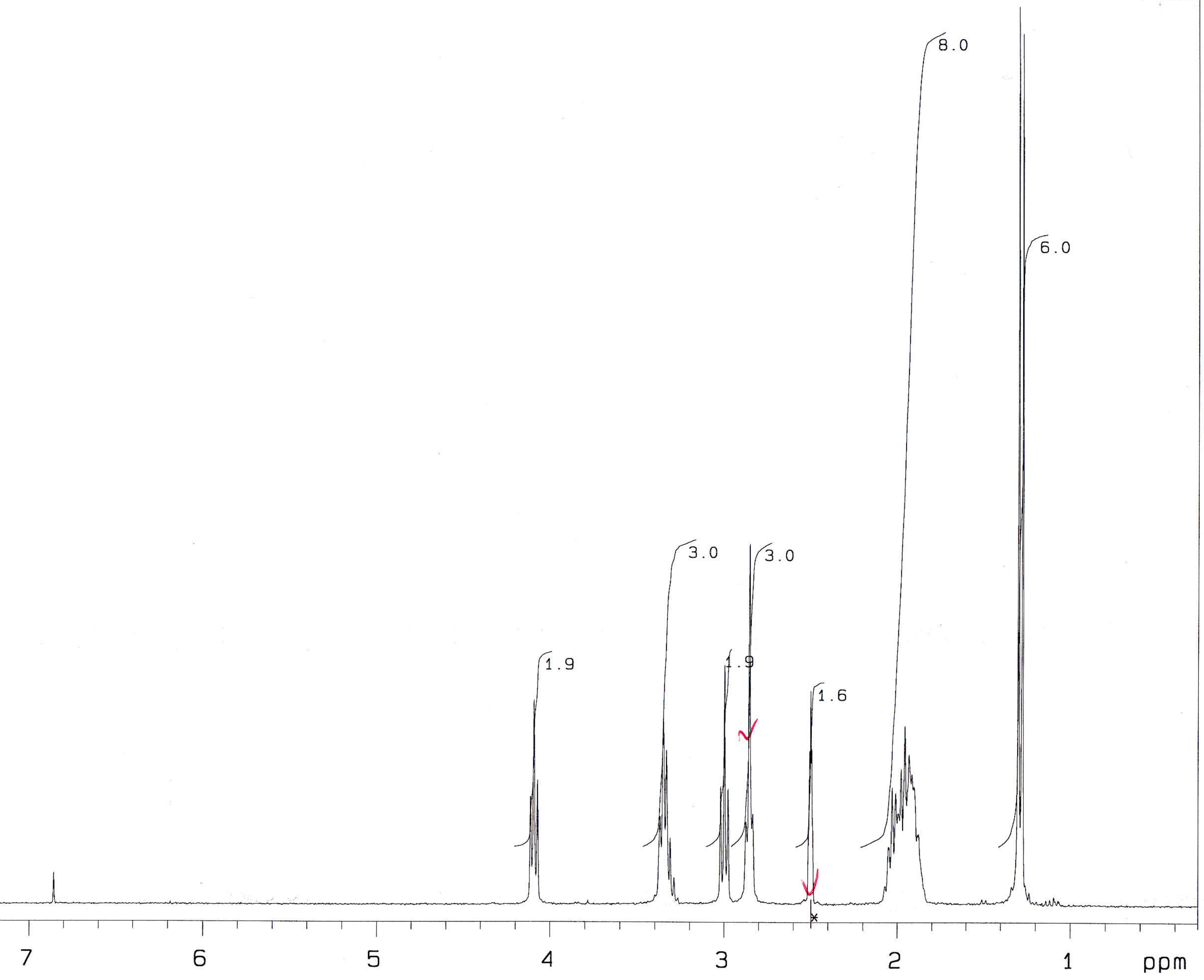

6f

Molecular Structure Research Centre, Yerevan, Armenia, Varian Mercury-300VX

C13 75.465 MHz, nt = 4640, np = 19998, temp = 30.0 C, lb = 1.0, solvent = DMSO/CCl4 1/3

Mar 28 2017

HA-950

SAMV_17 ha-950

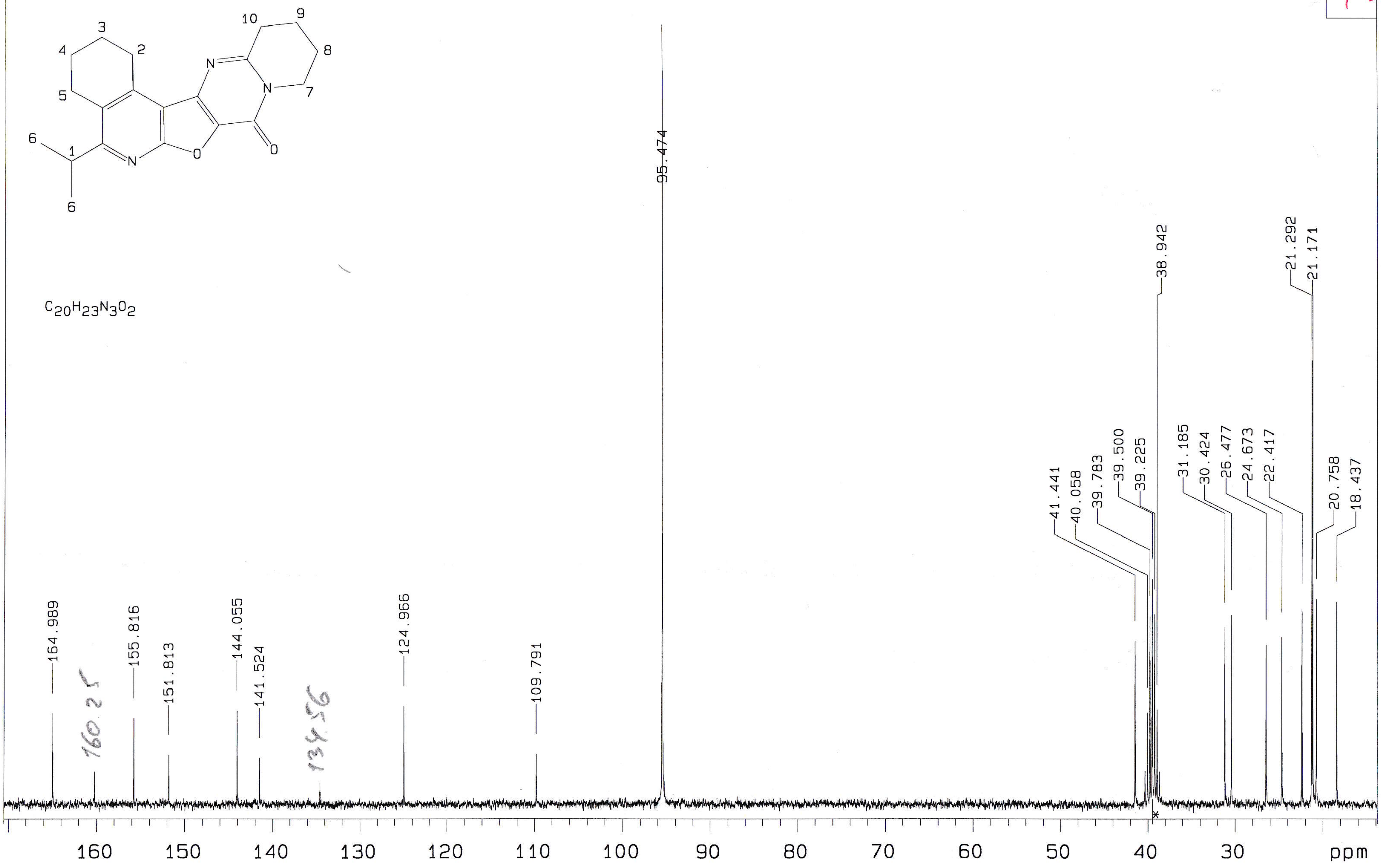

Molecular Structure Research Centre, Yerevan, Armenia, Varian Mercury-300VX

H1 300.088 MHz, nt = 16, np = 32000, temp = 30.0 C, lb = -0.2, solvent = DMSO/CCl4 1/3

Mar 9 2017

HA-966

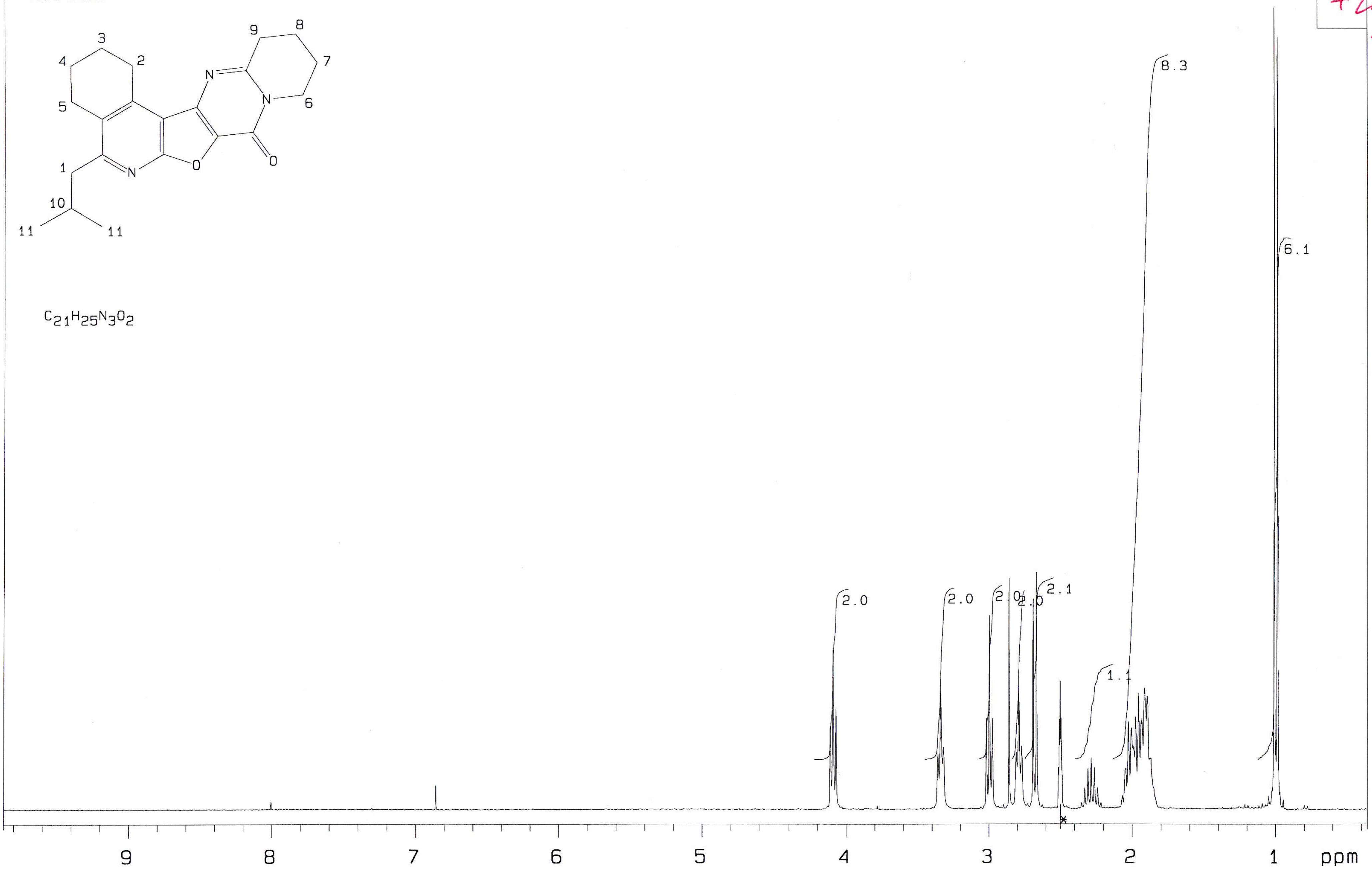

Molecular Structure Research Centre, Yerevan, Armenia, Varian Mercury-300VX

C13 75.465 MHz, nt = 640, np = 19998, temp = 30.0 C, lb = 1.0, solvent = DMSO/CCl4 1/3

Mar 9 2017

6g

HA-966

SAMV_17 ha-966

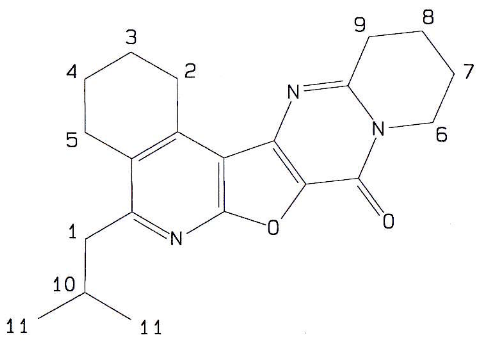

$C_{21}H_{25}N_3O_2$

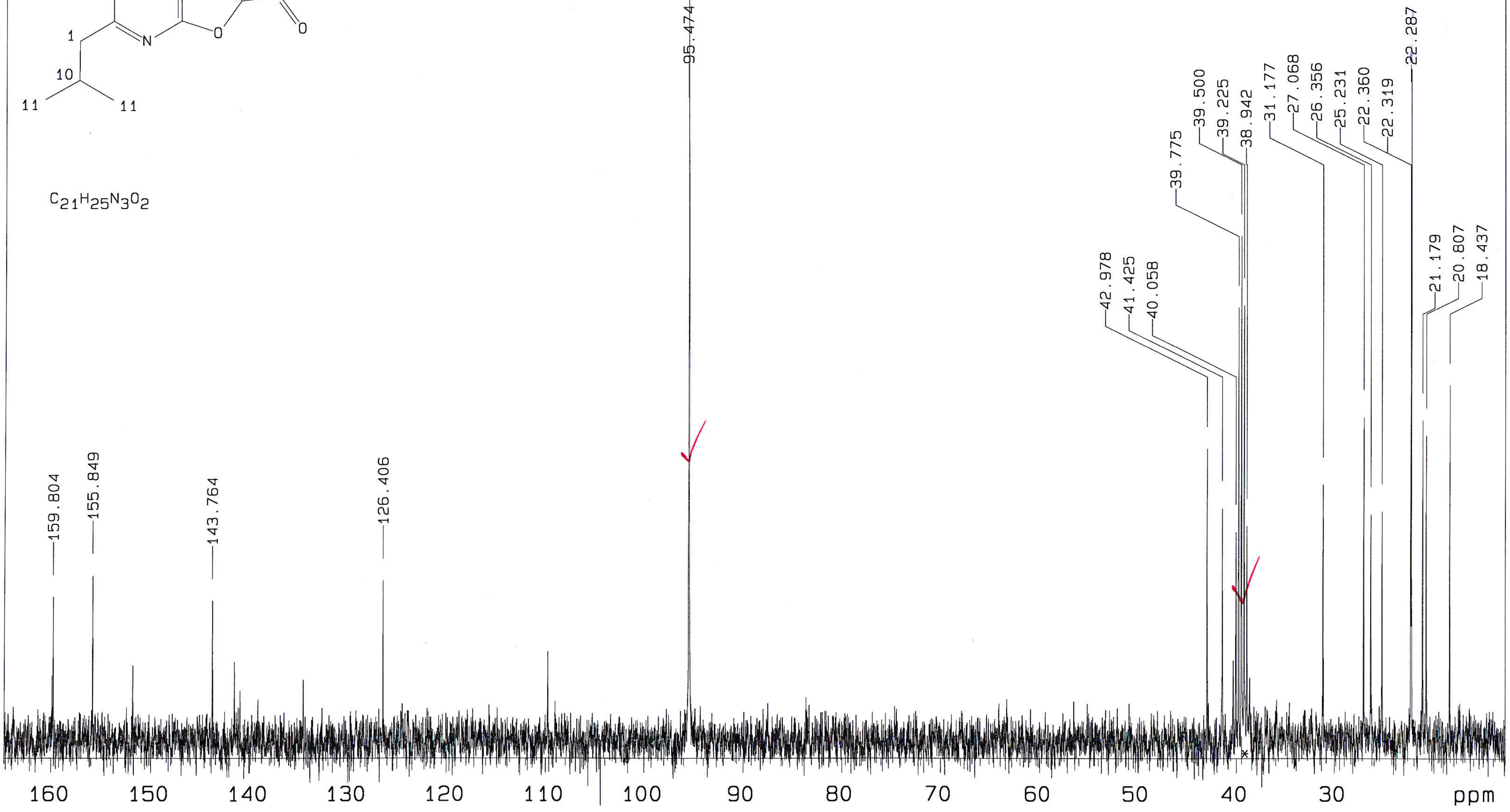

6h

Molecular Structure Research Centre, Yerevan, Armenia, Varian Mercury-300VX

H1 300.088 MHz, nt = 16, np = 32000, temp = 30.0 C, lb = -0.2, solvent = DMSO/CCl4 1/3

Feb 13 2017

SAMV_17 ha-951

HA-951

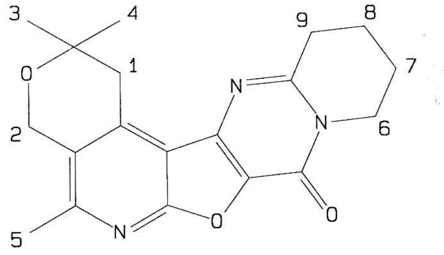

$C_{19}H_{21}N_3O_3$

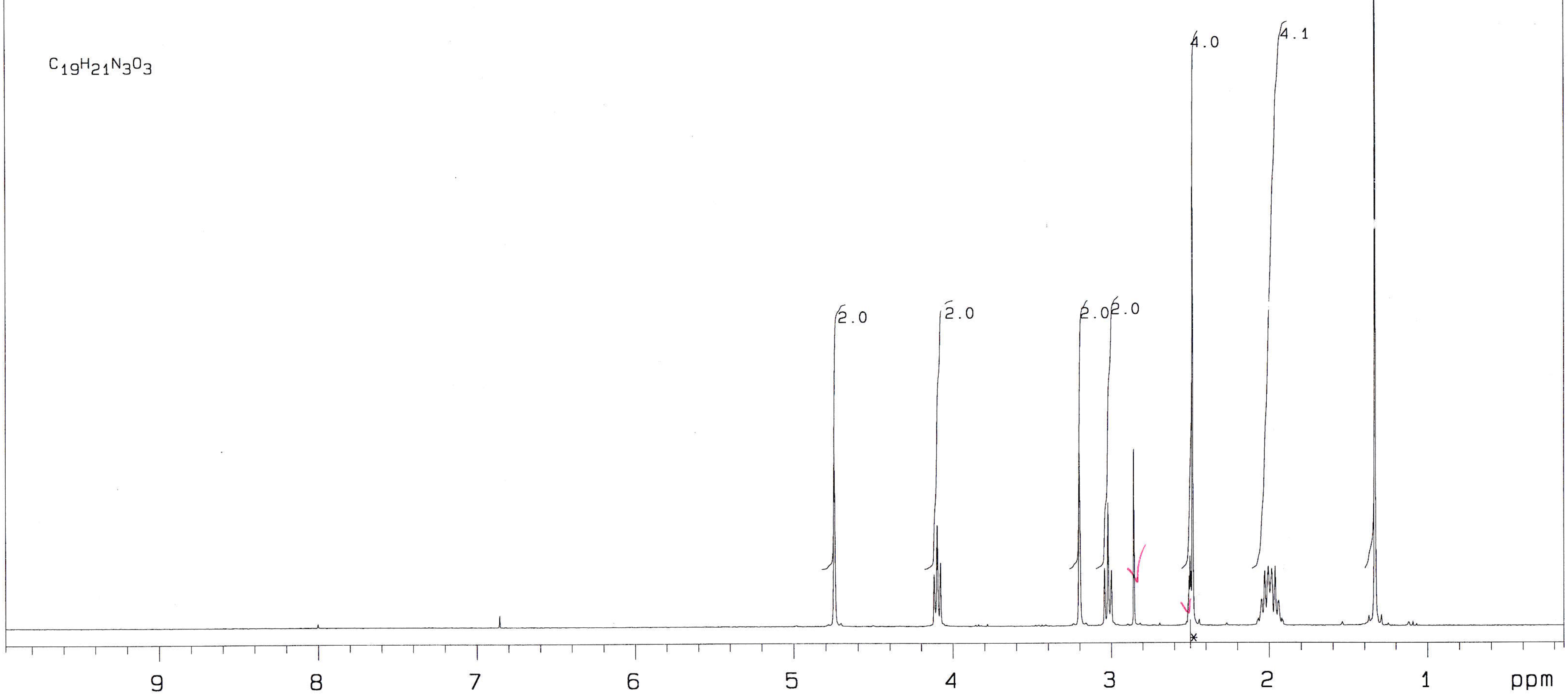

6h

Molecular Structure Research Centre, Yerevan, Armenia, Varian Mercury-300VX

C13 75.465 MHz, nt = 256, np = 19998, temp = 30.0 C, lb = 1.0, solvent = DMSO/CCl4 1/3

Feb 13 2017

SAMV_17 ha-951

HA-951

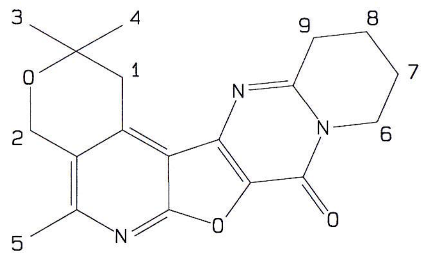

$C_{19}H_{21}N_3O_3$

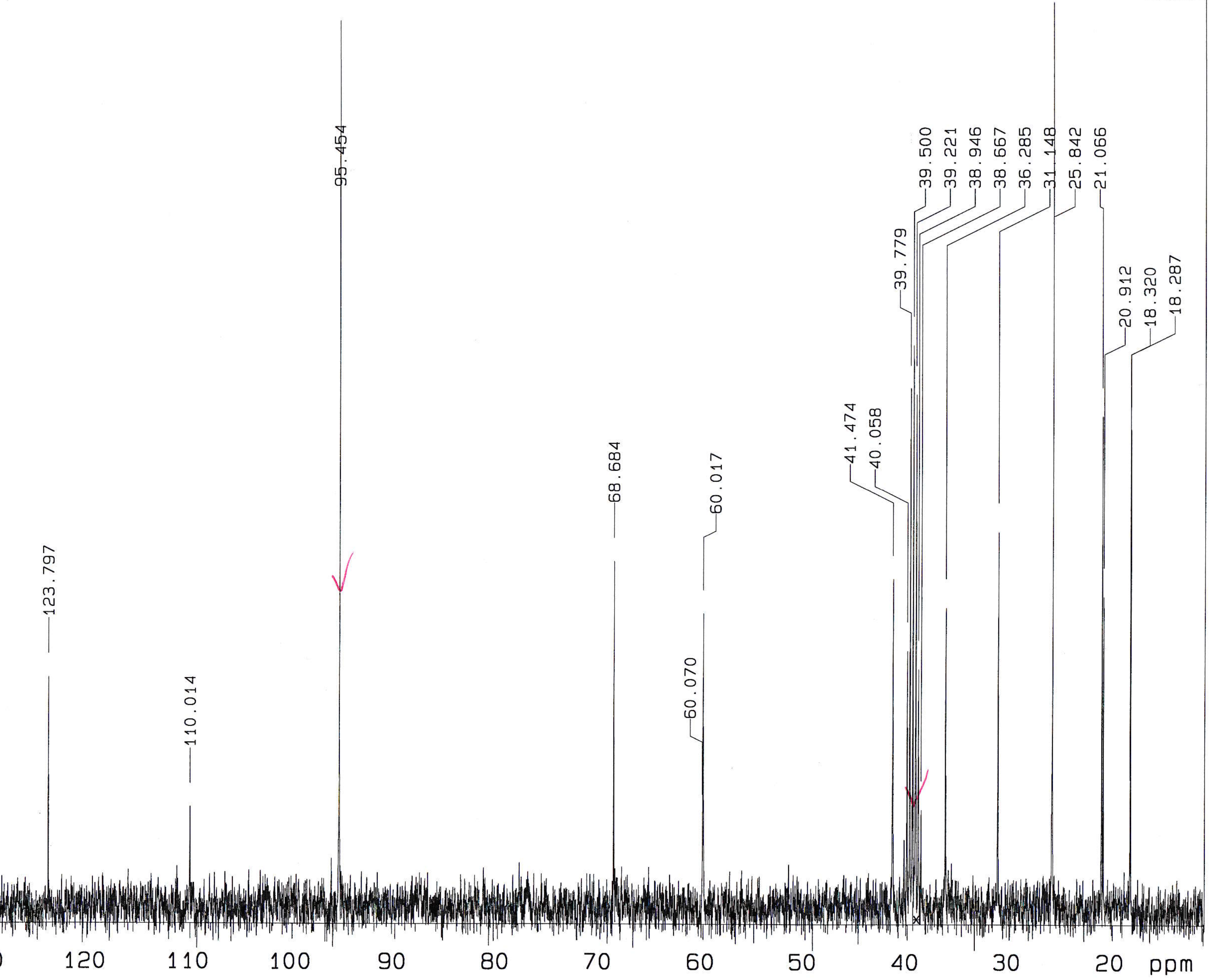

6i

Molecular Structure Research Centre, Yerevan, Armenia, Varian Mercury-300VX

H1 300.088 MHz, nt=16, np=32000, temp=30.0 C, lb=-0.2, solvent=DMSO/CCl4 1/3

Feb 10 2017

SAMV_17 ha-948

HA-948

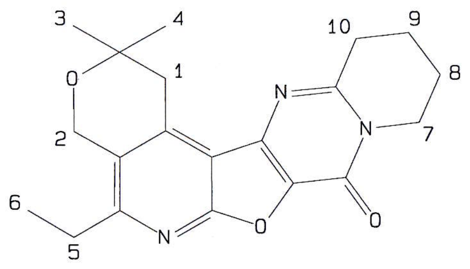

$C_{20}H_{23}N_3O_3$

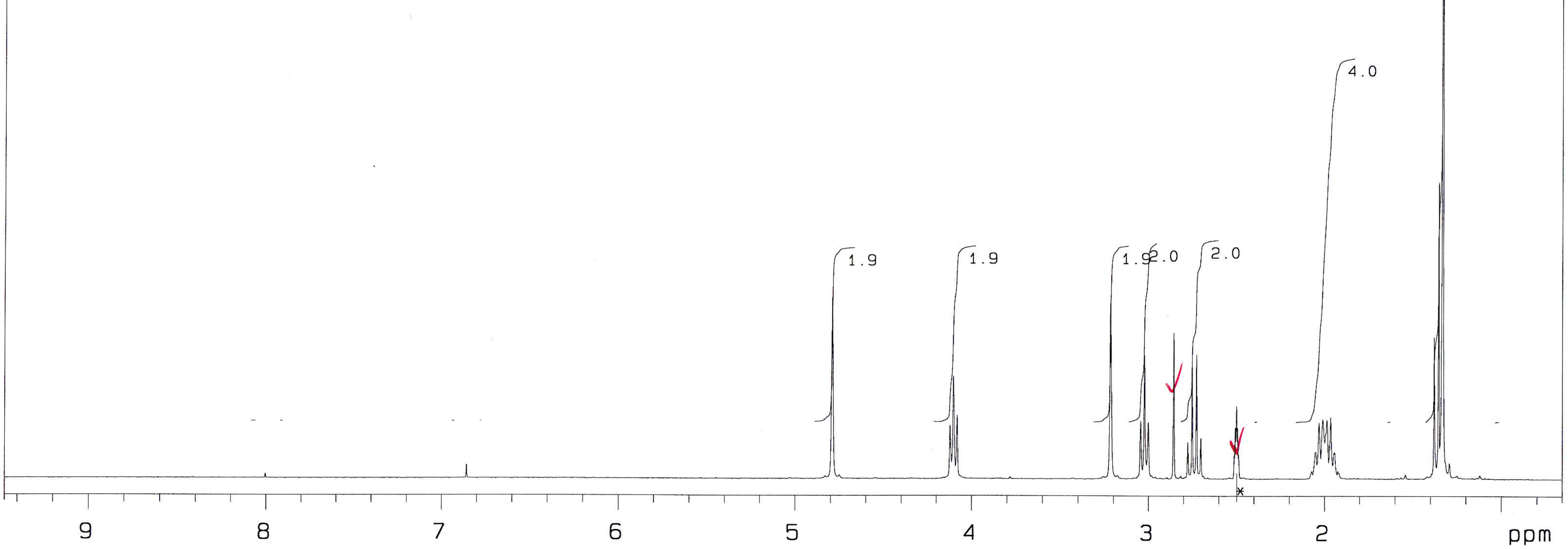

6i

Molecular Structure Research Centre, Yerevan, Armenia, Varian Mercury-300VX

C13 75.465 MHz, nt = 656, np = 19998, temp = 30.0 C, lb = 1.0, solvent = DMSO/CCl4 1/3

Feb 10 2017

SAMV_17 ha-948

HA-948

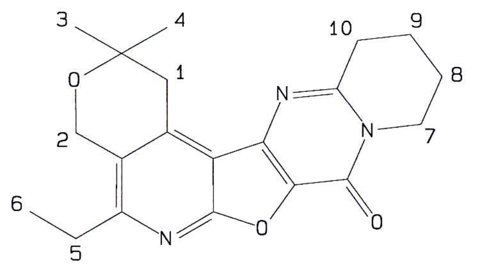

$C_{20}H_{23}N_3O_3$

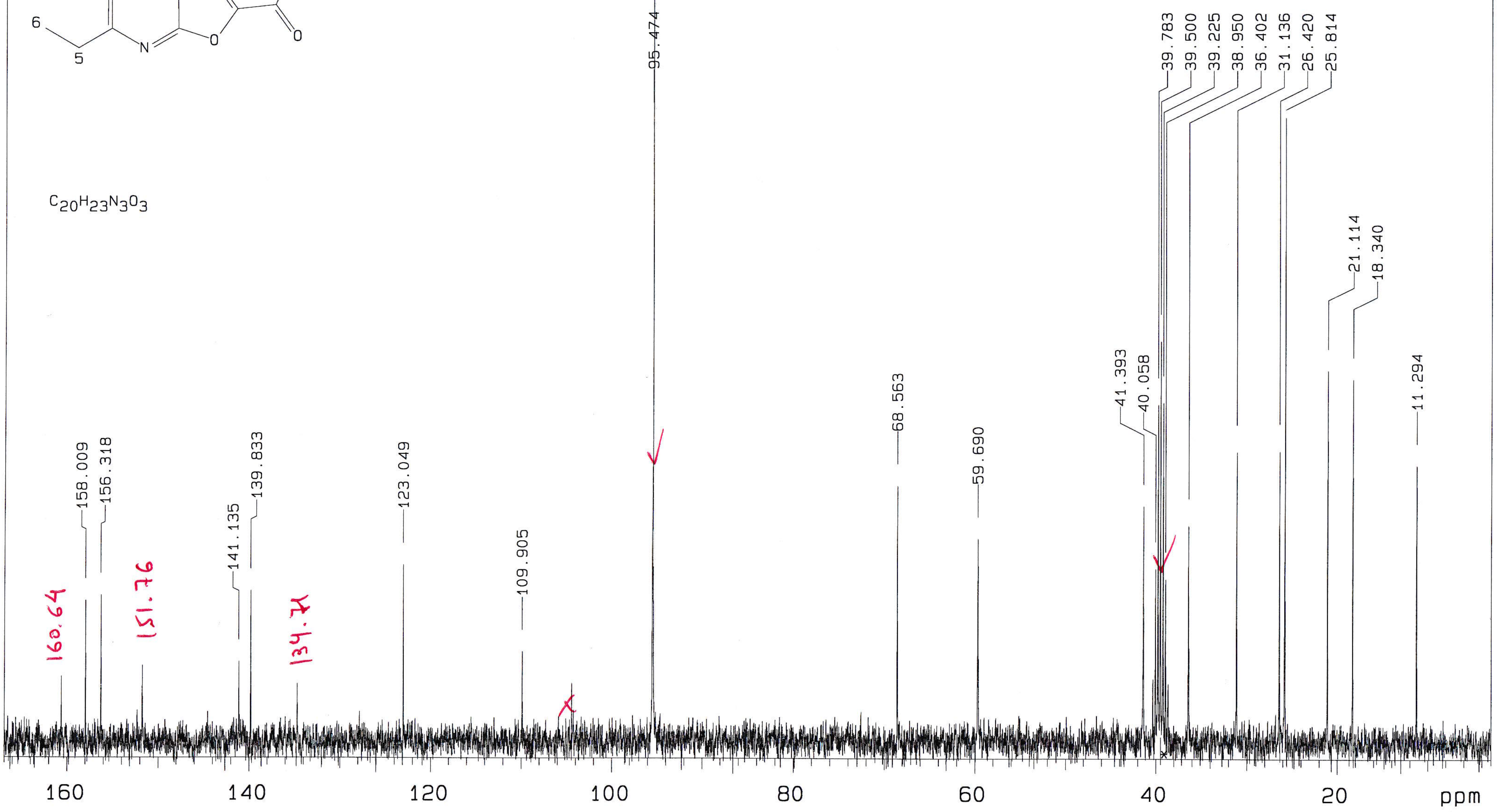

6j

Molecular Structure Research Centre, Yerevan, Armenia, Varian Mercury-300VX **H1 300.088 MHz, nt=16, np=32000, temp=30.0 C, lb=-0.2, solvent=DMSO/CCl4 1/3** **Dec 23 2016**

SAMV_16 ha-943

HA-943

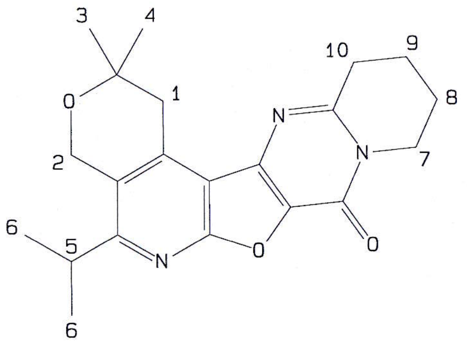

$C_{21}H_{25}N_3O_3$

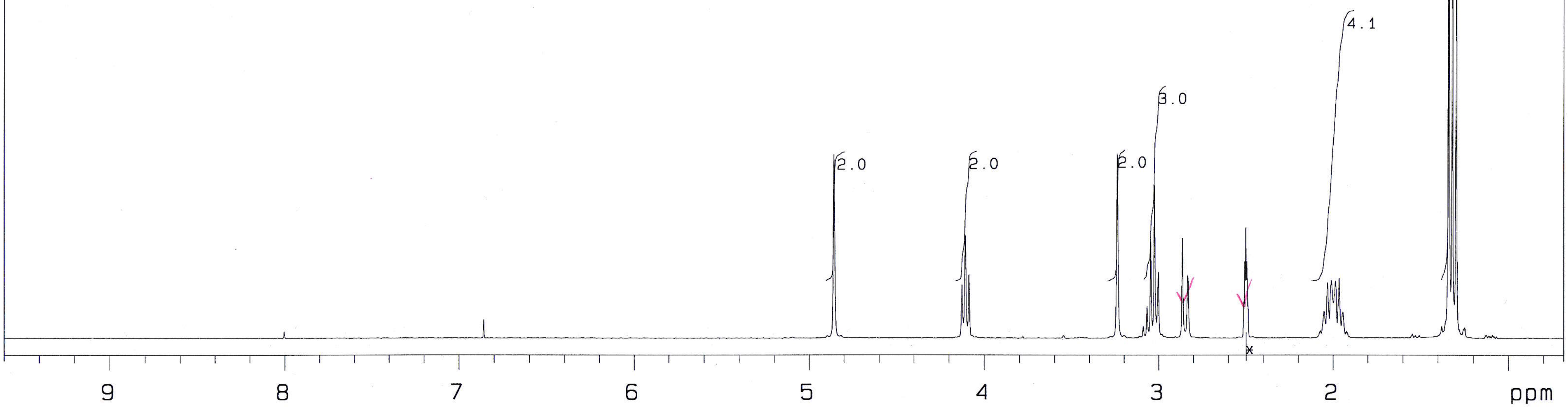

6j

Molecular Structure Research Centre, Yerevan, Armenia, Varian Mercury-300VX

C13 75.465 MHz, nt=848, np=19998, temp=30.0 C, lb=1.0, solvent=DMSO/CCl4 1/3

Dec 23 2016

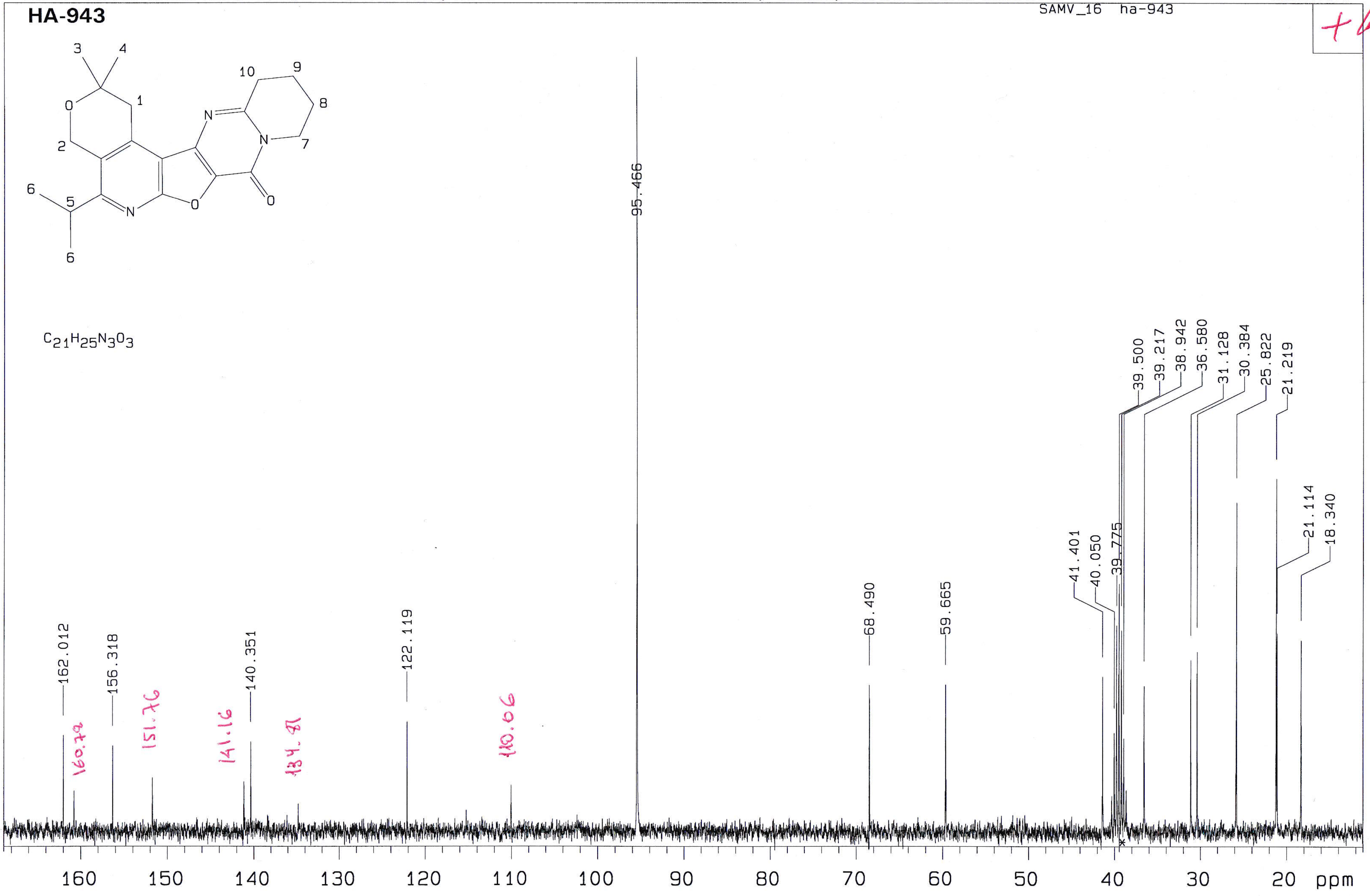

6K

Molecular Structure Research Centre, Yerevan, Armenia, Varian Mercury-300VX

H1 300.088 MHz, nt = 16, np = 32000, temp = 30.0 C, lb = -0.2, solvent = DMSO/CCl4 1/3

Feb 1 2017

HA-944

NOCI_17 ha-944

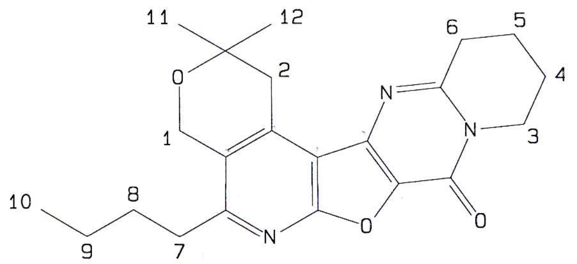

$C_{22}H_{27}N_3O_3$

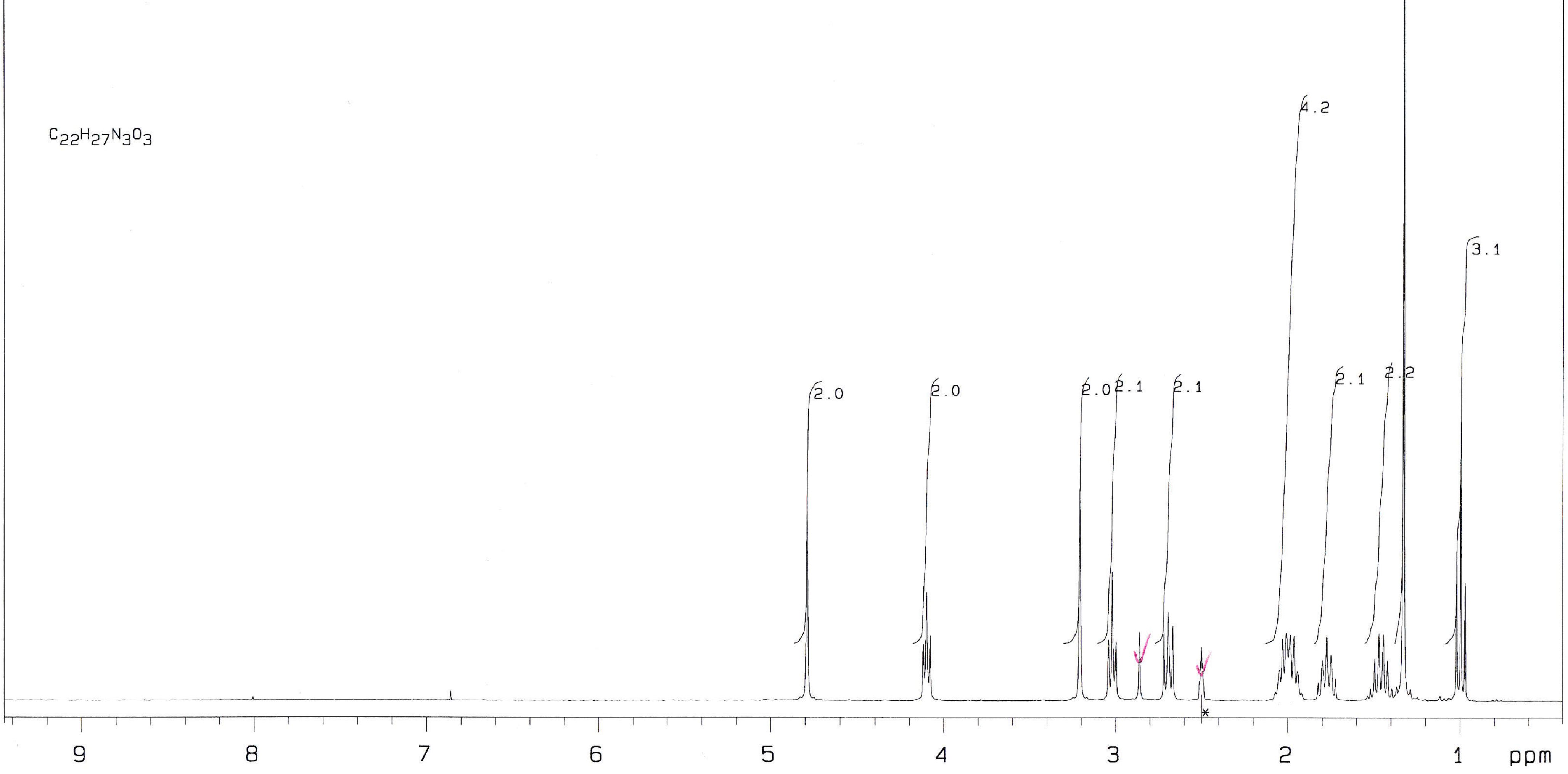

6K

Molecular Structure Research Centre, Yerevan, Armenia, Varian Mercury-300VX **C13 75.465 MHz, nt=1360, np=19998, temp=30.0 C, lb=1.0, solvent=DMSO/CCl4 1/3** **Feb 1 2017**

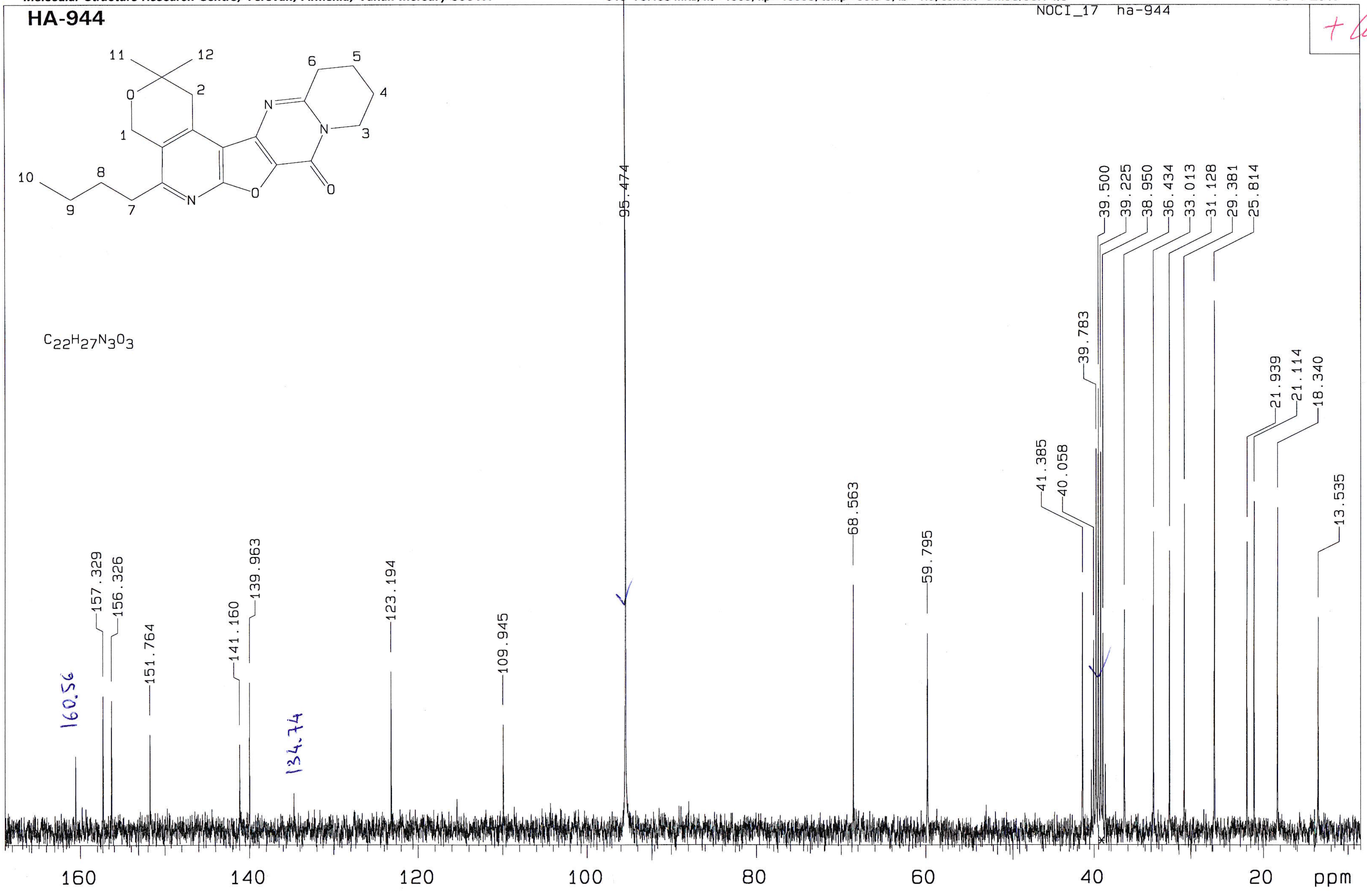

6l

Molecular Structure Research Centre, Yerevan, Armenia, Varian Mercury-300VX

H1 300.088 MHz, nt = 16, np = 32000, temp = 30.0 C, lb = -0.2, solvent = DMSO/CCl4 1/3

May 23 2019

SAMV_19 ha-1044

HA-1044

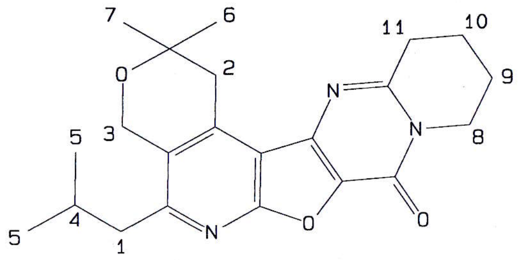

$C_{22}H_{27}N_3O_3$

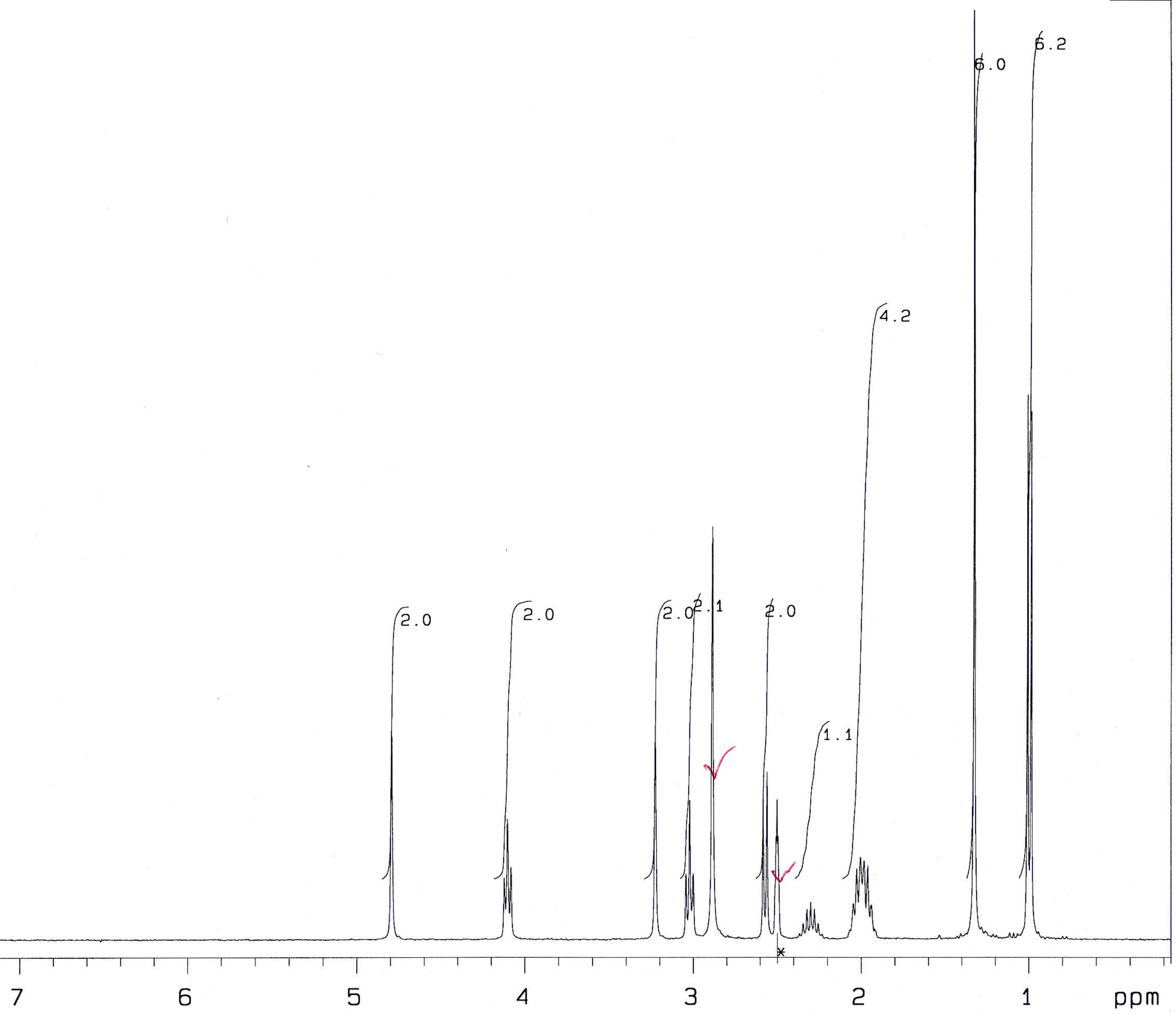

Molecular Structure Research Centre, Yerevan, Armenia, Varian Mercury-300VX

C13 75.465 MHz, nt=256, np=19998, temp=30.0 C, lb=1.0, solvent=DMSO/CCl4 1/3

May 23 2019

SAMV_19 ha-1044

HA-1044

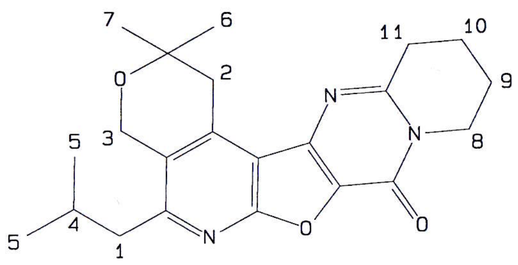

$C_{22}H_{27}N_3O_3$

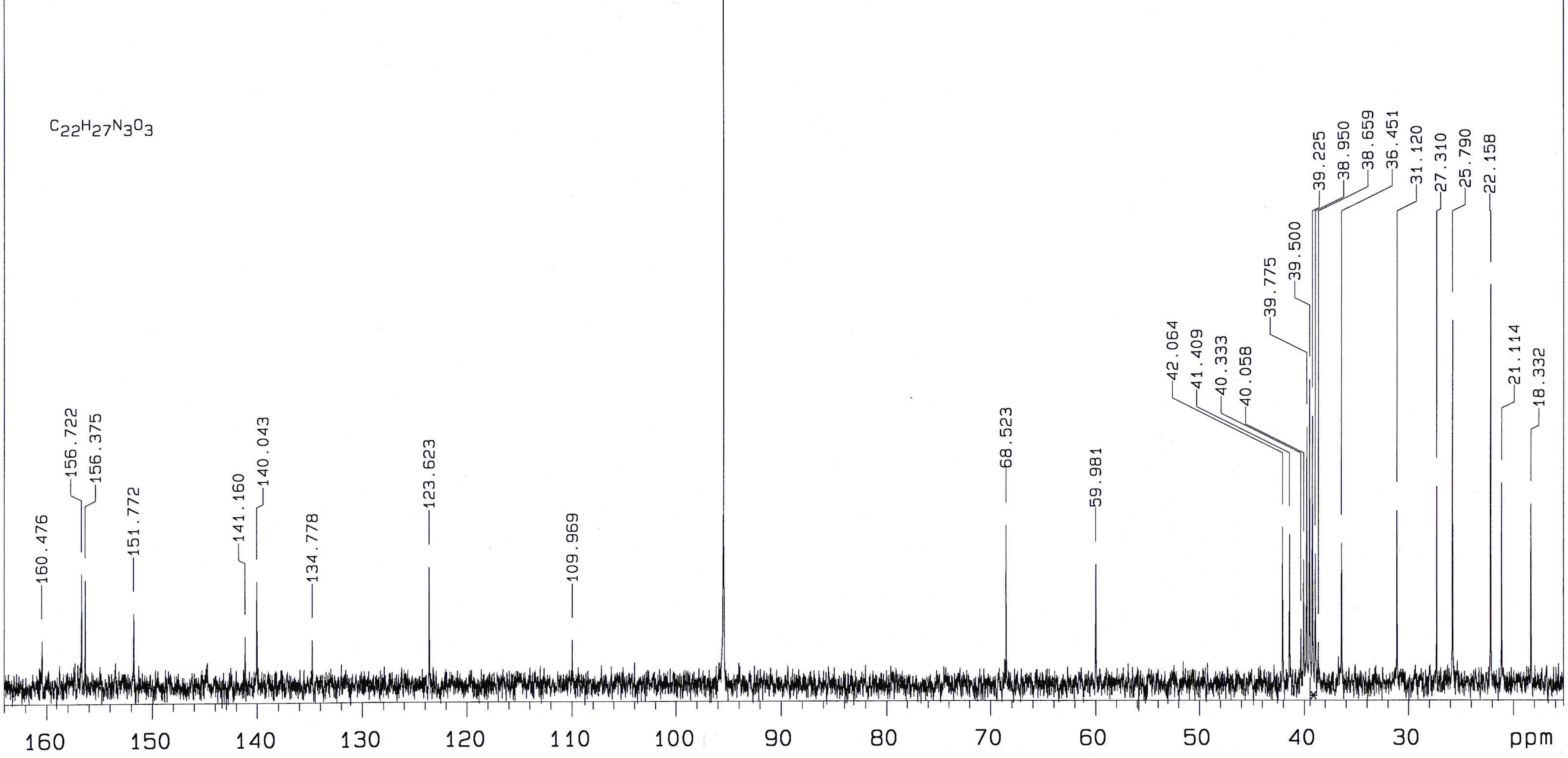

6m

Molecular Structure Research Centre, Yerevan, Armenia, Varian Mercury-300VX

H1 300.088 MHz, nt = 16, np = 32000, temp = 30.0 C, lb = -0.2, solvent = DMSO/CCl4 1/3

Feb 21 2017

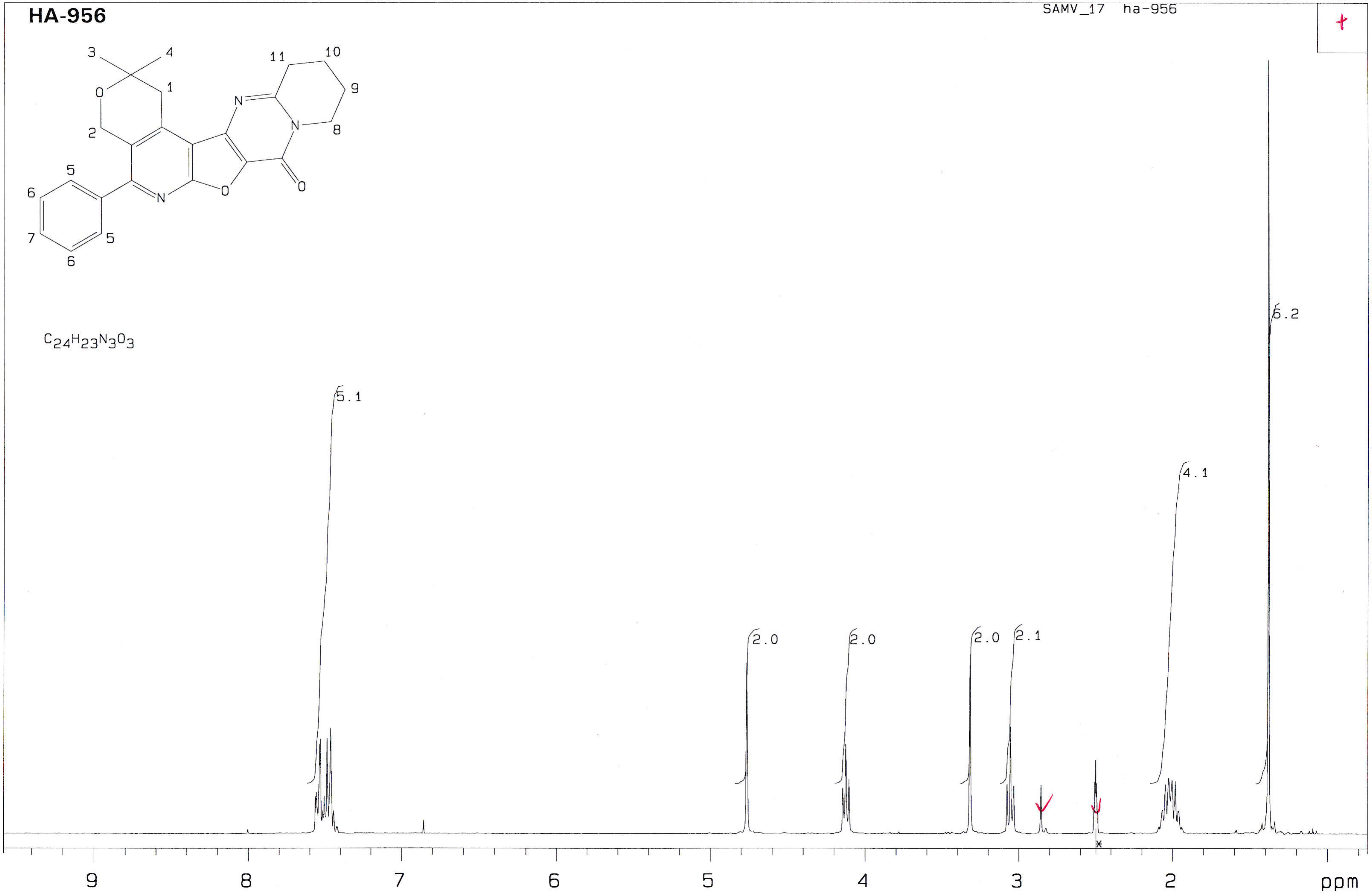

6 m

Molecular Structure Research Centre, Yerevan, Armenia, Varian Mercury-300VX

C13 75.465 MHz, nt = 1280, np = 19998, temp = 30.0 C, lb = 1.0, solvent = DMSO/CCl4 1/3

Feb 21 2017

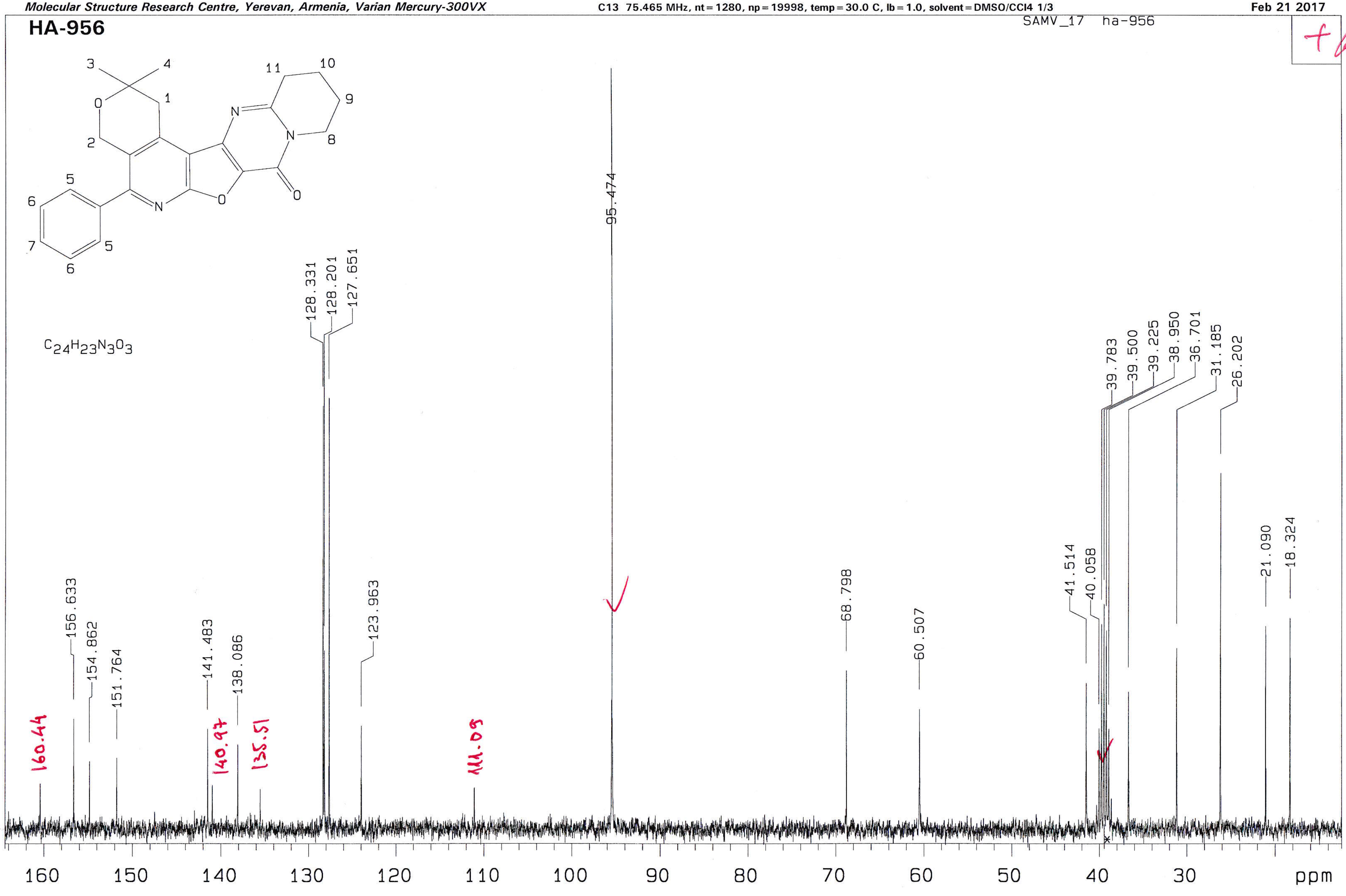

6n

Molecular Structure Research Centre, Yerevan, Armenia, Varian Mercury-300VX

H1 300.088 MHz, nt = 16, np = 32000, temp = 30.0 C, lb = -0.2, solvent = DMSO/CCl4 1/3

Feb 8 2017

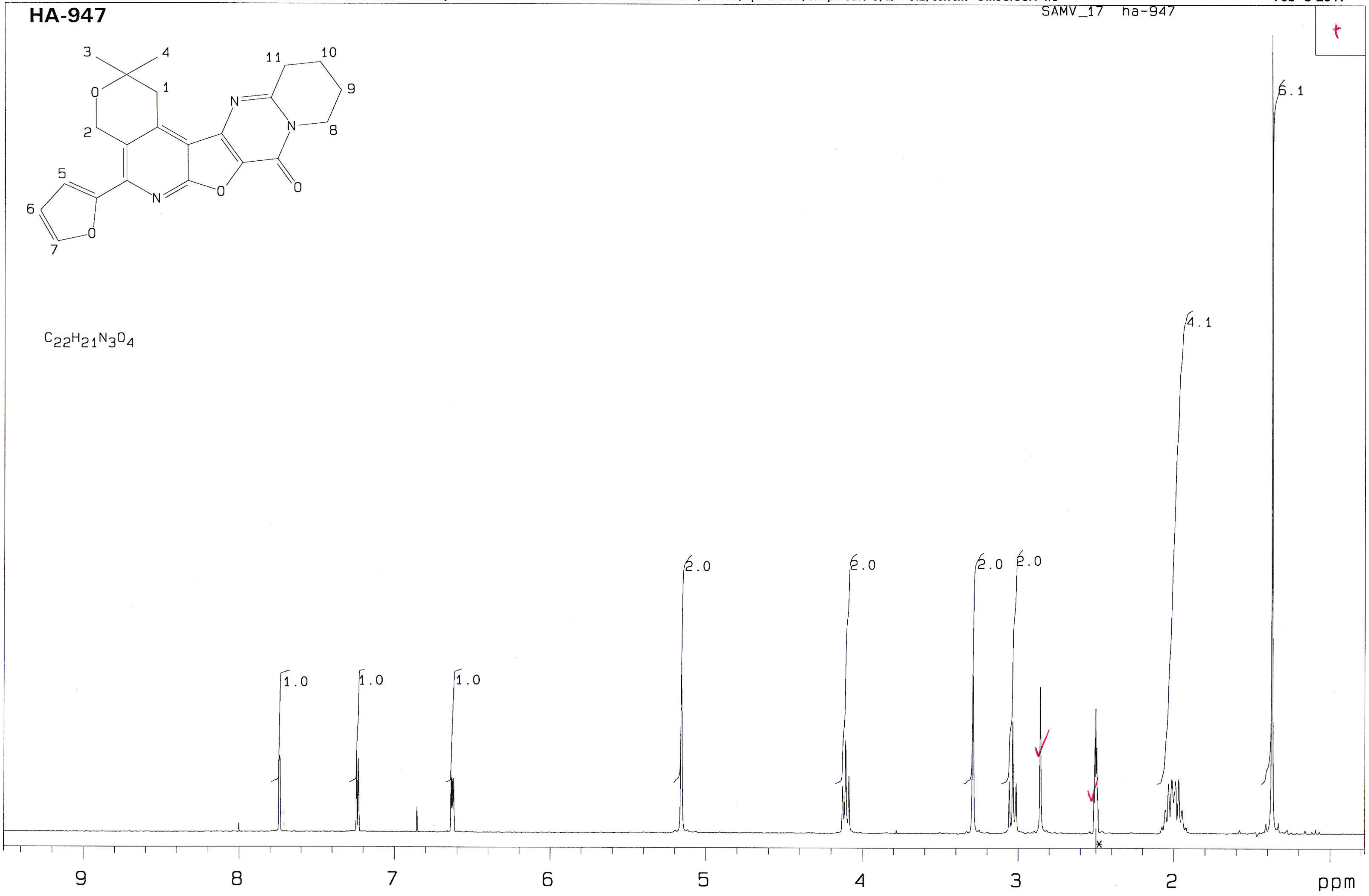

Molecular Structure Research Centre, Yerevan, Armenia, Varian Mercury-300VX

C13 75.465 MHz, nt=960, np=19998, temp=30.0 C, lb=1.0, solvent=DMSO/CCl4 1/3

Feb 8 2017

SAMV_17 ha-947

HA-947

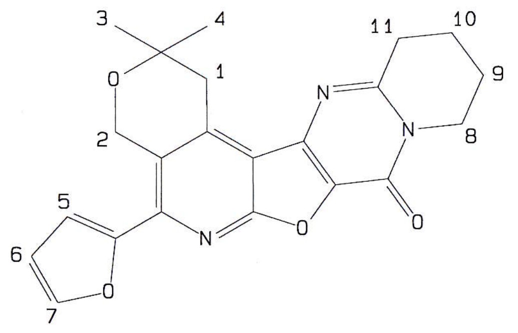

$C_{22}H_{21}N_3O_4$

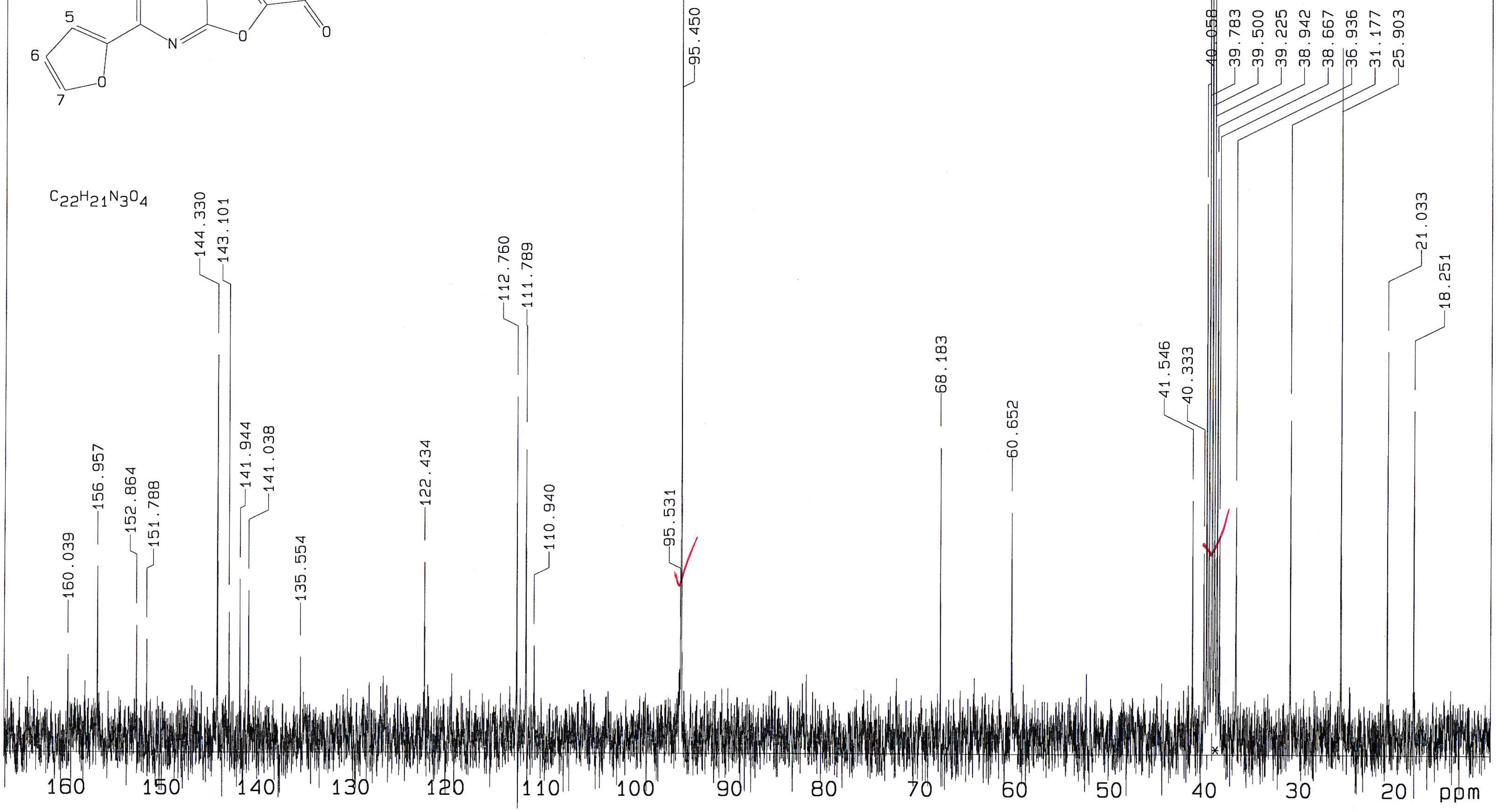

7a

Molecular Structure Research Centre, Yerevan, Armenia, Varian Mercury-300VX

H1 300.088 MHz, nt = 16, np = 32000, temp = 30.0 C, lb = -0.2, solvent = DMSO/CCl4 1/3

Feb 22 2017

HA-957

SAMV_17 ha-957

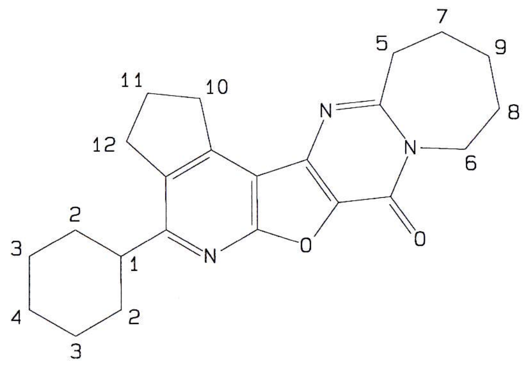

$C_{23}H_{27}N_3O_2$

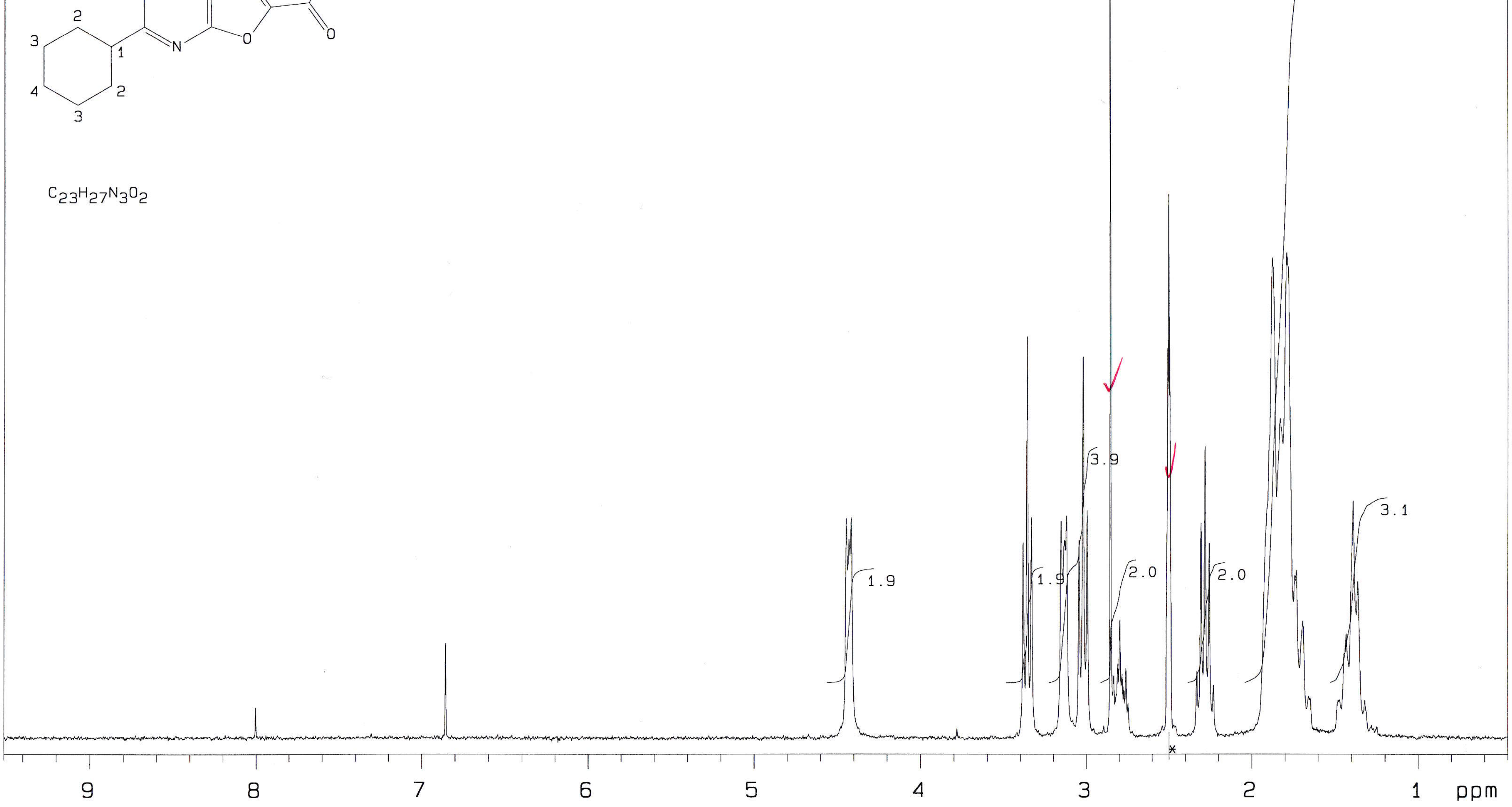

7a

Molecular Structure Research Centre, Yerevan, Armenia, Varian Mercury-300VX

C13 75.465 MHz, nt = 512, np = 19998, temp = 30.0 C, lb = 1.0, solvent = DMSO/CCl4 1/3

Feb 22 2017

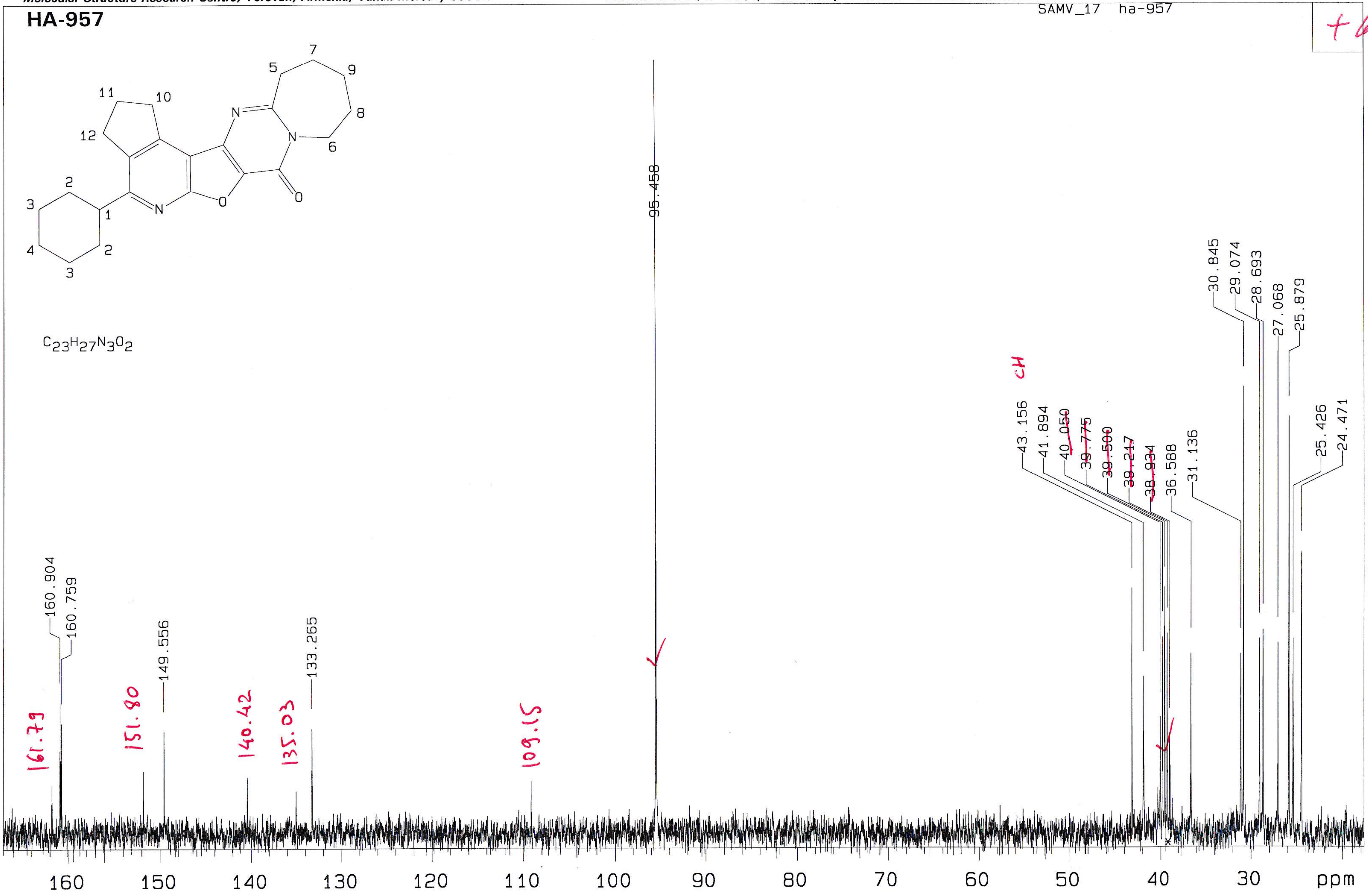

7b

Molecular Structure Research Centre, Yerevan, Armenia, Varian Mercury-300VX

H1 300.088 MHz, nt=16, np=32000, temp=30.0 C, lb=-0.2, solvent=DMSO/CCl4 1/3

Mar 1 2017

HA-961

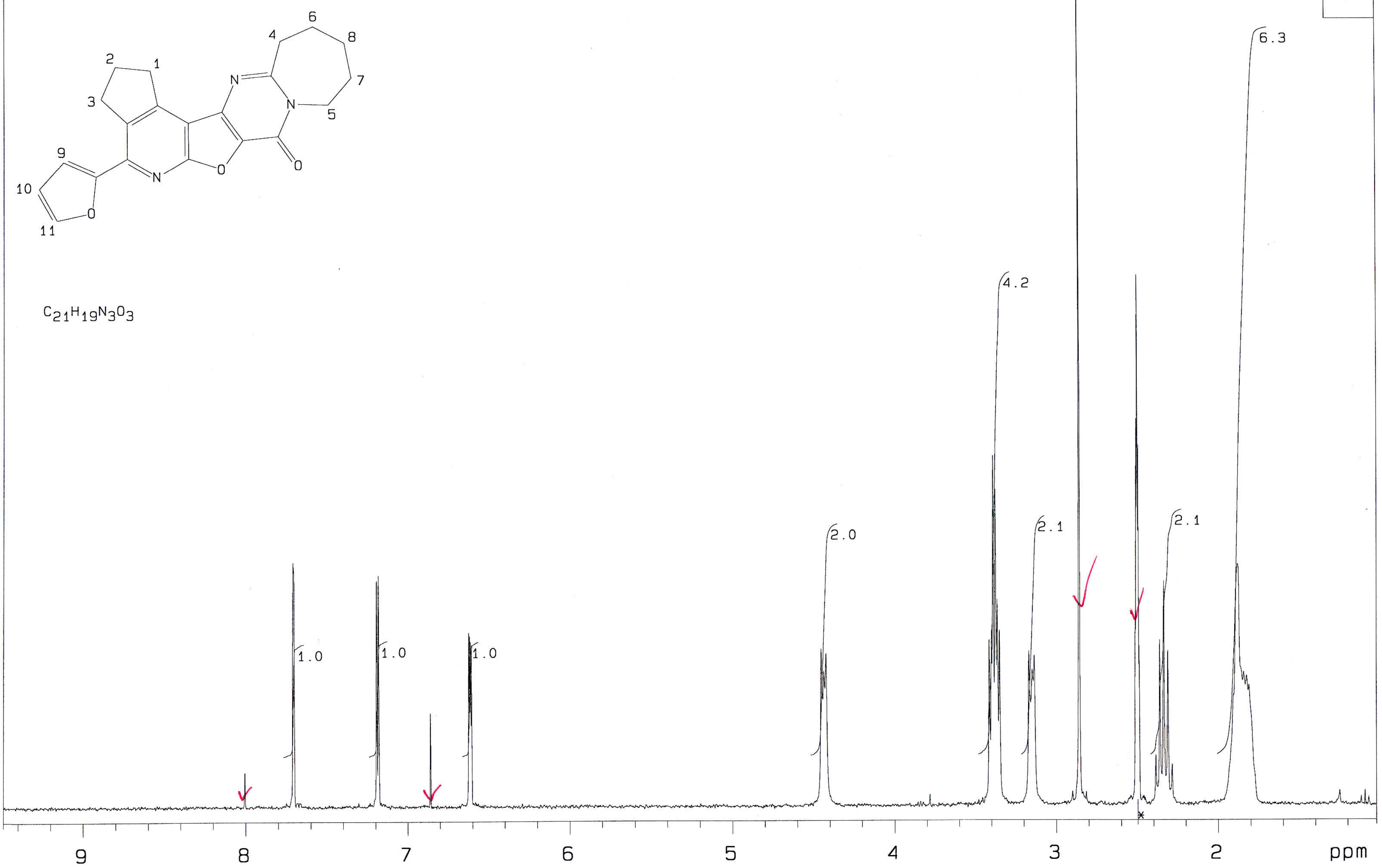

76

Molecular Structure Research Centre, Yerevan, Armenia, Varian Mercury-300VX

C13 75.465 MHz, nt=4272, np=19998, temp=30.0 C, lb=1.0, solvent=DMSO/CCl4 1/3

Mar 1 2017

NOCI_17 ha-961

HA-961

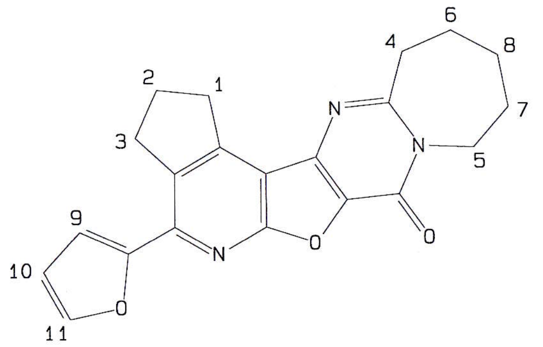

$C_{21}H_{19}N_3O_3$

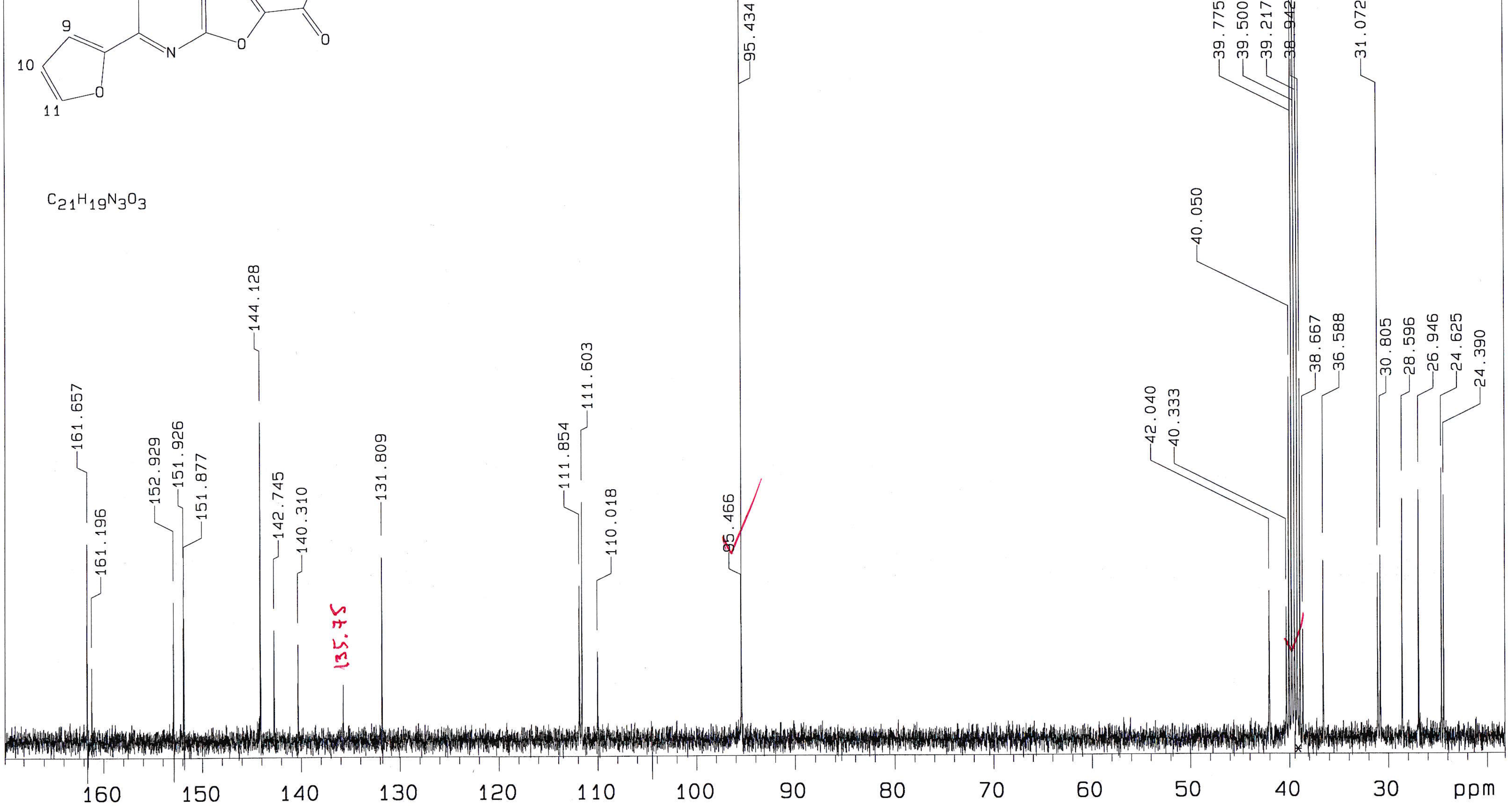

7c

Molecular Structure Research Centre, Yerevan, Armenia, Varian Mercury-300VX

H1 300.088 MHz, nt = 16, np = 16000, temp = 30.0 C, lb = -0.2, solvent = DMSO/CCl4 1/3

Apr 5 2017

NOCI_17 ha-971

HA-971

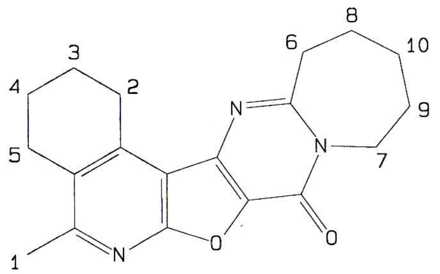

$C_{19}H_{21}N_3O_2$

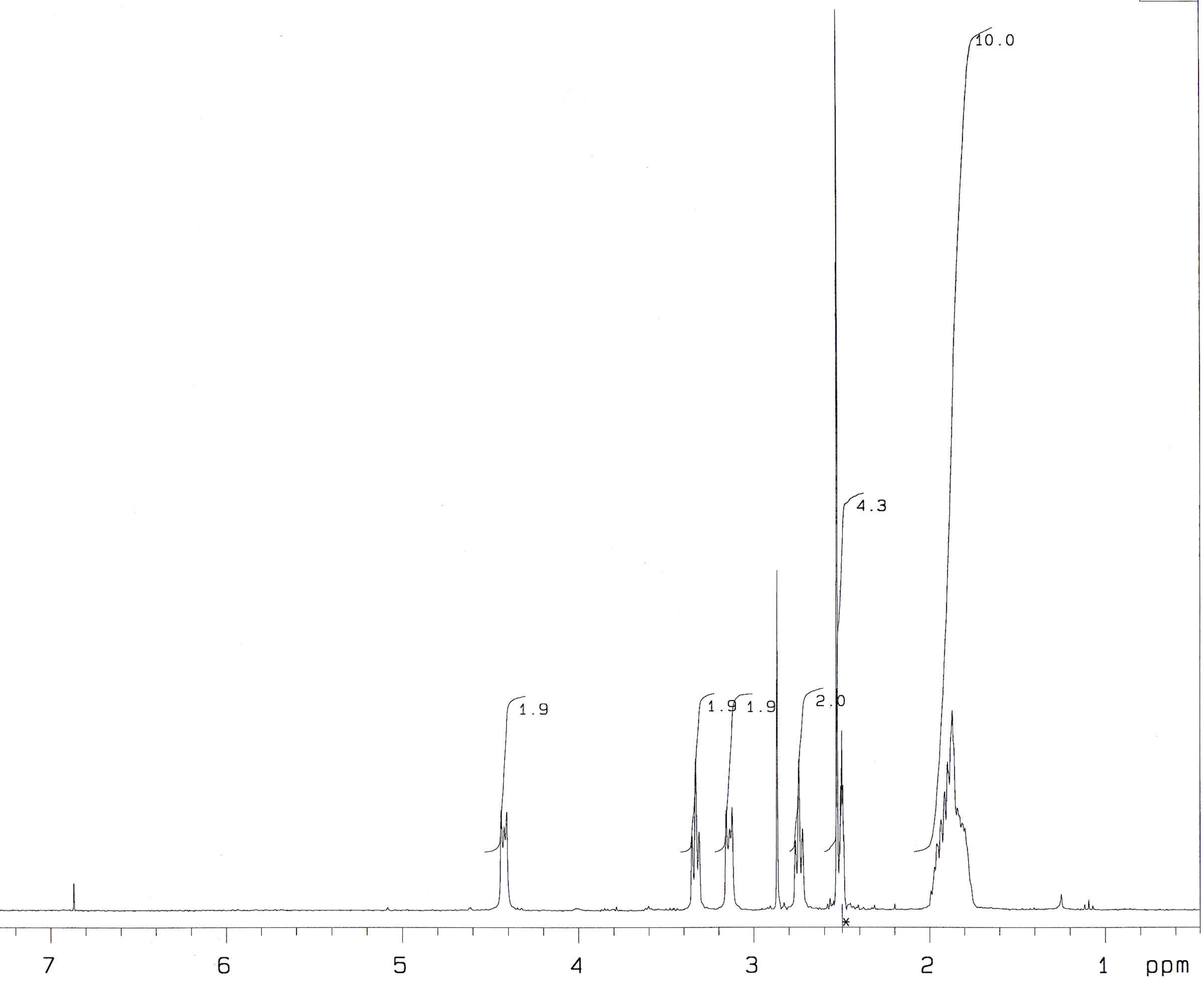

7c

Molecular Structure Research Centre, Yerevan, Armenia, Varian Mercury-300VX

C13 75.465 MHz, nt = 1504, np = 19998, temp = 30.0 C, lb = 1.0, solvent = DMSO/CCl4 1/3

Apr 5 2017

NOCI_17 ha-971

HA-971

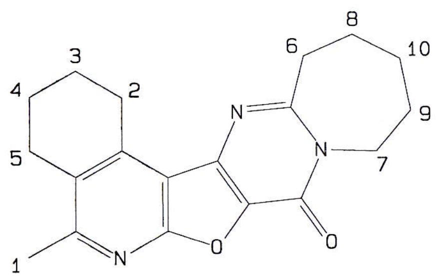

$C_{19}H_{21}N_3O_2$

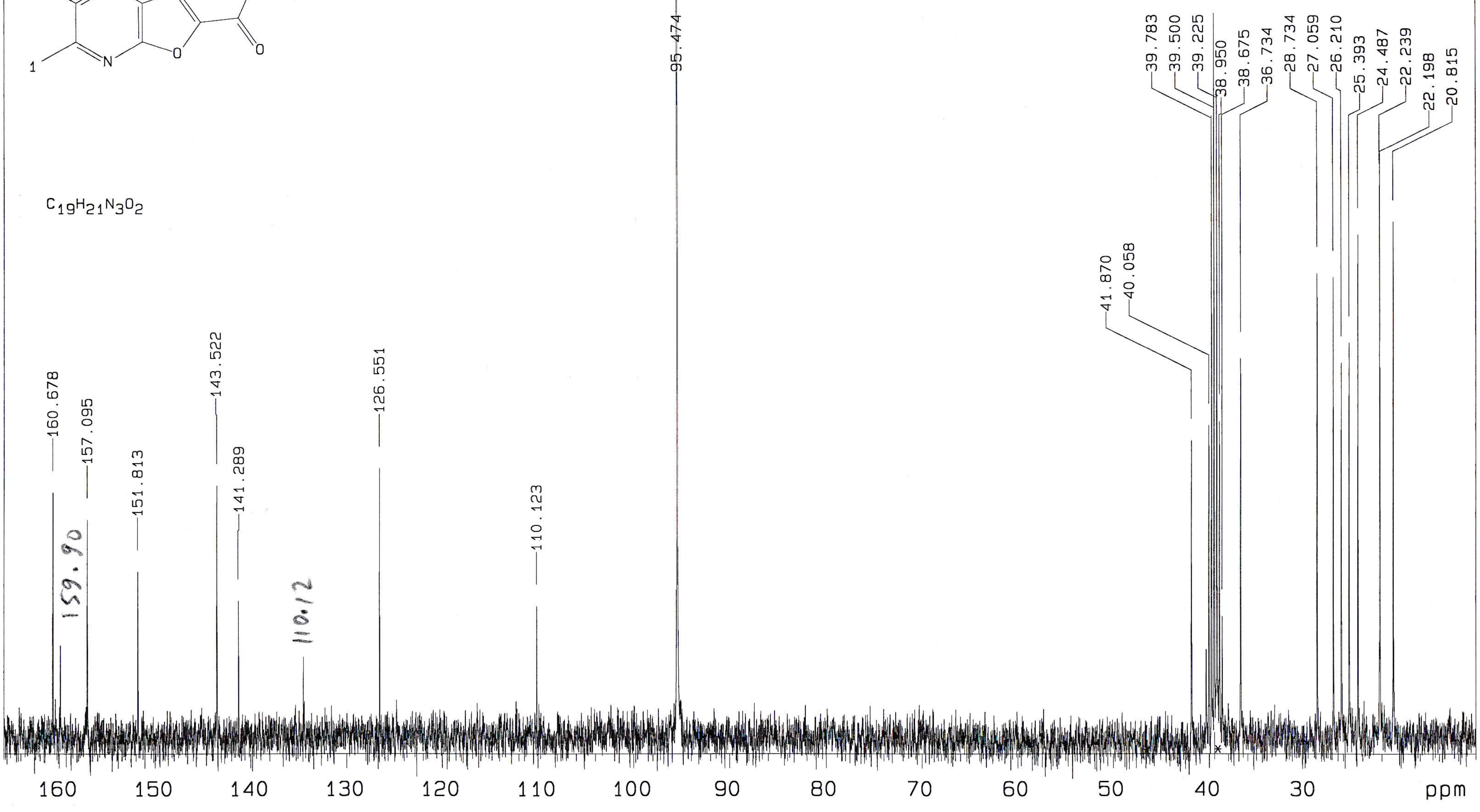

7d

Molecular Structure Research Centre, Yerevan, Armenia, Varian Mercury-300VX

H1 300.088 MHz, nt = 16, np = 32000, temp = 30.0 C, lb = -0.2, solvent = DMSO/CCl4 1/3

Mar 27 2017

SAMV_17 ha-968

HA-968

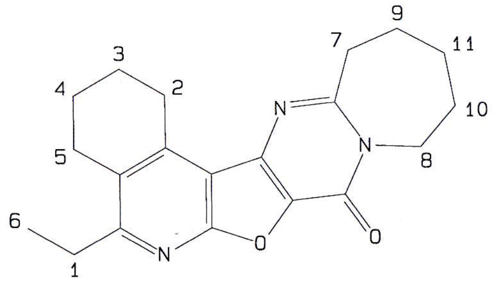

$C_{20}H_{23}N_3O_2$

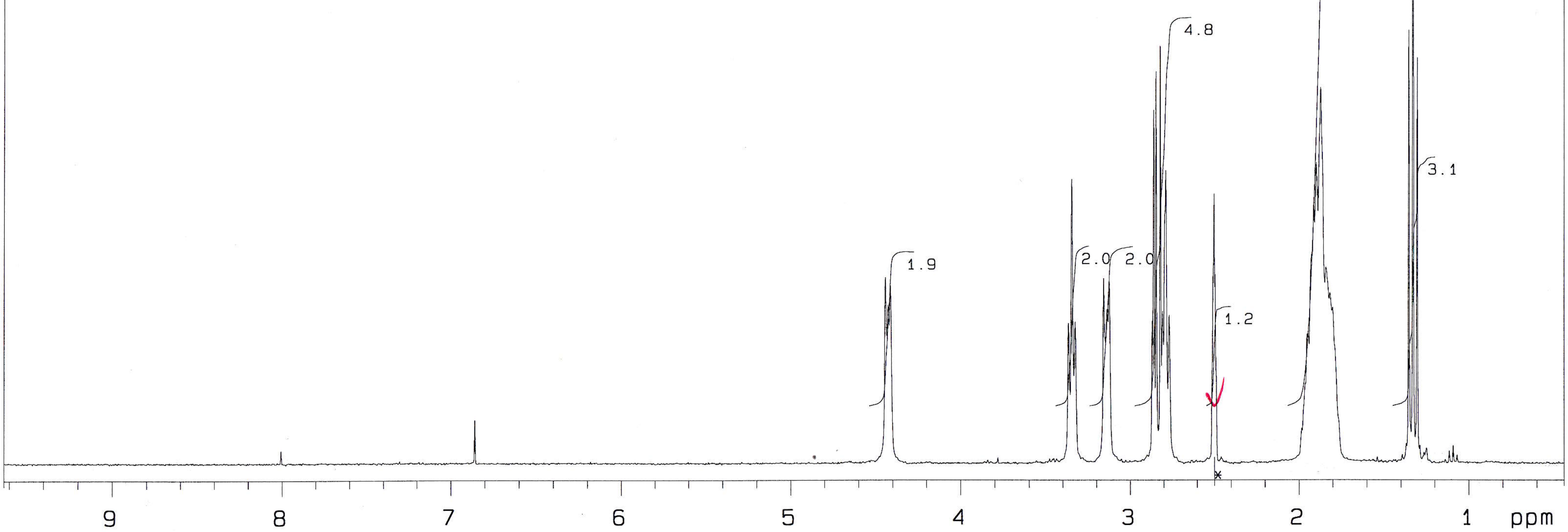

7d

Molecular Structure Research Centre, Yerevan, Armenia, Varian Mercury-300VX

C13 75.465 MHz, nt=624, np=19998, temp=30.0 C, lb=1.0, solvent=DMSO/CCl4 1/3

Mar 27 2017

SAMV_17 ha-968

HA-968

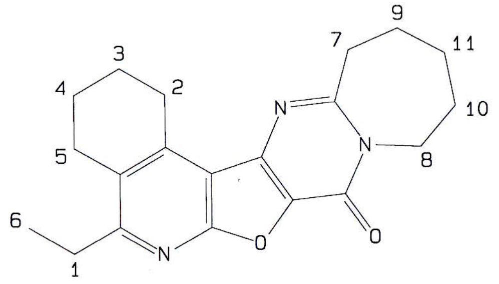

$C_{20}H_{23}N_3O_2$

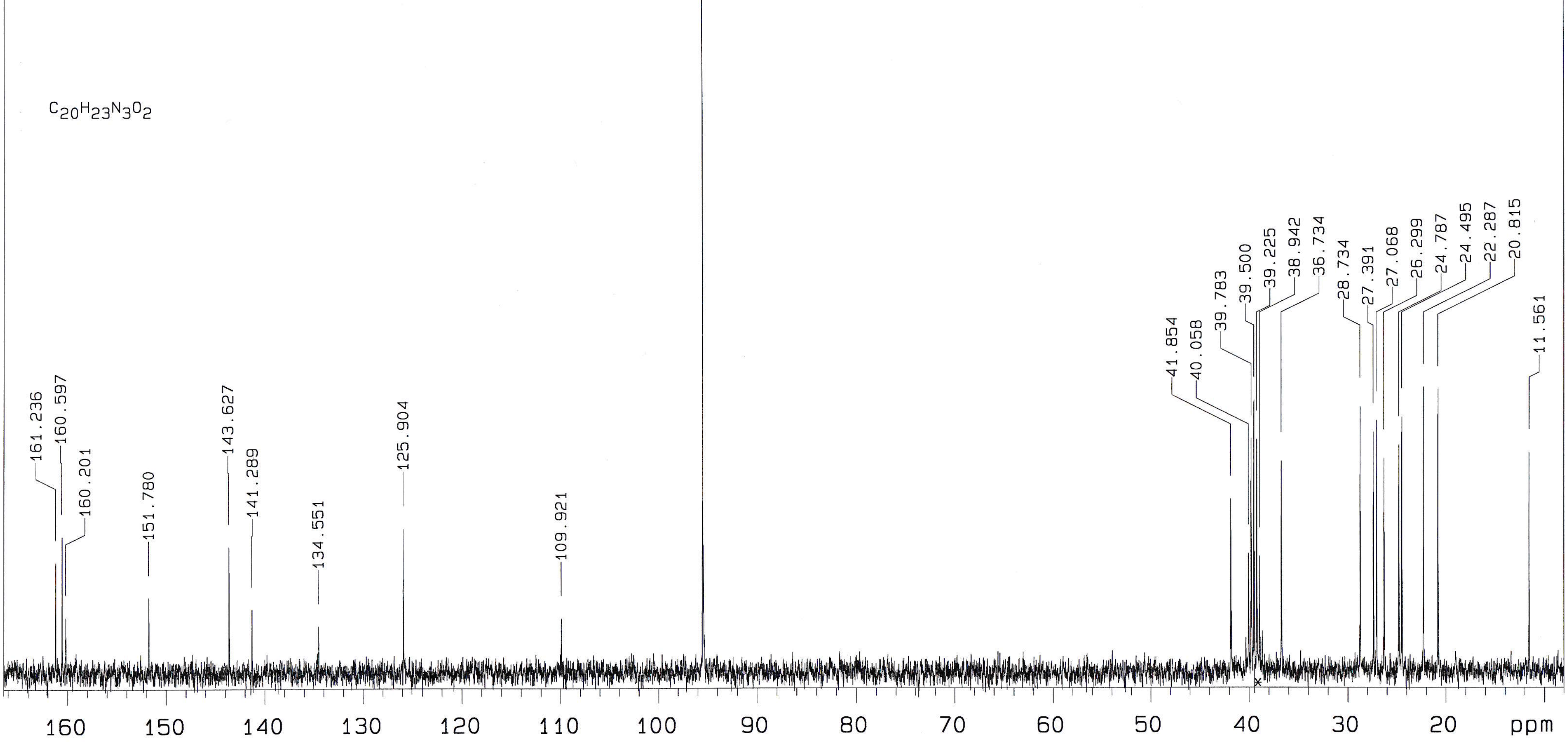

7e

Molecular Structure Research Centre, Yerevan, Armenia, Varian Mercury-300VX

H1 300.088 MHz, nt = 16, np = 32000, temp = 30.0 C, lb = -0.2, solvent = DMSO/CCl4 1/3

Feb 15 2017

HA-953

SAMV_17 ha-953

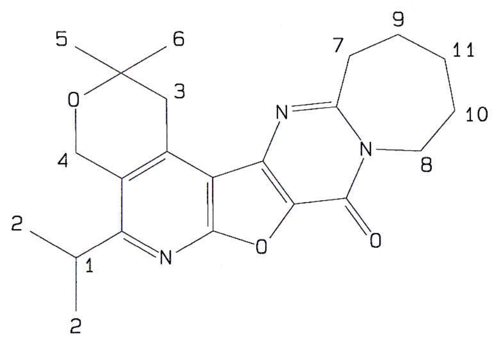

$C_{22}H_{27}N_3O_3$

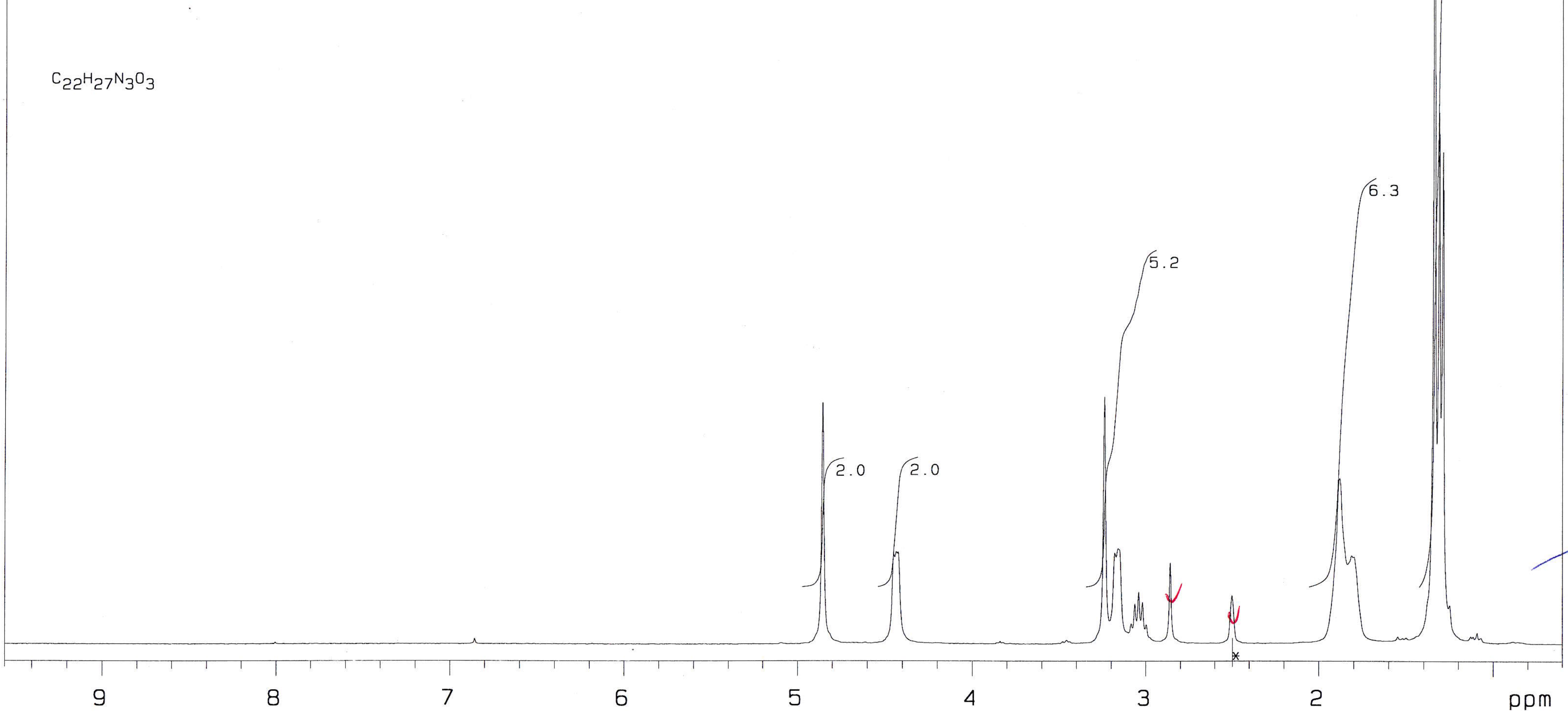

7e

Molecular Structure Research Centre, Yerevan, Armenia, Varian Mercury-300VX

C13 75.465 MHz, nt = 0, np = 19998, temp = 30.0 C, lb = 1.0, solvent = DMSO/CCl4 1/3

Feb 16 2017

HA-953

SAMV_17 ha-953

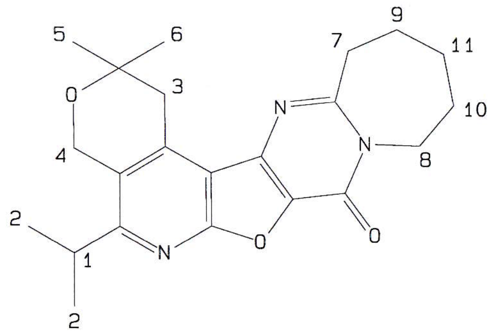

$C_{22}H_{27}N_3O_3$

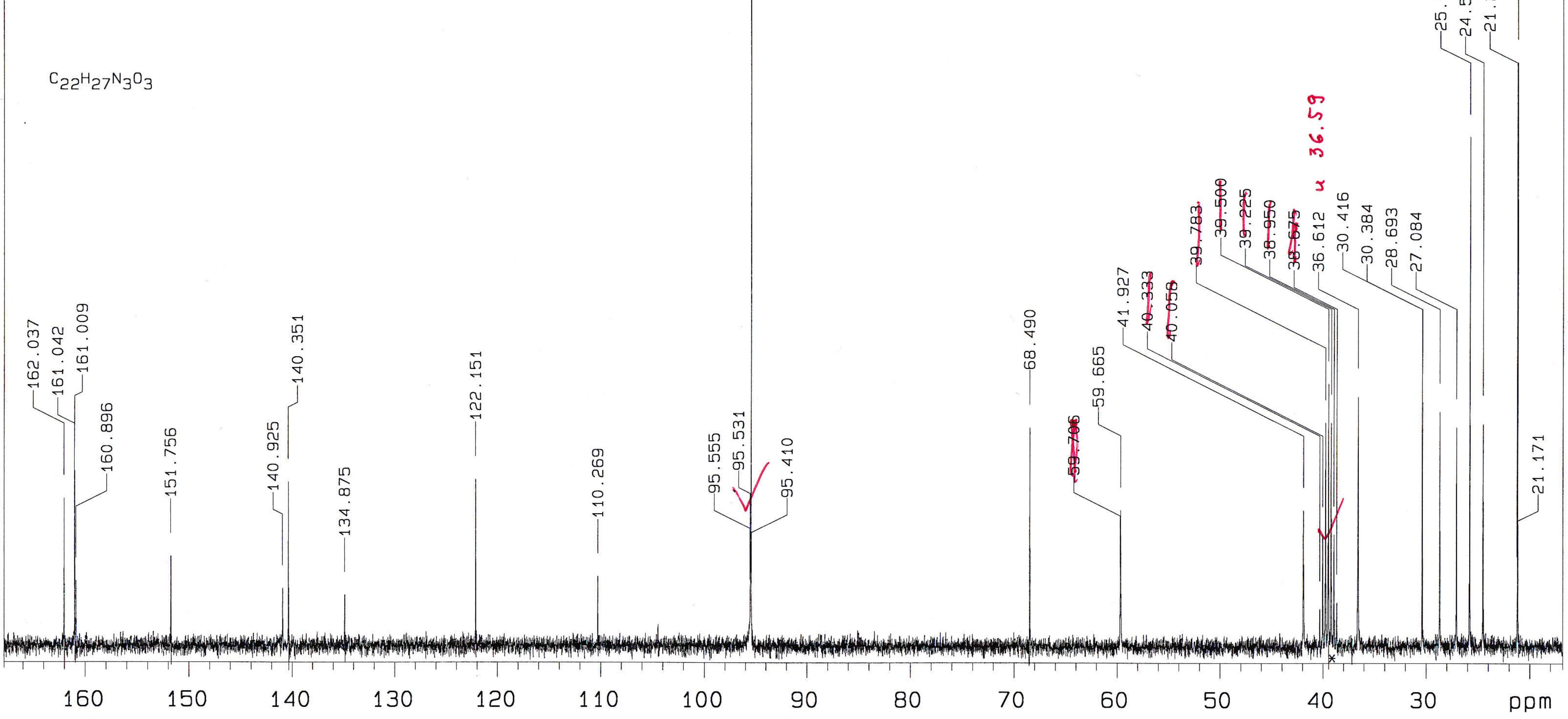

7f

Molecular Structure Research Centre, Yerevan, Armenia, Varian Mercury-300VX

H1 300.088 MHz, nt = 16, np = 32000, temp = 30.0 C, lb = -0.2, solvent = DMSO/CCl4 1/3

May 14 2019

NOCI_19 ha-1041

HA-1041

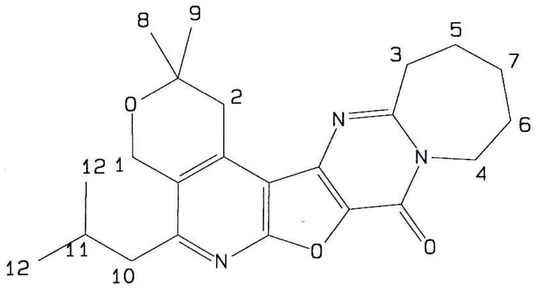

$C_{23}H_{29}N_3O_3$

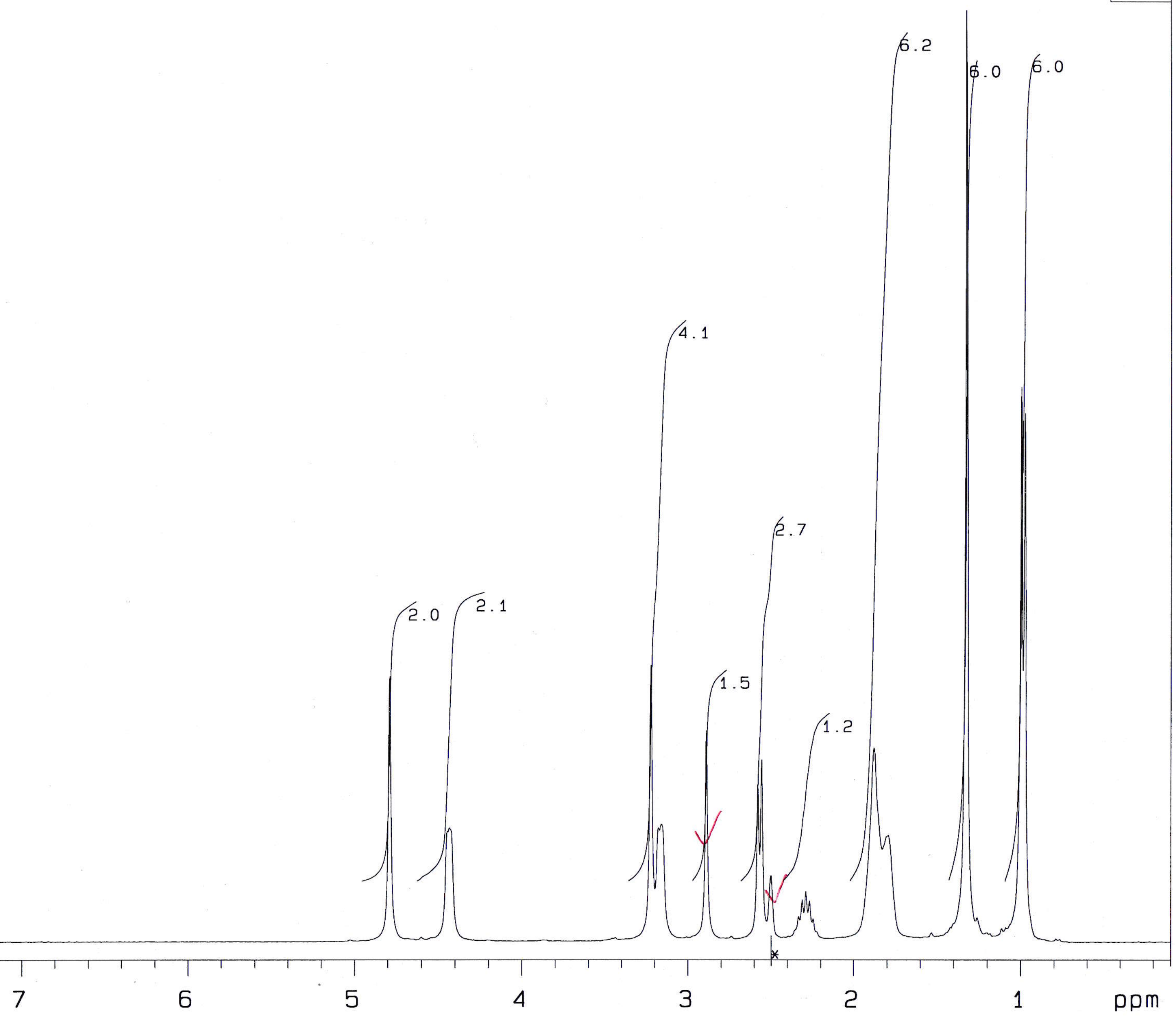

7f

Molecular Structure Research Centre, Yerevan, Armenia, Varian Mercury-300VX

C13 75.465 MHz, nt=512, np=19998, temp=30.0 C, lb=1.0, solvent=DMSO/CCl4 1/3

May 14 2019

NOCI_19 ha-1041

HA-1041

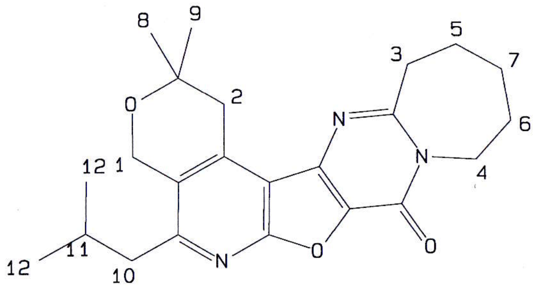

$C_{23}H_{29}N_3O_3$

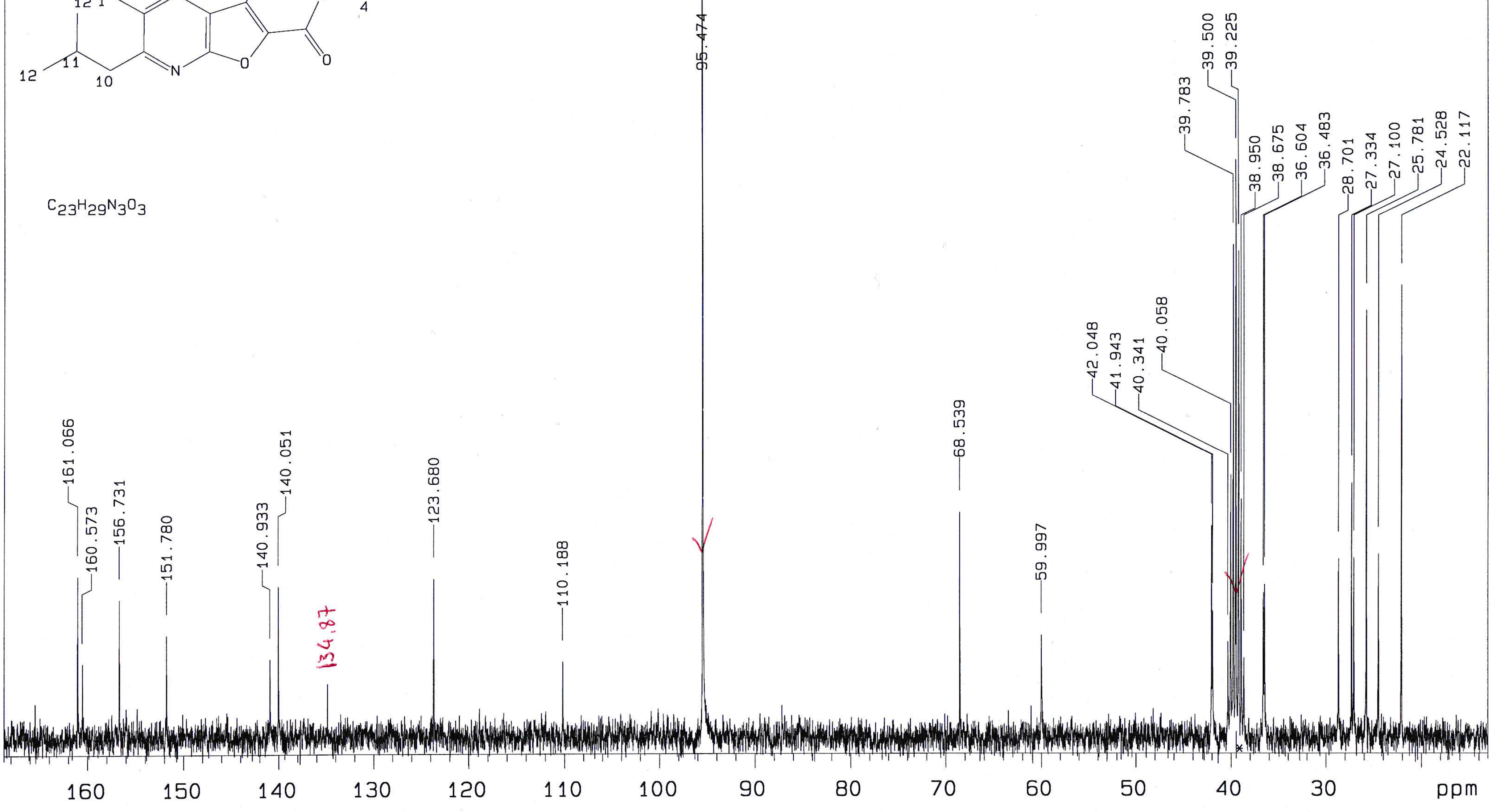

+

7g

Molecular Structure Research Centre, Yerevan, Armenia, Varian Mercury-300VX

H1 300.088 MHz, nt = 16, np = 32000, temp = 30.0 C, lb = -0.2, solvent = DMSO/CCl4 1/3

Feb 21 2017

HA-955

SAMV_17 ha-955

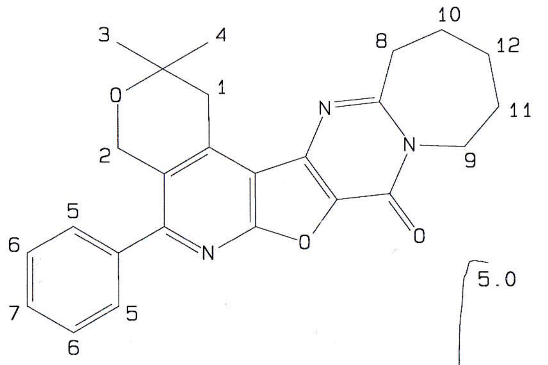

$C_{25}H_{25}N_3O_3$

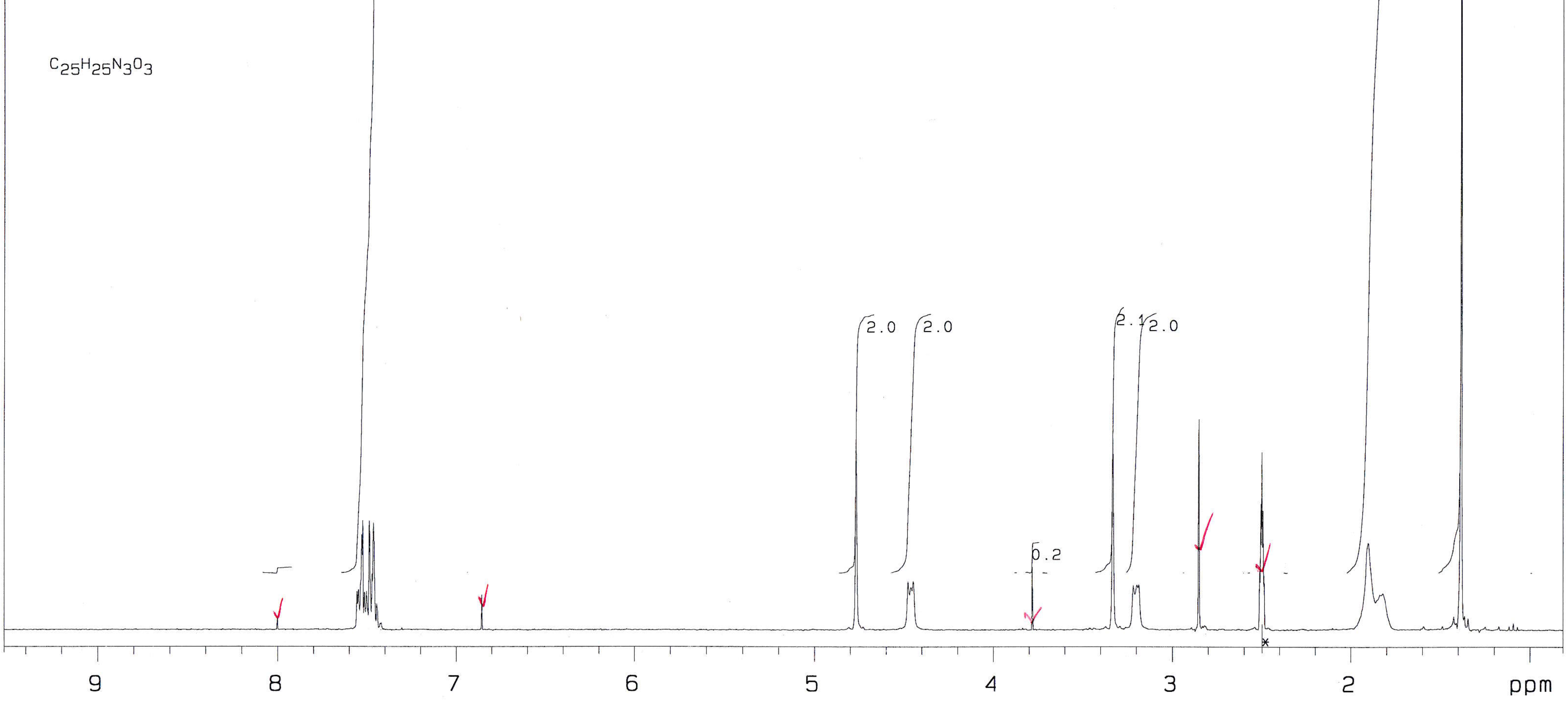

7g

Molecular Structure Research Centre, Yerevan, Armenia, Varian Mercury-300VX

C13 75.465 MHz, nt = 512, np = 19998, temp = 30.0 C, lb = 1.0, solvent = DMSO/CCl4 1/3

Feb 21 2017

SAMV_17 ha-955

HA-955

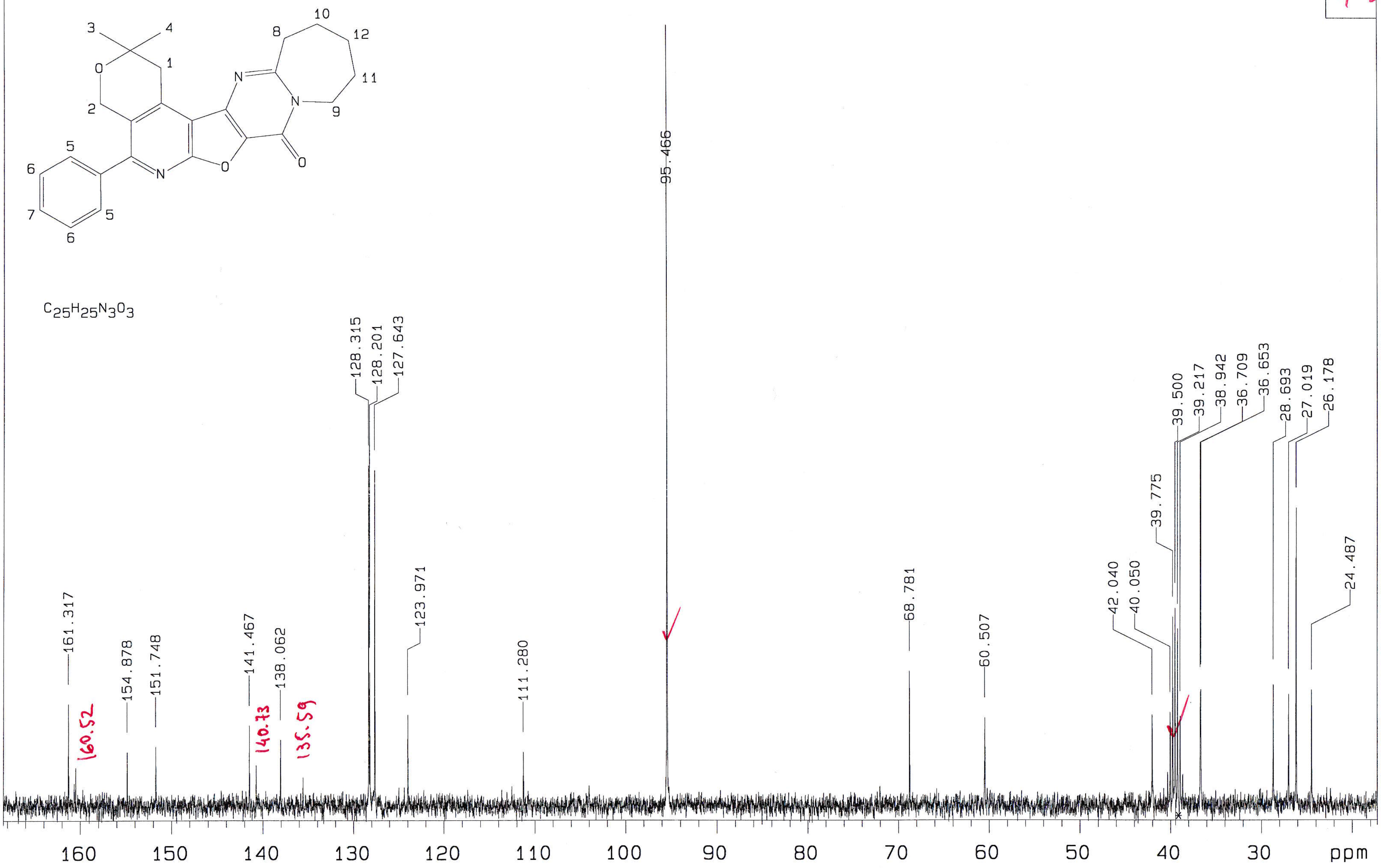

$C_{25}H_{25}N_3O_3$

7h

Molecular Structure Research Centre, Yerevan, Armenia, Varian Mercury-300VX

H1 300.088 MHz, nt = 16, np = 32000, temp = 30.0 C, lb = -0.2, solvent = DMSO/CCl4 1/3

Jan 30 2017

HA-945

SAMV_17 ha-945

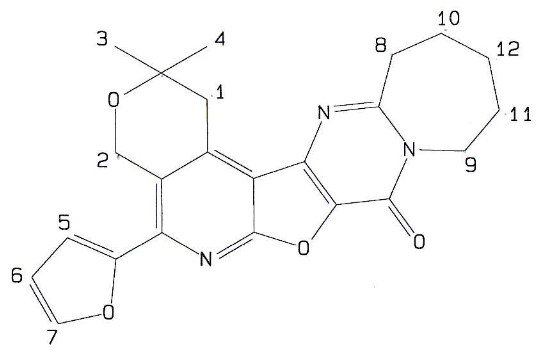

$C_{23}H_{23}N_3O_4$

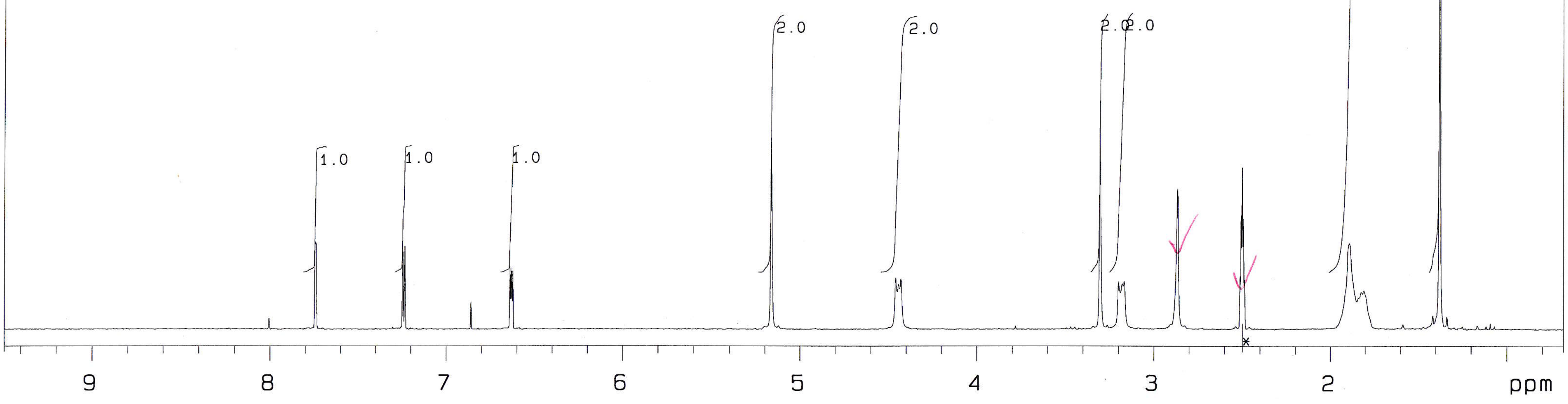

7h

Molecular Structure Research Centre, Yerevan, Armenia, Varian Mercury-300VX

C13 75.465 MHz, nt=704, np=19998, temp=30.0 C, lb=1.0, solvent=DMSO/CCl4 1/3

Jan 30 2017

SAMV_17 ha-945

HA-945

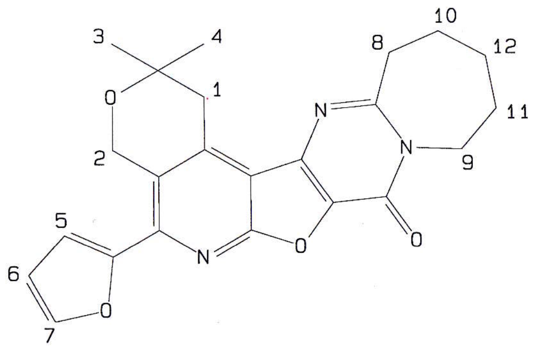

$C_{23}H_{23}N_3O_4$

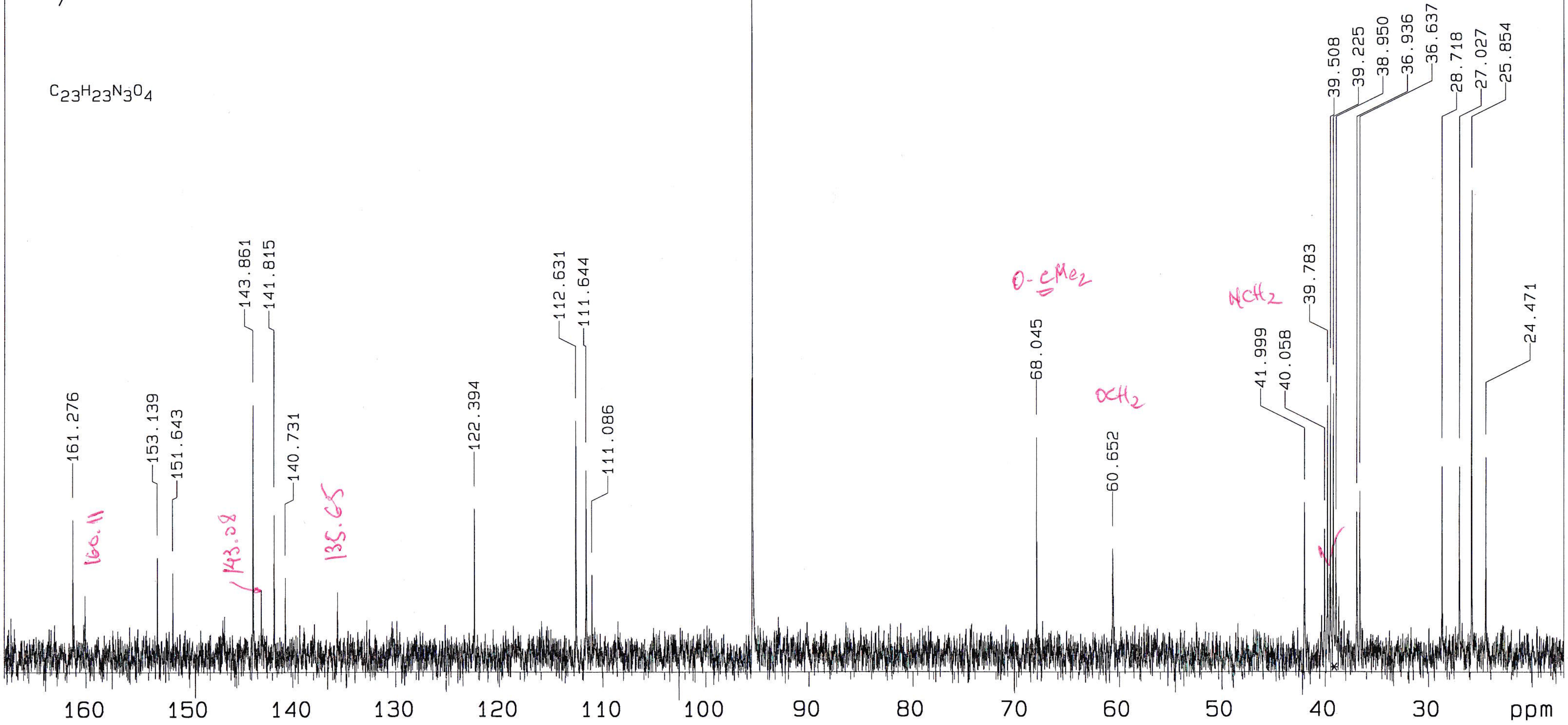

Supplement: Supplementary file 1 [file molecules-26-03320-s001.zip › molecules-1235512-supplementary.pdf]
